# Newly identified motifs in *Candida albicans* Cdr1 protein nucleotide binding domains are pleiotropic drug resistance subfamily-specific and functionally asymmetric

**DOI:** 10.1038/srep27132

**Published:** 2016-06-02

**Authors:** Manpreet Kaur Rawal, Atanu Banerjee, Abdul Haseeb Shah, Mohammad Firoz Khan, Sobhan Sen, Ajay Kumar Saxena, Brian C. Monk, Richard D. Cannon, Rakesh Bhatnagar, Alok Kumar Mondal, Rajendra Prasad

**Affiliations:** 1 School of Life Sciences, Jawaharlal Nehru University, New Delhi, 110067, India; 2 School of Physical Sciences, Jawaharlal Nehru University, New Delhi, 110067, India; 3 The Sir John Walsh Research Institute, University of Otago, Dunedin 9054, New Zealand; 4 School of Biotechnology, Jawaharlal Nehru University, New Delhi, 110067, India

## Abstract

An analysis of *Candida albicans* ABC transporters identified conserved related α-helical sequence motifs immediately C-terminal of each Walker A sequence. Despite the occurrence of these motifs in ABC subfamilies of other yeasts and higher eukaryotes, their roles in protein function remained unexplored. In this study we have examined the functional significance of these motifs in the *C. albicans* PDR transporter Cdr1p. The motifs present in NBD1 and NBD2 were subjected to alanine scanning mutagenesis, deletion, or replacement of an entire motif. Systematic replacement of individual motif residues with alanine did not affect the function of Cdr1p but deletion of the M1-motif in NBD1 (M1-Del) resulted in Cdr1p being trapped within the endoplasmic reticulum. In contrast, deletion of the M2-motif in NBD2 (M2-Del) yielded a non-functional protein with normal plasma membrane localization. Replacement of the motif in M1-Del with six alanines (M1-Ala) significantly improved localization of the protein and partially restored function. Conversely, replacement of the motif in M2-Del with six alanines (M2-Ala) did not reverse the phenotype and susceptibility to antifungal substrates of Cdr1p was unchanged. Together, the M1 and M2 motifs contribute to the functional asymmetry of NBDs and are important for maturation of Cdr1p and ATP catalysis, respectively.

The major role played by the ATP binding cassette (ABC) transporter *Candida albicans* drug resistance protein 1 (Cdr1p) in azole resistance was first deduced from the correlation between its high level of expression in many azole resistant clinical isolates and their rapid efflux of accumulated drugs^1^,^2^,^3^. Cdr1p is thus of clinical importance and has become the target of strategies designed to combat high-level antifungal resistance^4^,^5^.

Cdr1p is an integral plasma membrane (PM) protein of 1501 amino acids and has a predicted molecular weight of 169.9 kDa^1^. It consists of two homologous halves with a domain organisation characteristic of the pleiotropic drug resistance (PDR) subfamily i.e. (nucleotide binding domain [NBD] -transmembrane domain [TMD])_2_^6^ (Fig. 1A). Each TMD contains six α-helical transmembrane segments (TMS) that contribute to multiple overlapping substrate binding sites^5^,7–9. The twelve TMSs are interlinked by six extracellular loops (ECL1-6) and four intracellular loops (ICL1-4) (10; Fig. 1A). The NBDs are the most conserved regions of Cdr1p. They bind and hydrolyze cellular ATP, energizing translocation of a variety of structurally unrelated compounds across the PM^8^,^9^. Due to their common substrate (ATP), NBDs from different ABC proteins share extensive amino acid sequence identity and motifs that are considered to be critical for the domain function^10^. These include a Walker A motif with a consensus sequence GxxGxGKS/T, where ‘x’ represents any amino acid, a Walker B motif hhhhD, where ‘h’ represents any aliphatic residue, and an ABC signature motif LSGGQQ/R/KQR. (Fig. 1B)^11^,^1^2. Comparison of the primary sequences of NBDs of fungal proteins belonging to the PDR subfamily reveals that Cdr1p possesses divergent amino acids in otherwise well conserved motifs. For example, in the Walker A motif (GRPGAGCS) in the NBD1 of Cdr1p the usual lysine residue (K) is replaced by a cysteine (C). In contrast the Walker A of the NBD2 in Cdr1 and other yeast ABC transporters contains the evolutionary conserved lysine (GASGAGKT). The Walker B motif of NBD1 in Cdr1p is also degenerate (IQCWD) when compared with the Walker B of NBD2 (LLFLD) and with other yeast ABC transporter Walker B motifs. Finally, NBD1 possesses a well-conserved ABC signature sequence (VSGGERKRVSIA) but the counterpart in NBD2 is degenerate (LNVEQRKRLTIGV). Research to date has established that the unique evolutionary replacements in Cdr1p are critical for function. For example, biochemical analysis of a purified functional NBD1 domain of Cdr1p revealed that the atypical C193 of the Walker A motif is required for ATP hydrolysis^13^. The swapping of C193 of NBD1 with the conserved K901 of NBD2, in the context of the full protein, was not tolerated and cells expressing the swapped variants displayed very low ATPase activity and high susceptibility to drugs^14^. It has been shown for the closely related ABC transporter Pdr5p that mutations in the divergent ATP binding site did not significantly affect ATPase activity^15^. It has been suggested that the divergent PDR ATP binding site had evolved to adopt new functions and plays an active role in the transport cycle^16^. The domains responsible for nucleotide binding and hydrolysis are composites that use parts of NBD1 and NBD2 and it is not known for fungal PDR ABC proteins whether both composite sites are catalytically active.

Our previous genome-wide inventory of *C. albicans* ABC transporters revealed a pair of sequence motifs characteristic of each subfamily of *C. albicans* ABC proteins (Table 1; ref 17). These motifs occur in both NBDs immediately C-terminal of the Walker A sequences in PDR and MDR subfamilies^17^, which are distinct subfamilies of ABC transporters both of which confer resistance to multiple xenobiotics. Although these new motifs have been used to identify sequences from the corresponding subfamily in other organisms (Table 1) their role in ABC protein function has not been determined. In the present study, we have examined the functional significance of these uncharacterized motifs in Cdr1p. We have shown that these new motifs are functionally asymmetric in both NBDs i.e. the motif in NBD1 is critical for proper localisation or folding of Cdr1p and the motif in NBD2 is required for ATP hydrolysis.

## Results

### Modeling of the M1- and M2-motifs in open and closed Cdr1p structures

**Figure 1:**
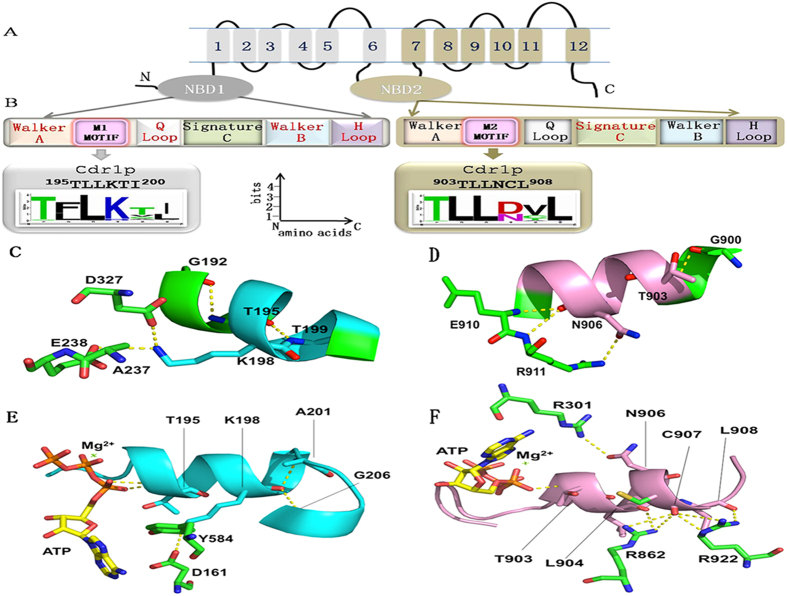
PDR subfamily-specific motif in Cdr1p. (**A**) Schematic representation of Cdr1p topology showing two TMDs each comprising six TMSs preceded by an NBD. (**B**) New motifs which are designated as M1-motif (in NBD1) and M2-motif (in NBD2) occur immediately C-terminal of each Walker A sequences (upper panel). Sequence logos depicting conservation of motif residues across 85 fungal PDR transporters (lower panel). The scale indicates the frequency of a particular amino acid at a given position. The residues at each position are arranged in order of predominance, with the highest frequency residue at the top. The height of symbols within each stack indicates the relative frequency of each amino acid at that position. Green indicates polar residues, blue basic, red acidic, black hydrophobic and violet indicates amino acids that have polar amide groups. Sequence logos were generated using the “WebLogo” (http://weblogo.berkeley.edu/). Alignment of the 85 PDR transporters is provided as Supplementary Alignment 1. Models of consensus M1-motif and M2-motif in both the open (**C,D**) and closed (ATP-bound) Cdr1p structures (**E,F**). Teal and pink colors represent the M1- and M2-motif respectively. Hydrogen bonds between the motif and other atoms are shown as yellow dotted lines. For clarity, the amino acids and ATP molecules making hydrogen bonds with the motifs are shown in different color schemes to the motif and the rest of the molecule. Hydrogen bonds within the motifs are not shown.

We previously used comparative primary sequence alignment to identify among ABC proteins in *C. albicans* two NBD-specific variants of a motif characteristic of the PDR subfamily of ABCG proteins^17^. For the present study these motifs are designated as the M1-motif (TLLKTI) in NBD1 and the M2-motif (TLLNCL) in NBD2 (Fig. 1B, upper panel). The conservation of both these motifs was assessed across 85 fungal PDR transporters having domain organization similar to Cdr1p, i.e. (NBD-TMS_6_)_2_. For this, the sequences of the 85 non-redundant full-length PDR transporters were aligned with Cdr1p using the membrane specific multiple alignments program PRALINE^TM^ and sequence logos (Fig. 1B, lower panel) were generated which indicated that the M1- and M2-motifs are conserved across a range of fungi, with the N-terminal sequence being more conserved than the C-terminal sequence in both the motifs.

We explored the environment of the M1 and M2 motifs using Cdr1p homology models based on the Rutledge *et al.*^18^ homology models of Pdr5p in the open and closed (ATP-bound) conformations. In the open conformation, both motifs (underlined) are part of a 10 amino acid helix, G^192^CSTLLKTIA^201^in NBD1 and G^900^KTTLLNCLS^909^ in NBD2, with the first 3 amino acids in each of these sequences comprising the C-terminal end of a Walker A motif. In the M1-motif the T195 side-chain hydroxyl hydrogen bonds with the main chain carbonyl of G192, the side-chain amino of K198 forms ionic bonds with both E238 and D327 while the main chain nitrogen of A237 caps the helix by hydrogen bonding with the main chain carboxyl of T199 (Fig. 1C). Similarly, in the M2-motif the side-chain hydroxyl of T903 hydrogen bonds with the main chain carboxyl of G900, the side-chain hydroxyl of N906 hydrogen bonds with the side-chain guanidino group of R911 and the main chain nitrogens of E910 and R911 cap the helix by hydrogen bonding to the main chain nitrogen of N906 (Fig. 1D). On the opposite faces of both motif helices hydrophobic (L) and polar residues (T, C) pack against nearby structures including β-sheets. This arrangement is essentially unchanged in both the open and closed conformations of Cdr1p.

In the model of the closed conformation of Cdr1p, the binding of two ATP molecules in the structure truncates both ends of each 10 amino acid helix to give helices consisting of 8 amino acids. Each helix now contains the two C-terminal amino acids of the Walker A sequence plus the motif only (C^193^STLLKTI^200^ in NBD1 and K^901^TTLLNCL^908^ in NBD2). The N-terminal faces of each helix hydrogen bond with the α-phosphate of ATP plus other residues of the Walker A motif while the C-terminal end leads into a helix-capping loop. The closed model of Cdr1p indicates that the conserved T (T195 or T903) hydrogen bonds with the α-phosphate of ATP via its peptide amide in both cases and via the side-chain hydroxyl group for T195 only. The side-chain amino group of K198 forms a non-conserved ionic bond with side-chain carboxyl of D161 (with D213 in a different surface loop in Pdr5p) and a conserved hydrogen bond with the phenol group of Y584 (Y594 in Pdr5p) that is associated with intracellular loop 1 (ICL1) (Fig. 1E). In contrast N906 (D916 in Pdr5p) forms a conserved hydrogen bond with R301 (R316 in Pdr5p), an amino acid residue that lies almost immediately N-terminal of the Cdr1p NBD1 signature sequence (R^301^GVSGGERKRVS, signature sequence underlined) (Fig. 1F). This analysis suggested that the M1-motif and M2-motif might have distinctly different roles in the biogenesis and/or function of Cdr1p. Considering the predicted interaction of the motifs with ATP itself and with structures known to be involved in ATP-mediated catalysis and efflux, a systematic mutagenesis strategy was employed to assess their impact on the localization and function of the *C. albicans* Cdr1p ABC transporter.

### Alanine scanning mutagenesis of the motifs does not affect cellular localization of Cdr1p or susceptibility to xenobiotic substrates

**Figure 2:**
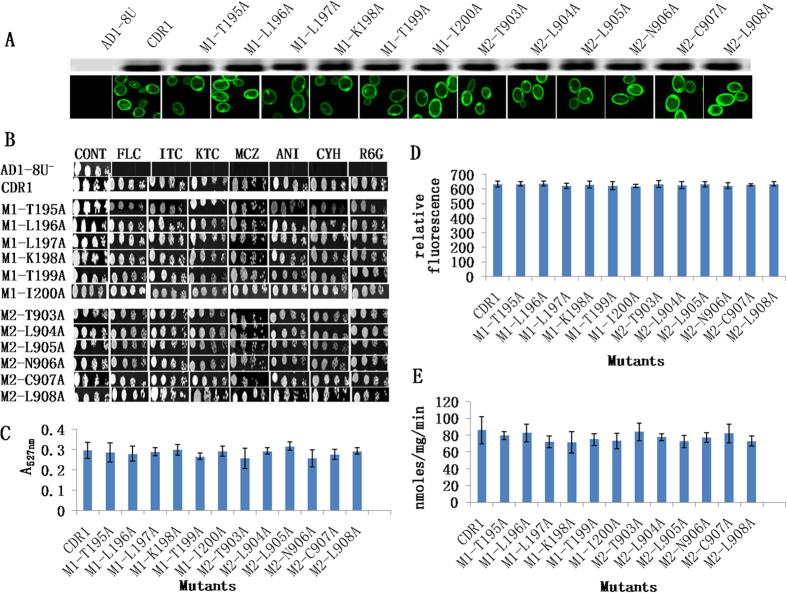
Alanine scanning mutagenesis of M1-motif and M2-motif. (**A**) Western blot and confocal microscopy images showing expression and membrane localization of the WT and alanine mutant variants of both the M1-motif and M2-motif. Immunodetection of GFP-tagged Cdr1p and its variants was performed using HRP-labeled anti-GFP antibody. (**B**) The drug resistance profiles of yeast strains overexpressing WT Cdr1p or mutant variants of the M1-motif and M2-motif were determined using agarose-based drug susceptibility assays as described in Methods section. (**C**) R6G transport (**D**) NR transport and (**E**) ATPase activities of M1-motif and M2-motif mutant variants. R6G, NR and Mg-ATP were used at a final concentration of 10 μM, 7 μM and 5 mM, respectively, for measurement of drug efflux and ATPase activities. Energy dependent R6G efflux was quantified by measuring the absorbance of the supernatant at 527 nm as described in Methods section. Results are means of at least 3 independent experiments. Error bars represent standard deviation. Differences between the mean values were analyzed by Student’s t-test.

The function of the M1- and M2-motifs in Cdr1p-GFP was tested initially using scanning mutagenesis in which each amino acid residue of the motifs was replaced with an alanine (Fig. 2). All the mutant ORFs were integrated at the genomic *PDR5* locus in a *Saccharomyces cerevisiae* AD1-8u^−^ heterologous hyper-expression system^19^. The host AD1-8u^−^ was derived from a *pdr1–3* mutant strain with a gain-of-function mutation in the transcription factor Pdr1p that result in constitutive hyper-induction of the *PDR5* promoter^19^. The host strain is also deleted of seven ABC transporters plus the *PDR3* gene that encodes the Pdr3p transcriptional regulator. This gives a strain that is hypersensitive to xenobiotic substrates of Cdr1p and contributes low levels of background activity in efflux and ATPase assays when Cdr1p is over-expressed. Confocal and immunblot analysis of the PM fractions with an anti-GFP monoclonal antibody confirmed that Cdr1-GFP was stably overexpressed and correctly localized to the plasma membrane. All site-directed mutations were confirmed by sequencing the entire *CDR1* ORF. Confirmed mutant variants were also assessed for the expression of Cdr1p fused with GFP (Cdr1p-GFP). Western blot analysis showed the wild-type (WT) and mutant Cdr1p proteins were expressed at similar levels in the PM. In addition, the confocal images of GFP-tagged Cdr1p confirmed that WT and mutant proteins were localized comparably at the cell surface (Fig. 2A). The drug susceptibilities of the cells expressing WT Cdr1p-GFP were compared with those expressing its variants using two independent drug susceptibility assays. Agarose-based drug susceptibility assays and liquid MIC_80,_(minimum concentration resulting in >80% growth inhibition compared with the no drug control) values were determined for each mutant as described in the Methods section^20^. The agarose-based drug suceptibility assays showed that the replacement of individual residues of the motif with alanine had little or no effect on drug susceptibility compared with WT Cdr1p (Fig. 2B). These results were also confirmed by the MIC_80_ determinations where the values of WT Cdr1p and mutant variants were comparable (Supplementary Table S1). We compared the the transport capability of WT Cdr1p and its mutant variants with an R6G efflux assay and flow cytometry-based NR accumulation as described in the Methods section. The efflux activity of all mutant variants was comparable with those for cells overexpressing WT Cdr1p, as shown in Fig. 2C,D. The oligomycin-sensitive ATPase activity of isolated plasma membranes from WT and mutant Cdr1-GFP variants, analysed as described in the Methods section, was found to be comparable for the WT and mutant strains. Collectively, the confocal microscopy, the drug susceptibility profiles, the drug efflux results and the Cdr1-ATPase activities of all the mutant variants GFP (Fig. 2C–E) showed that alanine replacement of individual residue in the Cdr1p motifs did not not affect cellular localization, enzyme activity, pump function, or resistance to the xenobiotic Cdr1p substrates tested.

### Deletion of the motifs increased drug susceptibility and caused mislocalization of M1-Del Cdr1p

**Figure 3:**
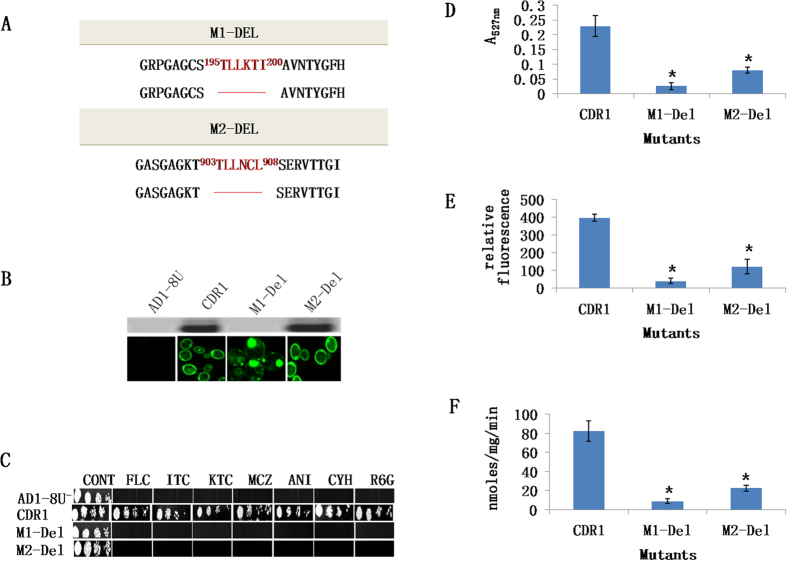
Deletion of M1-motif or M2-motif. (**A**) The strategy for constructing variants deleted of the entire NBD1 motif (M1-Del) or NBD2 motif (M2-Del). Deletions are represented by red lines. (**B**) Western blot and confocal microscopy images showing expression in a PM fraction and overall cellular localization of WT, M1-Del and M2-Del Cdr1p-GFP. Immunodetection of GFP-tagged Cdr1p and its variants was performed using HRP-labeled anti-GFP antibody. (**C**) Drug resistance profile of yeast strains overexpressing WT Cdr1p and deletion mutants M1-Del and M2-Del were determined using agarose-based drug susceptibility assays as described in Methods section. (**D**) R6G transport, (**E**) NR transport and (**F**) ATPase activities of WT and M1-Del and M2-Del Cdr1p variants. Efflux and ATPase activities were determined as described in Fig. 2. Mutant variants showing a significant difference compared to WT Cdr1p are marked with an asterisk (*). Results are means of at least 3 independent experiments. Error bars represent standard deviation.

Since the replacement of single residues in motifs with alanine did not yield a phenotype, the entire motif in each NBD was deleted (Fig. 3A). Confocal microscopy of the cells expressing mutant Cdr1p-GFP found that the removal of the NBD1 motif (M1-Del) led to mislocalization of the protein to an internal site likely to be the endoplasmic reticulum (ER). In contrast, deletion of the NBD2 motif (M2-Del) gave proper localization of Cdr1p-GFP to the PM (Fig. 3B, lower panel). Western blot analysis showed there was little or no expression of the M1-Del protein in the PM but WT levels of the M2-Del protein were detected in this fraction (Fig. 3B, upper panel). Cells expressing either the M1- or M2-deleted variants showed greater susceptibility to all drugs tested (Fig. 3C). The cells showed significantly reduced efflux of the Cdr1p substrates R6G and NR and the PM lacked the ATPase activity normally be attributable to overexpression of Cdr1p-GFP (Fig. 3D–F).

### Alanine replacement of motif partially restores Cdr1p function

**Figure 4:**
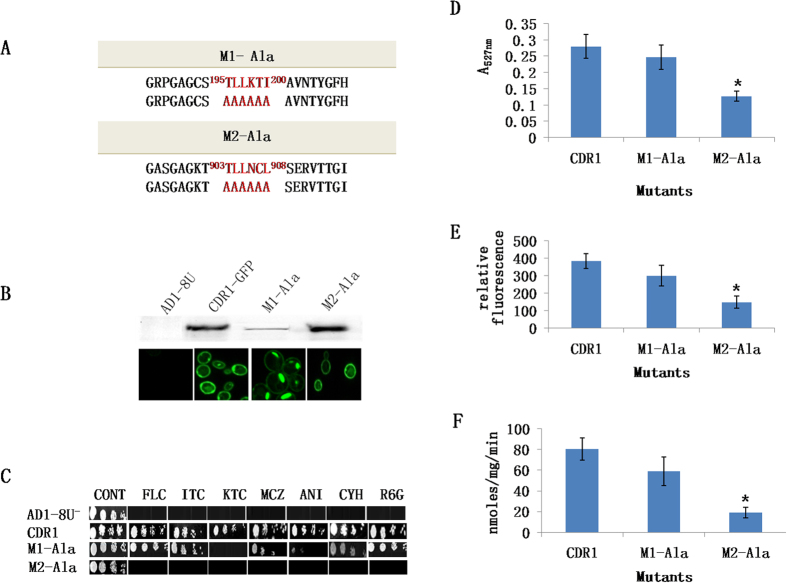
Replacement of M1-motif or M2-motif with hexa-alanine insertion. (**A**) The strategy for constructing hexa-alanine replacement mutants of the entire motif in NBD1 (M1-Ala) and in NBD2 (M2-Ala). Insertions are represented by hexa-alanine insertions (in red). (**B**) Western blot and confocal microscopy images showing expression and membrane localization of the WT, M1-Ala and M2-Ala GFP-tagged Cdr1p. Immunodetection of GFP-tagged Cdr1p and its variants was performed using HRP-labeled anti-GFP antibody. (**C**) Drug resistance profiles of yeast strains overexpressing WT and M1-Ala and M2-Ala Cdr1p were determined using agarose-based drug susceptibility assays as described in Methods section. (**D**) R6G transport, (**E**) NR transport and (**F**) ATPase activities of WT and M1-Ala and M2-Ala mutant variants. Efflux and ATPase activities were determined as described in Fig. 2. Mutant variants showing a significant difference compared to WT Cdr1p are marked with an asterisk (*). Results are means of at least 3 independent experiments. Error bars represent standard deviation.

We speculated that restoration of motif α-helical structure with alanines might correct the mislocalization of M1-Del Cdr1p-GFP and restore drug resistance and efflux activity. The deleted stretches in both M1-Del and M2-Del were replaced with an equivalent number of alanines (hexa-alanine) and the resulting variants of Cdr1p-GFP designated as M1-Ala and M2-Ala, respectively (Fig. 4A). The variant M1-Ala partially localised (~25%) to the PM (Fig. 4B) and substantially restored resistance to some but not all drugs i.e. FLC, ITC MCZ and CYH but not KTC or ANI (Fig. 4C). ATPase and efflux activities were also restored to at least 60% of control levels (Fig. 4D–F). In contrast, the M2-Ala did not show the WT drug resistance phenotype. Cells overexpressing M2-Ala remained susceptible to drugs (Fig. 4C), failed to efflux R6G and NR (Fig. 4D,E) and lacked significant Cdr1p-GFP dependent ATPase activity (Fig. 4F).

**Figure 5:**
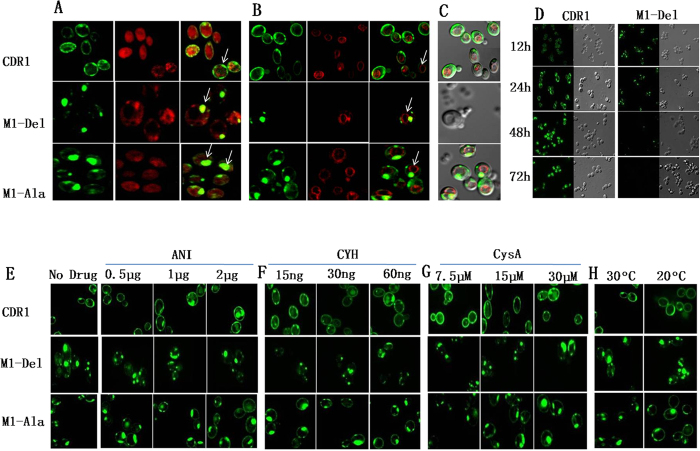
M1-Ala but not M1-Del entrapped within the ER can relocate to the PM by growth in the presence of chemical chaperones and at low temperature. (**A**) Staining of cells expressing WT Cdr1p-GFP, M1-Del and M1-Ala variants with the ER-specific dye ER-Tracker. The confocal images for individual strains consist of three subpanels, representing fluorescence due to GFP (left panel), fluorescence due to ER-Tracker (middle panel) and their superimposition (right panel). M1-Del is entrapped in the ER (yellow color indicated by arrows). About 75% of M1-Ala was entrapped within the ER, with only ~25% associated with the PM (a faint green color outlining cortical ER). WT Cdr1p showed a prominent green color rim (indicated by arrow) surrounding the red color of the cortical ER, consistent with its PM localization. (**B**) Staining of cells expressing WT Cdr1p-GFP, M1-Del and M1-Ala variants with the vacuolar membrane dye FM4-64. The confocal images for individual strains consist of three subpanels, representing fluorescence due to GFP (left panel), fluorescence due to FM4-64 (middle panel) and their superimposition (right panel). FM4-64 stains vacuolar membranes. M1-Del, M1-Ala are clearly shown to be present outside the vacuolar membrane (the vacuolar membrane is indicated by an arrow) similar to WT CDR1. (**C**) DIC image showing FM4-64 staining, GFP tagged protein variants and vacuole *per se* confirms the localization of protein variants outside the vacuole (probably in the ER as shown by colocalization with ER-tracker dye). (**D**) M1-Del mutant protein was unstable and disappeared after 72 h growth, unlike Cdr1p-GFP. (**E,F,G**) Relocation of ER-entrapped M1-Del and M1-Ala to the cell surface during growth in the presence of substrates and chaperones. Substrates of Cdr1p (at sub-MIC concentration) such as anisomycin (0.5 μg, 1 μg, and 2 μg), cycloheximide (15 ng, 30 ng, and 60 ng) and the pharmacological chaperone cyclosporin A (CysA, 7.5 μM, 15 μM, and 30 μM) were added to M1-Del and M1-Ala cells just after lag phase (4 to 5 h growth, OD_600_ of ~1). The cells were grown for 12 h and the localization Cdr1p-GFP assessed by confocal microscopy. (**H**) Growth of M1-Ala but not M1-Del at 20 °C improves Cdr1p-GFP localization to the PM.

The localization of protein variants to the ER was confirmed by monitoring co-localization with the ER-specific dye ER-tracker in cells expressing WT Cdr1p-GFP, M1-Del and M1-Ala variants (Fig. 5A). This result was validated by excluding trafficking to the vacuole using the fluorescent vacuole-specific dye FM4-64, which stains vacuolar membranes (Fig. 5B). The staining with FM4-64 showed clearly that M1-Del and M1-Ala variant proteins were outside the vacuole i.e. the GFP-tagged variant proteins were outside the red boundary of vacuolar membrane that encloses the large central vacuole (Fig. 5B). Superimposed differential interference contrast (DIC) images of FM4-64 staining, GFP-tagged protein variants and the vacuole *per se* also demonstrated that the protein variants were localized outside the vacuole (Fig. 5C). These results confirmed that the M1-Del variants were trapped within the ER network. Deletion of the M1-motif (M1-Del) appeared to trap Cdr1p-GFP in the ER quality control network and prevented it from reaching its normal target, the PM. It was eventually degraded in stationary phase cells after 72h, unlike cells expressing WT Cdr1p-GFP (Fig. 5D). While M1-Del Cdr1p-GFP remained entrapped in the ER until degraded under all conditions tested, the entrapped component of M1-Ala Cdr1p-GFP was partially rescued with chemical chaperones and at lower temperature (Fig. 5E–H). These results suggest that the efficient translocation of Cdr1p-GFP to the PM may require formation of the complete M1-containing α-helix.

### Dissection of motifs reveals importance of entire M1-motif as compared to ^903^TLL^905^ of M2-motif

**Figure 6:**
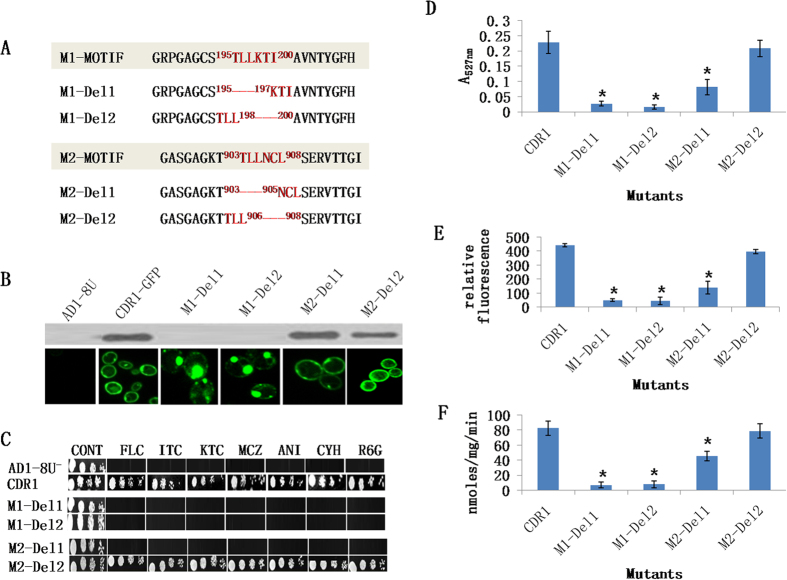
Partial deletion of M1-motif or M2-motif. (**A**) The strategy for constructing M1-Del1 and M1-Del2 and M2-Del1 and M2-Del2. Deletions are represented by red lines. (**B**) Western blot and confocal microscopy images of the WT, M1-motif and M2-motif deletant partners showing the expression and membrane localization of GFP-tagged Cdr1p. GFP-tagged Cdr1p and its variants were immunodetected using HRP-labeled anti-GFP antibody. (**C**) Drug resistance profiles of yeast strains overexpressing WT, M1-motif and M2-motif deletant Cdr1p were determined using agarose-based drug susceptibility assays as described in Methods section. (**D**) R6G transport, (**E**) NR transport and (**F**) ATPase activities of WT, M1-motif and M2-motif deletant proteins. Efflux and ATPase activities were determined as described in Fig. 2. Mutant variants showing a significant difference compared to WT Cdr1p are marked with an asterisk (*). Results are means of at least 3 independent experiments. Error bars represent standard deviation.

Portions of each motif were deleted to evaluate their contribution to the function of the entire motif. The M1-motif was divided into M1-Del1 (ΔT^195^LL^197^) and M1-Del2 (ΔK^198^TI^200^) while the M2-motif was divided into M2-del1 (ΔT^903^LL^905^) and M2-Del2 (ΔN^906^CL^908^), as shown in Fig. 6A. Both M1-motif deletants (M1-Del1 and M1-Del2) gave mislocalized Cdr1p-GFP (Fig. 6B) implying that both parts of the M1-motif may be important for correct localization of Cdr1p-GFP.

Only the first three amino acids of the M2-motif appeared to be critical for function. Thus, only M2-Del1 (ΔT^903^LL^905^) showed increased susceptibility with all drugs tested (Fig. 6C), with drug efflux reduced and with retention of only 54% of WT Cdr1p-GFP ATPase activity (Fig. 6D–F). In contrast, M2-Del2 (ΔN^906^CL^908^) showed behaviour comparable with WT Cdr1p-GFP (Fig. 6B–F).

**Figure 7:**
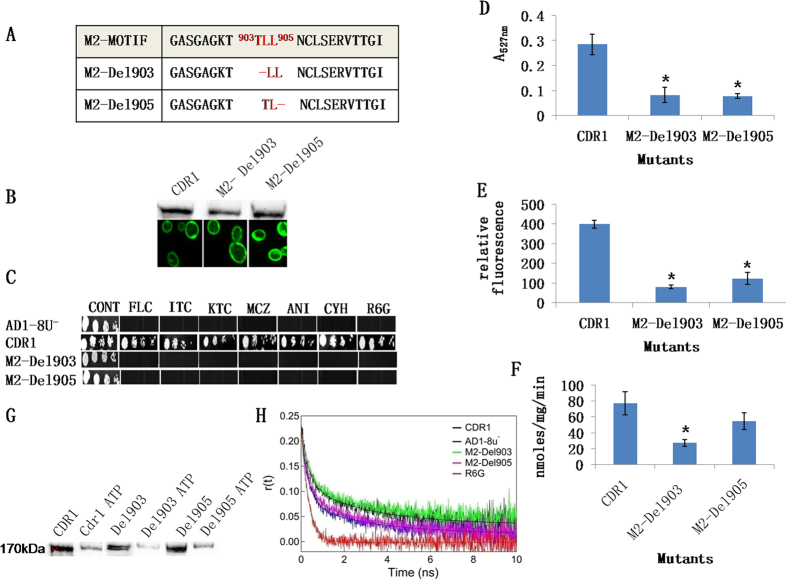
Deletion of individual amino acids from the N-terminal half of the M2-motif. (**A**) The strategy for constructing the two deletants M2-Del903 and M2-Del905. Deletions are represented by red lines. (**B**) Western blot and confocal microscopy images showing expression and membrane localization of the WT and M2-motif deletants GFP-tagged Cdr1p. GFP-tagged Cdr1p and its variants were detected using HRP-labeled anti-GFP antibody. (**C**) Drug resistance profile of yeast strains overexpressing WT Cdr1p and M2-motif deletant partner proteins determined using agarose-based drug susceptibility assays as described in Methods section. (**D**) R6G transport, (**E**) NR transport and (**F**) ATPase activities of WT and M2-motif deletant proteins. Efflux and ATPase activities were determined as described in Fig. 2. Mutant variants showing a significant difference compared to WT Cdr1p are marked with an asterisk (*). Results are means of at least 3 independent experiments. Error bars represent standard deviation. (**G**) Nucleotide binding of WT, M2-Del903 and M2-Del905 Cdr1 proteins. PM fractions were incubated with ATP-agarose beads; bound protein was eluted in SDS sample buffer and examined using immunoblotting with anti-GFP monoclonal antibody as described in Methods section. (**H**) Anisotropy decay rate of R6G bound to WT, M2-Del903, M2-Del905 and AD1–8u^−^. The anisotropy decay curve for M2-Del903 (green curve) lies close to native Cdr1p (black curve) while that for M2-Del905 (pink curve) lies close to host AD1–8u^−^ (blue curve).

Since the residues deleted in M2-Del1 appeared critical (Fig. 6C) T903 and L905 were deleted separately to identify the critical residue(s) (Fig. 7A). L904 did not need to be deleted as this residue has the same codon as L905 and its deletion gives the same Cdr1p sequence as deletion of L905. As expected, both M2-Del903 and M2-Del905 were correctly localized to the PM. M2-Del903 Cdr1p-GFP lacked significant ATPase activity but M2-Del905 Cdr1p-GFP retained 71% activity of the WT activity. However, both constructs failed to efflux the drugs tested (Fig. 7B–F).

To test for side-effects due to the deletion of part of the protein primary sequence, the residues deleted in M1-Del1, M1-Del2 and M2-Del1 variants were replaced with alanines. M2-Del1 was not considered for alanine replacement as this deletion did not alter the functional properties of the protein. The three new mutants were designated as M1-Ala1 (^195^TLL^198^ to ^195^AAA^198^), M1-Ala2 (^198^KTI^200^ to ^198^AAA^200^) and M2-Ala1 (^903^TLL^905^ to ^903^AAA^905^). Alanine replacement of the deleted residues in M1-Ala1 did not correct the localization defect of its deletion counterpart M1-Del1 and the alanine replacement mutant remained susceptible to all drugs tested. Similarly, M2-Ala1 showed the same drug susceptibility profile as M2-Del1. The phenotypes of M1-Del1 and M2-Del1 were therefore unlikely to be due to side-effects caused by missing secondary structure and instead indicated the primary sequences had functional significance. In contrast, alanine replacement in M1-Ala2 reversed the localization defect of the M1-Del2 deletant (Supplementary Fig. S1). This suggested that the localization defect of M1-Del2 might be a side-effect of the deletion. While M1-Ala2 developed resistance to 5 of the 7 efflux pump substrates tested, it also remained susceptible to 2 substrates (KTC and ITC). This implied that the deleted sequence was required for aspects of drug efflux.

### M2-Del903 and M2-Del905 deletions affect the kinetics of drug transport

Kinetic analysis of ATPase and drug efflux activities was used to investigate the basis of the M2-Del903 and M2-Del905 phenotypes. When compared to WT Cdr1p-GFP, the ATPase activity of M2-Del903 was reduced 65%. This was accompanied by a high Km and low Vmax compared to the WT construct (Table 2). In contrast, M2-Del905 exhibited near normal ATPase activity with Km and Vmax values similar to those for WT Cdr1p-GFP (Table 2).

An ATP-agarose binding assay^21^ was used to measure the affinity of Cdr1 proteins for ATP. PM proteins bound to ATP-agarose beads were eluted in SDS sample buffer and quantified by immunoblotting with an anti-GFP monoclonal antibody. Control Cdr1-GFP and its M2-Del905 variant were eluted in comparable amounts from the ATP-agarose beads while 60% less of M2-Del903 variant was eluted (Fig. 7G). This result suggests weaker binding of the M2-Del903 variant to ATP beads, consistent with its higher Km values obtained with the ATPase assay.

Comparison of the drug transport kinetics of cells overexpressing WT Cdr1-GFP, M2-Del903 and M2-Del905 found that the two variants transported R6G at about one third the rate of the Cdr1p-GFP construct. The M2-Del903 variant had an R6G binding affinity (measured as Kd) comparable to the WT control, while the higher Kd of M2-Del905 showed a significantly reduced affinity for R6G (Table 2).

### Time-resolved fluorescence spectroscopy (TRFS) reveals decreased substrate binding to M2-Del905

Since M2-Del903 failed to efflux R6G, caused by impaired ATPase activity due to weak ATP binding, while M2-Del905 had a reduced apparent affinity for R6G, we assessed whether the altered drug efflux in these M2-Motif mutants was due to modified interaction with the substrate. For this, time-resolved anisotropy decay measurements were used to characterise the interaction of R6G with Cdr1p, M2-Del903 and M2-Del905. The anisotropy decay of R6G to native Cdr1p was defined by comparing of free and bound R6G. Free R6G in solution rotates rapidly, resulting in a fast decay of *r*(*t*) (Methods section, equation 1) that can be fitted with a single rotational time constant of 345 ps (Table 3 and Fig. 7H). A complex with R6G bound to native Cdr1p rotates more slowly and the *r*(*t*) decay can be fitted with two rotational time constants: a fast component that correlates with the time constant of free R6G (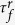 = 345 ps) and a slow component due to R6G bound to Cdr1p (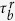 = 3.1 ns) (Table 3). As shown previously^5^ the anisotropy decay of R6G bound to PM containing Cdr1p-GFP has a higher contribution from bound fraction of R6G as well as higher residual anisotropy compared to that in the PM form of the null host AD1-8u^−^ which lacks Cdr1p (Table 3 and Fig. 7H). As can be seen from Fig. 7H and Table 3, samples of R6G incubated with PM containing native Cdr1p-GFP has less free R6G contribution (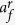 = 0.51) compared to that when R6G was incubated with the PM of AD1-8u^−^ cells (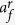 = 0.66). Because of the larger bound fraction (and residual anisotropy) of R6G the raw anisotropy decay data for the PM containing Cdr1p–GFP also showed a slower decay rate. The anisotropy decay curve of Cdr1p-GFP runs above those for free R6G and for R6G with the null host (AD1-8u^−^) PM (Fig. 7H) that is consistent with the fact that binding of substrate is more pronounced in Cdr1p compared to the null. The total fraction, 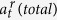, of R6G bound to Cdr1p-GFP was substantially higher (0.49) than for the null host sample (0.34) (Table 3). The background binding observed with the PM from the null host probably reflected the non-specific binding of R6G to other PM proteins. M2-Del905 showed a R6G free fraction (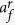 = 0.61) which is comparable to the null host while M2-Del903 showed a free fraction (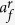 = 0.48) which is comparable to the native Cdr1p-GFP (Table 3). This result was also reflected in their raw anisotropy decay curves (Fig. 7H). The overall faster anisotropy decay of R6G bound to M2-Del905 (with a lower fraction of bound R6G and lower residual anisotropy) lay closer to the null host and confirmed a defect in R6G binding in this variant. In contrast, the overall anisotropy decay of R6G bound to M2-Del903 PM was slower having a higher fraction of bound R6G and higher residual anisotropy. In fact, its anisotropy decay curve lay close to that for native Cdr1-GFP, suggesting R6G interacted comparably with the M2-Del903 protein (Fig. 7H).

To validate the binding of R6G to the protein, we measured the anisotropy decays of R6G (3 μM) in presence of different concentrations of Cdr1p. The anisotropy decay of free R6G shows very fast single exponential decay, while the decays develop bi-exponential feature with increase in the fraction of the slower component (3.1 ns) with the addition of Cdr1p. The data were fitted with the same *r*(*t*) expression, keeping the time-constants of *free* and *bound* R6G same as above. Finally, the *free* and *total bound* fractions were extracted and plotted in Supplementary Fig. S2. The increase in *bound* fraction and the concomitant decrease in *free* fraction of R6G with increase in Cdr1p concentration directly validate the binding of R6G to the protein. In fact, at the highest ratio of [Cdr1p]/[R6G] (=10) we observe the saturation of binding of R6G to Cdr1p.

### M1- and M2-motifs in other other ABC proteins

**Figure 8:**
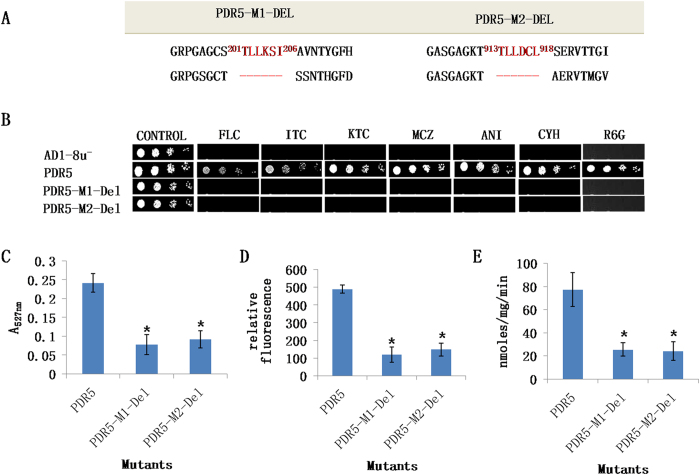
PDR subfamily specific motif in Pdr5p. (**A**) Strategy for constructing Pdr5p variants deleted of the entire NBD1 M1-motif (PDR5-M1-Del) or NBD2 M2-motif (PDR5-M2-Del). Deletions are represented by red lines. (**B**) Drug resistance profiles of yeast strains overexpressing WT Pdr5p-GFP and deletion mutants were determined using agarose-based drug susceptibility assays as described in Methods section. (**C**) R6G transport, (**D**) NR transport and (**E**) ATPase activities of WT and mutant variants. Energy dependent R6G efflux was quantified by measuring the absorbance of the supernatant at 527 nm as described in Methods section. Mutant variants showing a significant difference compared to WT Pdr5p are marked with an asterisk (*). Results are means of at least 3 independent experiments. Error bars represent standard deviation.

The M1- and M2-motifs characteristic of each *C. albicans* ABC transporter subfamily were identified in a previous study^17^. Such motifs can be identified in ABC transporters of other fungi. For example, the PDR (ABCG) subfamily-specific motif is present not only in Cdr1p of *C. albicans* and Pdr5p of *S. cerevisiae* but also in other non-*albicans Candida* species (e.g. *C. dubliniensis, C. glabrata and C. krusei*) and other fungal genomes such as *Aspergillus nidulans, Magnaporthe grisea and Penicillium digitatum* (Table 1). To demonstratethe broader significance of these motifs we tested the effect of deleting the motifs from *S. cerevisiae* Pdr5p. The deletion of either motif yielded a non-functional enzyme (Fig. 8).

Variants of the M1- and M2-motif occur in other subfamilies of ABC transporters. For example MDR (ABCB1) and MRP (ABCC1) subfamily-specific motifs are also found within the NBDs of human ABC proteins such as P-glycoprotein (P-gp) and multidrug resistance protein (Mrp1), respectively. While, the PDR- and MDR-subfamily specific motifs occur in both their NBDs immediately C-terminal of the Walker A sequences, the MRP subfamily specific motif is an extension of Walker B in NBD1 and while in NBD2 it lies just N-terminal of the Q loop (Table 1). This report provides the first experimental exploration of the function of the M1- and M2-motifs despite their wider occurrence in ABC transporter subfamilies beyond the PDR family.

## Discussion

The functional characterization of a pair of NBD motifs (M1, M2) that are conserved among fungal ABC transporters in the PDR subfamily is highlighted. Our experiments demonstrated the functional importance of the M1 and M2 motifs and that they contribute to the functional asymmetry of the NBDs.

Deletion of the M1-motif (M1-Del) trapped the mutant Cdr1p in the ER quality control network. The mutant protein was eventually degraded in stationary phase cells after 72h, unlike WT Cdr1p-GFP which was retained in the PM. Deletion of either the N-terminal or C-terminal half of the M1-motif also led to mis-localization to the ER. In contrast, scanning alanine mutagenesis showed that modification of individual side-chains in the M1-motif did not noticeably affect Cdr1p function. Furthermore, the M1-Ala variant of Cdr1p, which contains hexa-alanine in place of the M1-motif, gave partial rescue of enzyme mis-localization, with ~25% of Cdr1p-GFP observed in the PM and 77% of Cdr1p-specific ATPase activity detected in enriched PM fractions. Cdr1p-specific NR and R6G efflux was also rescued to ~78% and ~85% of WT levels, respectively. The PM localisation of Cdr1p-GFP was further improved in the M1-Ala but not the M1-Del mutant, using either chemical chaperones or growth at alower temperature (20 °C). These results imply that translocation to the PM may depend on formation of the M1-containing α-helix, including the ability of the T195 polypeptide backbone amino group to hydrogen bond with a Walker A motif carbonyl in the open conformation and/or with the α-phosphate of ATP in the closed conformation of Cdr1p-GFP. The improvement in protein localisation by chemical chaperones, such as the Cdr1p substrates anisomycin and cycloheximide, suggests protein stabilisation via the formation of a substrate-stimulated closed conformation.

Our models suggest that hydrogen-bond interactions involving the Walker motif main chain carbonyls or the α-phosphate of ATP may be further stabilised by a non-essential hydrogen bond formed by the side-chain hydroxyl of T195. They also indicate that the T195 hydroxyl and K198 amino side-chains in the M1-motif form different hydrogen bond partnerships in the open and closed conformations. These may contribute to the efficent formation of a compact Cdr1p structure that is recognised by the translocation machinery rather than degradation pathways. For example, the formation of a hydrogen bond between the side chain amino group of K198 of NBD1 and the conserved Y584 in ICL1 in the presence of ATP could provide a conformational check reflecting the successful integration of transmembrane loop 1+2 into the membrane. We believe that this model-based interpretation of the experimental evidence is reasonable as it is consistent with our previous study which identified suppressor mutations involving interactions between ICL and the Walker A motif in NBD1^22^. We note an important caveat. Cdr1p in its native commensal environment is synthesised and operates at 37 °C. Since lower temperature facilitates improved localization of Cdr1p-GFP to the PM, expression at 30 °C in *S. cerevisiae* might therefore significantly mask the impact of side-chain interactions on NBD structure in our experiments. This may explain why the M1-Ala2 mutant gives substantial, but incomplete, rescue of pump function *i.e.* it shows susceptibility to ANI, CYH and FLC but not to long-tailed triazoles that may have a different binding pockets in the membrane sector^5^.

Deletion of the M2-motif did not impact on protein folding or localization. The resultant protein appeared to be correctly transferrred to the PM but was non-functional i.e. its ATPase and efflux activities were severely reduced. Scanning alanine mutagenesis implied that the entire M2-motif, and not individual side-chains, was required for Cdr1p function. While replacement of the M2-motif with hexa-alanine gave correct localization as expected, it failed to confer function as Cdr1p-specific ATPase activity and the efflux of the transport substrates R6G and NR were severely reduced. Collectively, these results suggest the involvement of multiple side-chains in M2-motif function. Deletion of either the amino-terminal or the carboxyl-terminal half of the M2-motif resulted in a correctly translocated enzyme, but only the C-terminal deletion gave a functional enzyme. This implies that either the residues ^906^NCL are not essential for function or that residues such as ^909^SER can compensate for the loss of ^906^NCL e.g. a M2-motif comprising ^903^TLLSER is functional.

The orientation of the M2-motif may be critical as deletion of the conserved T903 in M2-Del903 resulted in non-functional protein variant that lacked ATPase activity and conferred drug suceptibility. Assuming that the helical structure of the M2-motif is retained but rotated by 100°, the replacement of T903 with L904 should not affect the main chain amide interactions between the M2-motif and the Walker A motif in the open conformation or with the α-phosphate of ATP in the closed conformation. It would, however, result in the loss of the T903 side-chain-mediated hydrogen bond with the G900 main chain carbonyl in the open conformation and with the Y866 phenolic hydroxyl in the closed conformation. In the open conformation the N906 side-chain-mediated hydrogen bond with the R911 side-chain would also be lost. In the closed conformation, the hydrophobic side-chain of L903 should make the environment near the ribose and α-phosphate of ATP more hydrophobic. Finally the N906 side-chain-mediated hydrogen bond with the R301 side-chain should be lost. R301 is an amino acid residue that is almost immediately amino terminal of the NBD1 signature sequence. The deletion of T903 is therefore predicted to deleteriously affect multiple aspects of ATP catalysis and enzyme function, including a substantial reduction in both Vmax and the affinity (1/Km) of the enzyme for ATP. The results shown in Table 2 are consistent with this interpretation. They are further supported by the mutant enzyme showing a binding affinity for R6G comparable to the WT enzyme (Table 2). Results obtained with the deletion of L905 in the M2-Del905 mutant are also consistent with this interpretation. The M2-Del905 mutant retained an environment in the active site that facilitated main chain and side-chain interaction of the M2-motif with the Walker A motif in the open conformation and with the α-phosphate of ATP and Y866 in the closed conformation. At the biochemical level this resulted in the predicted detection of a near normal ATP-binding and hydrolysis cycle. Despite the retention of ATP activity, the enzyme also had a significantly lower affinity for R6G. Whether this is a longer-distance consequence of a loss of hydrogen bonding between N906 and N911 in the open conformation and N906 and R301 in the closed conformation remains to be established. Mutagenesis studies needed to probe these model-based predictions further are beyond the scope of current study.

A combination of molecular moldeling, molecular genetic manipulation, phenotypic analysis and biochemical techniques has provided insight into how the M1- and M2-motifs in the ATP binding sites of Cdr1p may contribute to the physiology of this important fungal ABC transporter in an asymmetric manner. We conclude that these conserved motifs are structurally and functionally important. While the M1-motif in NBD1 is required for maturation of Cdr1p, the M2-motif in NBD2 is important for ATP catalysis. This report provides the first experimental exploration of the function of the M1- and M2-motifs despite their wider occurrence in ABC transporter subfamilies beyond the PDR family. The fact that they are essential for *S. cerevisiae* Pdr5p function supports the widespread importance of the motifs. An explanation of these motifs among a range of fungal PDR transporters will be needed to gain further insight into the significance of their conservation. Until direct experimental evidence can be obtained from high resolution X-ray crystal structures of Cdr1p in open and closed conformations, the present study provides a platform to explore how multiple features in these motifs determine conformations of Cdr1p that are suitable for transit to the cell surface and enable the enzyme to perform the complex catalytic cycle required for xenobiotic efflux.

## Methods

### Materials

Rhodamine 6G (R6G), ketoconazole (KTC), anisomycin (ANI), miconazole (MIC), cycloheximide (CYH), itraconazole (ITC), nile red (NR), adenosine triphosphate (ATP), oligomycin, phenylmethanesulfonyl fluoride (PMSF), tosyllysine chloromethyl ketone (TLCK), and tosyl phenylalanyl chloromethyl ketone (TPCK) were procured from Sigma Chemical Co. (St. Louis, MO). The protease inhibitors leupeptin, pepstatin A, and aprotinin were purchased from G-Biosciences (MO, USA). Fluconazole (FLC) was generously provided by Ranbaxy, India. Ascorbic acid (AA) was purchased from SRL (Mumbai, India). Oligonucleotides used in this study were purchasedfrom Sigma Genosys, India and are listed in Supplementary Table S2. Anti-GFP monoclonal antibody and anti-mouse secondary antibody were purchased from Santa Cruz Biotechnology Inc. (Texas, USA).

### Strains and media used

The yeast strains used in the study are listed in Supplementary Table S3. Plasmids were maintained in *E. coli* Dh5α cultured in Luria-Bertani medium (Difco, BD Biosciences, MD, USA) to which ampicillin was added (0.1 mg/ml). WT and *CDR1* mutant strains were cultured either in YPD broth or on YPD agar plates. Media were obtained either from Difco (Detroit, MI) or HiMedia (Mumbai, India). SD-ura^−^ dropout medium (0.67% yeast nitrogen base, 0.2% dropout mix and 2% glucose) containing 2.5% (w/v) agar was used for growth and selection of mutants after yeast integration.

### Site directed mutagenesis

Introduction of mutations at particular positions of *CDR1* was achieved by PCR amplification of the pPSCDR1-GFP plasmid containing the CDR1 gene with predesigned primers containing the mutation of interest. Mutagenesis was performed with a Quick Change site directed mutagenesis kit from Agilent technologies as per the manufacturer’s instructions. The introduction of mutations was confirmed by DNA sequencing. Positive plasmid clones were used for transformation of the *S. cerevisiae* strain AD1-8u^−^ using the lithium acetate transformation protocol after linearization with *Xba*I as described previously^20^.

### Immunodetection and Confocal microscopy

PM were prepared and used for immunodetection of Cdr1p in WT and mutant variants as described previously^20^. Immunodetection of GFP-tagged Cdr1p was performed using HRP-labeled anti-GFP antibody at a 1:5000 dilution. GFP-tagged proteins were imaged using an Olympus FluoView™ FV1000 laser confocal microscope (PA, USA) with 100X oil immersion objective lens.

### Drug susceptibility assays

Susceptibility of yeast to various drugs was evaluated either by broth microdilution or using agarose-based drug susceptibility assays. Agarose-based drug susceptibility assays were undertaken as described previously^20^. Briefly, 5-fold serial dilutions (5 μl), namely 1 (1:5), 2 (1:25), 3 (1:125) and 4 (1:625), of each strain were spotted on to YEPD plates in the absence (control) and presence of the following drugs: FLC (4 μg/ml); ITC (0.25 μg/ml); KTC (0.125 μg/ml); MCZ (0.25 μg/ml); ANI (2 μg/ml); CYH (0.062 μg/ml); R6G (3 μg/ml) and the growth of colonies was recorded following incubation of the plates for 48 h at 30 °C.

### Substrate transport assays

R6G efflux was determined essentially as described previously^20^. Briefly, log phase cells were washed and resuspended as a 2% suspension in PBS solution and incubated for 2 h in the presence of R6G at a 10 μM final concentration. After washing, the cells were resuspended in PBS with 2% glucose. After 40 min, a 1 ml portion of cells was centrifuged, and the absorbance of the supernatant was measured at 527 nm. NR was used at a final concentration of 7 μM, and its accumulation in WT and *CDR1* mutants was assessed by flow cytometry using a FACSort flow cytometer (Becton–Dickson Immunocytometry Systems, San Jose, CA). CellQuest software (Becton Dickinson Immunocytometry Systems, San Jose, CA) was used for data analysis^23^.

### Oligomycin sensitive ATPase assay

ATPase activity due to Cdr1p in WT and mutant variants was monitored by the oligomycin-sensitive release of inorganic phosphate as described previously^20^. PM protein (10 μg) from WT and each mutant strain was used to determine the Cdr1p ATPase activity in an *in vitro* assay. The reaction was started by adding 5 mM ATP to the reaction mixture and was terminated after 30 min of incubation at 30 °C by addition of 1 ml of stop solution containing 0.5% SDS.

### ATP agarose binding assay

The ability of Cdr1p to bind ATP was determined by using the method described by Ellinger *et al.*^21^. Briefly, 25 ml of a 1:1 slurry of C8-linked ATP-agarose resin (Sigma) equilibrated in resuspension buffer was added to 50 μg of PM and incubated at 4 °C on a rotator. After 2 h, the resin was pelleted by centrifugation (8200xg, 2 min, 4 °C) and washed three times with 250 μl of resuspension buffer. Bound proteins were eluted in SDS sample buffer by heating the resin to 65 °C for 20 min. The pellet samples were subjected to SDS-PAGE and analyzed by immunoblotting with anti-GFP monoclonal antibody.

### Time-Resolved Fluorescence Spectroscopy (TRFS)

The interaction of R6G with native Cdr1p was measured as described previously^5^. Briefly, fluorescence lifetime decays and anisotropy decay measurements were made in time-correlated single photon counting (TCSPC) mode using an FL920 instrument (Edinburgh Instruments, Livingston, UK). The R6G in the sample was excited at 470 nm using a picosecond (ps) pulsed diode laser (pulse width ~85 ps; instrument response function, IRF, ~110 ps). The fluorescence lifetime decays at an emission wavelength of 555 nm were collected at the magic angle (54°) and at a parallel and perpendicular polarisation relative to the vertical polarisation of excitation. From the decays measured at parallel and perpendicular polarisation, the anisotropy decay incorporating the value of the instrumental *G*-factor was calculated using equation 1.


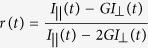


The interaction of R6G with native Cdr1p was measured by determining the time-resolved fluorescence and anisotropy decays of R6G, free in PBS and then bound to Cdr1p. The data were analyzed using IGOR-Pro software (Wave Metrics, USA). All fluorescence lifetime and anisotropy experiments were performed with purified PM from cells overexpressing Cdr1p (AD-CDR1) and with PM from host cells that did not express Cdr1p (AD1-8u^−^).

## Additional Information

**How to cite this article**: Rawal, M. K. *et al.* Newly identified motifs in *Candida albicans* Cdr1 protein nucleotide binding domains are pleiotropic drug resistance subfamily-specific and functionally asymmetric. *Sci. Rep.* **6**, 27132; doi: 10.1038/srep27132 (2016).

## Supplementary Material

Supplementary Information

## Figures and Tables

**Table 1:** New conserved ABC protein motifs.

PDR subfamily-specific motif T-[FL]-L-[DKN]-x-[AIL]			
Organism	Transporter	NBD1	NBD2
*Candida albicans*	Cdr1p	^195^TLLKTI^200^	^903^TLLNCL^908^
	Cdr2p	^193^TLLKTI^198^	^901^TLLNCL^906^
	Cdr3p	^186^TFLKTI^191^	^884^TLLNAL^890^
	Cdr4p	^203^TFLKTI^208^	^890^TLLNAL^895^
*Candida glabrata*	CgCdr1p	^190^TLLKSI^195^	^901^TLLDCL^906^
*Candida krusei*	ABC1	^212^TFLKTI^217^	^908^TLLDVL^913^
*Saccharomyces cerevisiae*	Pdr5p	^200^TLLKSI^205^	^913^TLLDCL^918^
	Pdr10p	^222^TLLKSI^227^	^967^TLLDCL^972^
	Pdr12p	^189^TFLKCL^194^	^886^TLLNVL^891^
	Pdr15p	^214^TLLKSI^219^	^931^TLLDCL^936^
*Aspergillus nidulans*	AtrB	^144^TLLKML^149^	^839^TLLDVL^844^
*Magnaporthe grisea*	ABC1	^243^TFLKTI^248^	^939^TLLDCL^944^
*Penicillium digitatum*	PMR1	^172^TFLKTI^177^	^871^TLLDVL^876^
MDR1 subfamily-specific motif T-x(3,4)-L-x(1,2)-R-x-Y-[DNS]-[GIPV]-x(2)-[GS]-x(4)-[DGNT]-[DGNT]			
*Homo sapiens*	P-gp	^435^TTVQLMQRLYDPTEGMVSVDG^455^	^1078^TVVQLLERFYDPLAGKVLLDG^1098^
MRP1 subfamily-specific motif L-x-D-x(0,2)-L-x(4)-[ADEGST]-x-[AGIV]-[AGNPST]			
*Homo sapiens*	MRP1-human	^790^LFDDPLSAVDAHVG^713^	^1363^LHDLRFKITIIP^1374^

Sequence motifs were identified in the NBDs of ABC transporter subfamilies^17^. The sequence motifs were described using a PROSITE-like syntax with amino acids represented by the standard IUPAC one-letter code. Repeated elements of the pattern are indicated by a numeric value following that element and gaps (‘x’) by a numerical range between parenthese.

**Table 2:** Kinetics of ATPase and drug transport activities of M2-Del903 and M2-Del905.

Transport and Kinetics Parameters		Cdr1p	M2-Del903	M2-Del905
ATP kinetics	ATPase activity (nmoles/mg/min)	78 ± 14	27.1 ± 4.4	55 ± 11
	K_m_ (mM)	2.1 ± 0.5	6.7 ± 0.8	2.9 ± 0.8
	V_max_ (nmoles/mg/min)	105 ± 15	45.3 ± 9.1	99.2 ± 8.9
Drug transport kinetics	R6G efflux (nmoles/10^8^cells)	4.3 ± 0.6	1.2 ± 0.5	1.2 ± 0.2
	R6G binding K_dR6G_ (μM)	9.0 ± 1.8	11.9 ± 1.9	25 ± 2.0
	R6G efflux rate (nmoles/10^8^cells/min)	4.3 ± 0.4	2.1 ± 0.3	2.6 ± 0.4

The values are the means ± standard deviation of three independent experiments. Km and Vmax values were calculated using Lineweaver-Burke plots. Differences greater than 2-fold compared with Cdr1p were considered significant (underlined).

**Table 3:** Contributions of free and bound R6G in the complexes obtained from anisotropy decay analysis of M2-Del903 and M2-Del905.

Sample	Fraction of *free*R6G ( 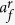 ) with time-constant ( 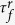 = 345 ps)	Fraction of *bound* R6G ( 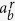 ) with time constant ( 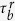 = 3.1 ns)	Normalised residual anisotropyof *bound* R6G (*b*)	Total *bound* contribution [ 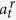 (total) = 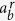 + *b*]
R6G	1	0	0	0
AD1-8u^−^	0.66	0.27	0.07	0.34
Cdr1p-GFP	0.51	0.34	0.15	0.49
M2-Del903	0.48	0.33	0.19	0.52
M2-Del905	0.61	0.32	0.07	0.39

All anisotropy decays are fitted with equation: 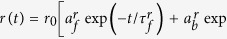

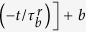
.
