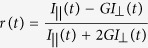# Erratum: Newly identified motifs in *Candida albicans* Cdr1 protein nucleotide binding domains are pleiotropic drug resistance subfamily-specific and functionally asymmetric

**DOI:** 10.1038/srep30221

**Published:** 2016-07-25

**Authors:** Manpreet Kaur Rawal, Atanu Banerjee, Abdul Haseeb Shah, Mohammad Firoz Khan, Sobhan Sen, Ajay Kumar Saxena, Brian C. Monk, Richard D. Cannon, Rakesh Bhatnagar, Alok Kumar Mondal, Rajendra Prasad

Scientific Reports 6: Article number: 2713210.1038/srep27132; published online: 06 02 2016; updated: 07 25 2016

This Article contains a typographical error in Equation 1.


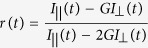


should read: