# Supplementary material for: Newly identified motifs in Candida albicans Cdr1 protein nucleotide binding domains are pleiotropic drug resistance subfamily-specific and functionally asymmetric
Source: Sci Rep. 2016 Jun 2;6:27132. doi: 10.1038/srep27132 (PMC4890005; doi:10.1038/srep27132)
Supplement: Supplementary Information [file srep27132-s1.pdf]

Newly identified motifs in *Candida albicans* Cdr1 protein nucleotide binding domains are pleiotropic drug resistance subfamily-specific and functionally asymmetric

Manpreet Kaur Rawal[1], Atanu Banerjee[1], Abdul Haseeb Shah[1†], Mohammad Firoz Khan[2], Sobhan Sen[2], Ajay Kumar Saxena[1], Brian C. Monk[3], Richard D. Cannon[3], Rakesh Bhatnagar[4], Alok Kumar Mondal[1]and Rajendra Prasad[1*‡]

[1]School of Life Sciences, [2]School of Physical Sciences, [3]The Sir John Walsh Research Institute, University of Otago, Dunedin 9054, New Zealand, [4]School of Biotechnology, Jawaharlal Nehru University, New Delhi, 110067, India

[*]To whom correspondence should be addressed: Rajendra Prasad, School of Life Sciences, Jawaharlal Nehru University, New Delhi 110067, India, Tel.: 91-11-26704509. [‡]Present address: Amity Institute of Integrative Sciences and Health, Amity University Haryana, Gurgaon 122413, Haryana, India

Email:[*‡]rp47jnu@gmail.com rprasad@ggn.amity.edu

[†]Present address: Department of Bioresources, University of Kashmir, Srinagar-190006, India

Supplementary Information

Supplementary Figure S1. (A) Confocal microscopy images showing expression and membrane localization of the WT Cdr1p and deletant mutant variants M1-Del1, M1-Del2 and M2-Del1 (upper panel) and length restored mutant variants M1-Ala1, M1-Ala2 and M2-Ala1 (lower panel). (B) The drug resistance profiles of yeast strains overexpressing WT Cdr1p and mutant variants M1-Ala1, M1-Ala2 and M2-Ala1 were determined using agarose-based drug susceptibility assays as described in Methods section.

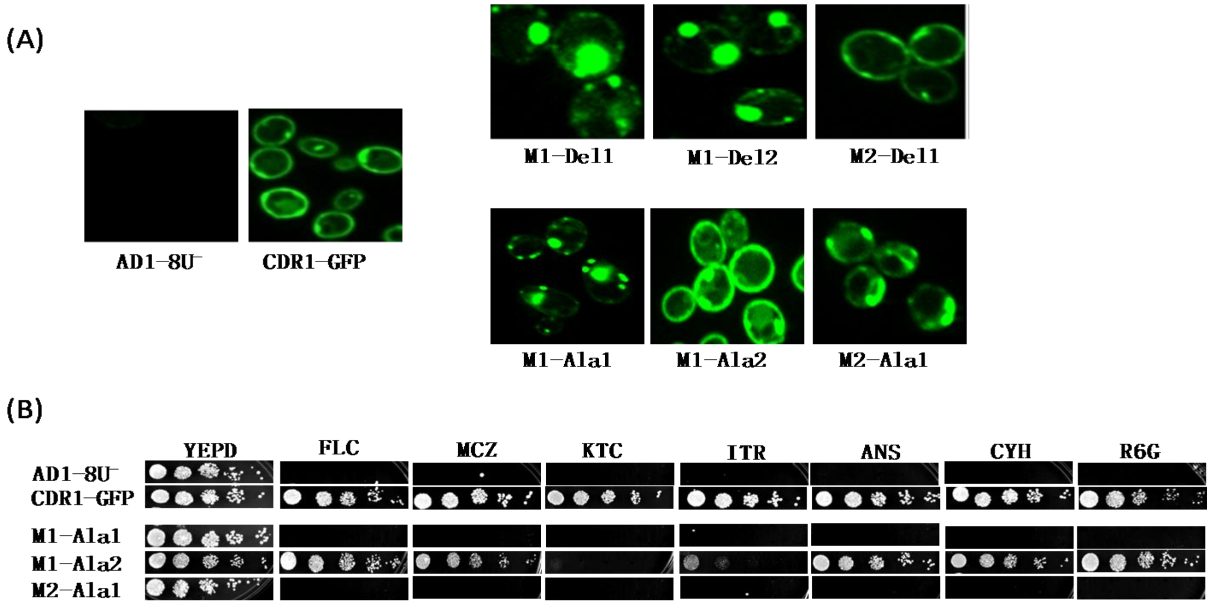

Supplemental Figure S2: Titration of Cdr1p with constant concentration of R6G (3 µM). Left Panel: Raw anisotropy decays of R6G at different concentrations of Cdr1p. Right panel: Extracted (total) bound and free fractions of R6G to the protein. Data shows the increase in *bound* fraction and the concomitant decrease in *free* fraction of R6G with increase in Cdr1p concentration which directly validates the binding of R6G to the protein.

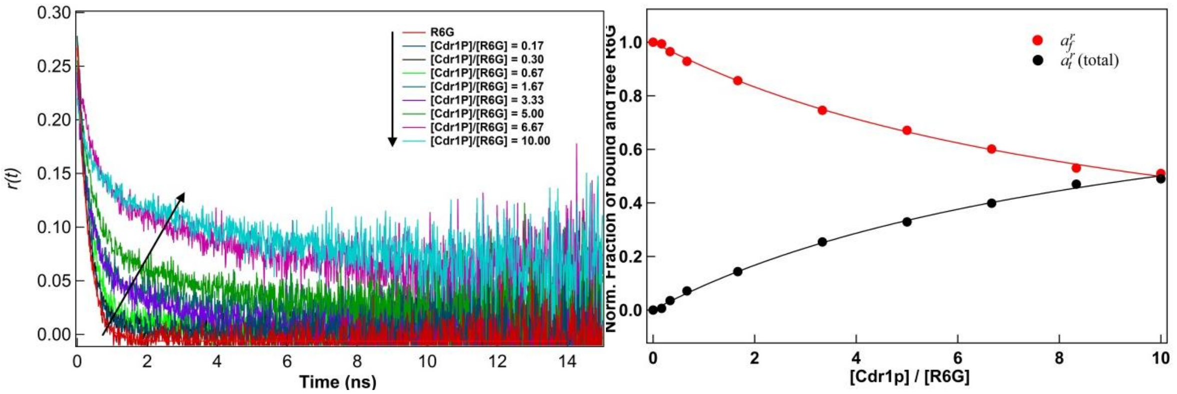

Supplementary Table S1. MIC$_{80}$ values (μg/ml) obtained for M1-motif and M2-motif variants. The MIC values showing 4-fold or greater differences from values for wilt type *CDR1* are shown in red.

Strains	FLC	ITC	KTC	MCZ	ANI	CYH	R6G
AD1-8U$^-$	0.5	0.015	0.015	0.015	0.25	0.012	0.37
CDR1	64	4	2	4	32	1	48
M1-T195A	64	4	2	4	32	1	24
M1-L196A	64	4	2	4	32	1	48
M1-L197A	64	4	2	4	32	1	48
M1-K198A	64	4	1	2	8	0.5	48
M1-T199A	32	4	2	4	32	1	24
M2-I200A	64	4	2	4	32	1	48
M2-T903A	64	4	2	2	32	1	48
M2-L904A	64	4	2	4	32	1	48
M2-L905A	64	4	2	4	32	1	48
M2-N906A	64	4	2	4	32	1	48
M2-C907A	32	4	2	2	32	1	48
M2-L908A	64	4	2	4	32	1	48
M1-Del	0.5	0.015	0.015	0.015	0.25	0.012	0.37
M2-Del	0.5	0.015	0.015	0.015	0.25	0.012	0.37
M1-Ala	64	4	0.015	1	4	1	48
M2-Ala	0.5	0.015	0.015	0.015	0.25	0.012	0.74
M1-Del1	0.5	0.015	0.015	0.015	0.25	0.012	0.37
M1-Del2	0.5	0.015	0.015	0.015	0.25	0.012	0.37
M2-Del1	0.5	0.015	0.015	0.015	0.25	0.012	0.74
M2-Del2	64	4	2	4	32	1	48
M2-Del903	0.5	0.015	0.015	0.015	0.25	0.012	0.37
M2-Del905	0.5	0.015	0.015	0.015	0.25	0.012	0.74

Supplementary Table S2. Oligonucleotides used for site-directed mutagenesis.

Oligo Name	5'<-----Sequence----->3'
M1-T195A/F	GGTGCTGGTTGTTCCGCATTGTTAAAGACC
M1-L196A/F	GGTTGTTCCACAGCGTTAAAGACCATTGC
M1-L197A/F	GGTTGTTCCACATTGGCAAAGACCATTGCTG
M1-K198A/F	GTTCCACATTGTTAGCGACCATTGCTGTTAAC
M1-T199A/F	CCACATTGTTAAAGGCCATTGCTGTTAACAC
M2-I200A/F	CATTGTTAAAGACCGCTGCTGTTAACACTTATGG
M2-T903A/F	GGTGCTGGTAAGACTGCATTGTTGAATTG
M2-L904A/F	GGTGCTGGTAAGACTACAGCGTTGAATTGTTTATCTTG
M2-L905A/F	GCTGGTAAGACTACATTGGCGAATTGTTTATCTGAAAGAG
M2-N906A/F	GCTGGTAAGACTACATTGTTGGCTTGTTTATCTGAAAGAG
M2-C907A/F	GACTACATTGTTGAATGCTTTATCTGAAAGAGTCAC
M2-L908A/F	GACTACATTGTTGAATTGTGCATCTGAAAGAGTCAC
M1-Del/F	GGAGACCCGGTGCTGGTTGTTCCGCTGTTAACACTTATGGTTTCC
M2-Del/F	GGGAGCATCTGGTGCTGGTAAGACTTCTGAAAGAGTCACTACTGG
M1-Ala/F	GCTGGTTGTTCCGCAGCGGCAGCGGCCGCTGCTGTTAACAC
M2-Ala/F	GGTGCTGGTAAGACTGCAGCGGCGGCTGCTGCATCTGAAAGAGTCAC
M1-Del1/F	CCGGTGCTGGTTGTTCCAAGACCATTGCTGTTAAC
M1-Del2/F	GGTTGTTCCACATTGTTAGCTGTTAACACTTATGG
M2-Del1/F	CTGGTGCTGGTAAGACTAATTGTTTATCTGAAAG
M2-Del2/F	GTAAGACTACATTGTTGTCTGAAAGAGTCACTACTG
M2-Del903/F	CTGGTGCTGGTAAGACTTTGTTGAATTGTTTATC
M2-Del905/F	GCTGGTAAGACTACATTGAATTGTTTATCTGAAAG
M1-Ala1/F	CTGGTTGTTCCGCAGCGGCAAAGACCATTGCTG
M1-Ala2/F	CCACATTGTTAGCGGCCGCTGCTGTTAACAC
M2-Ala1/F	CTGGTAAGACTGCAGCGGCGAATTGTTTATC

Supplementary Table S3. Yeast strains used in the study.

Strains	***Genotype or description***	***Source***
AD1-8U$^-$	(*Mata,pdr1-3,ura3 his1, Δyor1::hisG, Δsnq2::hisG, Δpdr5::hisG, Δpdr10::hisG, Δpdr11::hisG, Δycf1::hisG, Δpdr3::hisG, Δpdr15::hisG*)	Nakamura, 2001
CDR1	AD1-8u$^-$ cells harboring*CDR1* ORF integrated at *PDR5* locus	Nakamura, 2001
M1-T195A	AD1-8u- cells harboring CDR1–GFP ORF with a T195A mutation and integrated at *PDR5* locus	This study
M1-L196A	AD1-8u- cells harboring CDR1–GFP ORF with a L196A mutation and integrated at *PDR5* locus	This study
M1-L197A	AD1-8u- cells harboring CDR1–GFP ORF with a L197A mutation and integrated at *PDR5* locus	This study
M1-K198A	AD1-8u- cells harboring CDR1–GFP ORF with a K198A mutation and integrated at *PDR5* locus	This study
M1-T199A	AD1-8u- cells harboring CDR1–GFP ORF with a T199A mutation and integrated at *PDR5* locus	This study
M2-I200A	AD1-8u- cells harboring CDR1–GFP ORF with a I200A mutation and integrated at *PDR5* locus	This study
M2-T903A	AD1-8u- cells harboring CDR1–GFP ORF with a T903A mutation and integrated at *PDR5* locus	This study
M2-L904A	AD1-8u- cells harboring CDR1–GFP ORF with a L904A mutation and integrated at *PDR5* locus	This study
M2-L905A	AD1-8u- cells harboring CDR1–GFP ORF with a L905A mutation and integrated at *PDR5* locus	This study
M2-N906A	AD1-8u- cells harboring CDR1–GFP ORF with a N906A mutation and integrated at *PDR5* locus	This study
M2-C907A	AD1-8u- cells harboring CDR1–GFP ORF with a C907A mutation and integrated at *PDR5* locus	This study
M2-L908A	AD1-8u- cells harboring CDR1–GFP ORF with a L908A mutation and integrated at *PDR5* locus	This study
M1-Del	AD1-8u- cells harboring CDR1–GFP ORF with deletion of residues 195-200 in *CDR1* ORF and integrated at *PDR5* locus	This study
M2-Del	AD1-8u- cells harboring CDR1–GFP ORF with deletion of residues 903–908 in *CDR1* ORF and integrated at *PDR5* locus	This study
M1-Ala	AD1-8u- cells harboring CDR1–GFP ORF with alanine substitution of residues 195-200 in *CDR1* ORF and integrated at *PDR5* locus	This study
M2-Ala	AD1-8u- cells harboring CDR1–GFP ORF with alanine substitution of residues 903–908 in *CDR1* ORF and integrated at *PDR5* locus	This study
M1-Del1	AD1-8u- cells harboring CDR1–GFP ORF with deletion of residues 195-197 in *CDR1* ORF and integrated at *PDR5* locus	This study
M1-Del2	AD1-8u- cells harboring CDR1–GFP ORF with deletion of residues 198-200 in *CDR1* ORF and integrated at *PDR5* locus	This study

M2-Del1	AD1-8u- cells harboring CDR1–GFP ORF with deletion of residues 903-905 in *CDR1* ORF and integrated at *PDR5* locus	This study
M2-Del2	AD1-8u- cells harboring CDR1–GFP ORF with deletion of residues 906-908 in *CDR1* ORF and integrated at *PDR5* locus	This study
M2-Del903	AD1-8u- cells harboring CDR1–GFP ORF with deletion of residue 903 in *CDR1* ORF and integrated at *PDR5* locus	This study
M2-Del904	AD1-8u- cells harboring CDR1–GFP ORF with deletion of residue 904 in *CDR1* ORF and integrated at *PDR5* locus	This study
M2-Del905	AD1-8u- cells harboring CDR1–GFP ORF with deletion of residue 905 in *CDR1* ORF and integrated at *PDR5* locus	This study
M1-Ala1	AD1-8u- cells harboring CDR1–GFP ORF with alanine substitution of residues 195-197 in *CDR1* ORF and integrated at *PDR5* locus	This study
M1-Ala2	AD1-8u- cells harboring CDR1–GFP ORF with alanine substitution of residues 198-200 in *CDR1* ORF and integrated at *PDR5* locus	This study
M2-Ala1	AD1-8u- cells harboring CDR1–GFP ORF with alanine substitution of residues 903-905 in *CDR1* ORF and integrated at *PDR5* locus	This study

Supplementary Alignment 1. PRALINE™ aligned multiple sequence alignment (MSA) of fungal PDR transporters used in the study.

```
>P43071 caCDr1
--------------------------------------------------
--------------------------------------------------
--------------------------------------------------
--------------------------------------------------
--------------------------------------------------
--------------------------------------------------
--------------------------------------------------
--------------------------------------------------
------------------------------MSDSKMSSQDESKLEKAISQ
DSSSENHSINEYHGFDAHTSENIQNLARTFTHDSFK--DDSSA--GLLKY
LTHMSEVPGVNPYEHEEINNDQLNPDSENFNAKFWVKNLR-KLFESDPEY
YKPSKLGIGYRNLRAYGVANDSDYQPTVTNALWKLATEGFRHFQKDDD--
----SRY----FDILKSMDAIMRPGELTVVLGRPGAGCSTLLKTIAVNTY
GFHIGKE--SQI------TYDGLSPH---DIERHYRGDVI--YSAETDVH
FPHLSVGDTLEFAARLRTPQNRGE-----GIDRETYAKHMASVYMATYGL
SHTRNTNVGNDFVRGVSGGERKRVSIAEASLSGANIQCWDNATRGLDSAT
ALEFIRALKTSAVILDTTPLIAIYQCSQDAYDLFDKVVVLYEGYQIFFGK
ATKAKEYFEK-MGWKC-PQRQTTADFLTSLTNPAEREP---LPG-YED--
KVPRTAQEFETYWKNSPEYAELTKEIDEYFVEC-----------------
E---RSNTRETYRESHVAKQSNNTRPASP--YTVSFFMQVRYGVARNFLR
MKGDPSIPIFSVFGQLVMGLILSSVFYNL----SQTTGSFYYRGAAMFFA
VLFNAFSSLL-EIMSLFEAR-PIVEKHKKYALYRPSADALA-SIISELPV
KLAMSMSFNFVFYFMVNFRRNPGRFFFYWLMCIWCTFVMSHLFRSIGAVS
TSISGAMTPATVLLLAMVIYTGFVIPTPSML---GWSRWINYINPVGYVF
ESLMVNEFHGREFQC--AQYVP----SGP-G-YENISRSNQVCTAV-GSV
PGNEMVSGTN-YLAGAYQYYNSHKWRNLGITIGFAVFFLAIYIALTEFNK
GAMQKGEIVLFLKGSLKKHKRKTAASNKGDIEAGPVAGKLDYQDEAEAVN
NEKFTE-------------------------------KGST---GSVDFP
```

ENREIFFWRDLTY---QVKIKK-EDRVILDHVDGWVKPGQITALMGASGA
GKTTLLNCLSERVTTGII----TDGERLVNGHALDS--SFQRSIGYVQQQ
DVH-LPTSTVREALQFSAYLRQSNKISKKEKDDYVDYVIDLLEMTDYADA
LVGV--AGEGLNVEQRKRLTIGVELVAKPKLLLFLDEPTSGLDSQTAWSI
CKLMRKL-ADHGQAILCTIHQPSALIMAEFDRLLFLQKGGRTAYFGELGE
NCQTMINYFEKYGADPCPKEANPAEWMLQVVGAAPG--------------
-------SH-AK-QDYFEVWRNSSEYQAVREEINRMEAELSKLP------
---RDNDPEALLKYAAPLWKQYLLVSWRTIVQDWRSPGYIYSKIFLVVSA
ALFNGF-SFFK--AKNNMQGLQNQMFSVFMFFIPFNTLVQQMLPYFVKQR
DVYEVREAPSRTFSWFAFIAGQITSEIPYQVAVGTIAFFCWYYPLGLYNN
ATPTDSVNPRGVLMWMLVTAFYVYTATMGQLCMSFSELADNAANLATLLF
TM-CLNFCGVLAGPDVLPGFW-IFMYRCNPFTYLVQAMLSTGLANTFVKC
AEREYVSVKP--PNG-ESCSTYLDPYIK-----------FAGGYFET-RN
----DGSCAFCQMSS--TNTF--LKSVNSL-YSERWRNFGIFIAFIAINI
ILTVIFYWLARVPKGNREKKNKK---------------------------

>P78595 CaCDr2

---------------------------------MSTANTSLSQQLDENPW
VDASDNSSVQEYQGFDATASHNIQDLARKLTHGSTNGDHHSAN--DLARY
LSHMSDIPGVSPFN-GNISHEQLDPDSENFNAKYWVKNLK-KLFESDSDY
YKPSKLGVAYRNLRAYGIANDSDYQPTVTNALWKFTTEAINKLKKPDD--
----SKY----FDILKSMDAIMRPGELTVVLGRPGAGCSTLLKTIAVNTY
GFHIGKE--SQI------TYDGLSPH---DIERHYRGDVI--YSAETDVH
FPHLSVGDTLEFAARLRTPQNRGE-----GIDRETYAKHMASVYMATYGL
SHTRNTNVGNDFVRGVSGGERKRVSIAEASLSGANIQCWDNATRGLDSAT
ALEFIRALKTSATILDTTPLIAIYQCSQDAYELFDNVVVLYEGYQIFFGK
ASKAKEYFEN-MGWKC-PQRQTTADFLTSLTNPAEREP---LPG-YED--
KVPRTAQEFETFWKNSPEYAELTKEIDEYFVEC-----------------
E---RSNTGETYRESHVGKQSNNTRPSSP--YTVSFFMQVRYVIARNFLR
MKGDPSIPLISILSQLVMGLILASVFFNL----RKSTDTFYFRGGALFFS
VLFNAFSSLL-EILSLYEAR-PIVEKHRKYALYRPSADALA-SIISELPV
KLLMTMSFNIVYYFMVNLRRTAGNFFFYWLMCASCTLVMSHMFRSIGAVT
TTIATAMSLSTVFLLAMIIYAGFVLPIPYIL---GWSRWIRYINPVTYIF
ESLMVNEFHGREFEC--GQYIP----SGP-G-FENLPVENKVCTTV-GST
PGSTVVQGTE-YIKLAYQFYSSHKWRNFGITVAFAVFFLGVYVALTEFNK
GASQKGEIVLFLKGSLKKHKRKTAASNKGDIEAGPVAGKLDYQDEAEAVN
NEKFTE-------------------------------KGST---GSVDFP
ENREIFFWRDLTY---QVKIKK-EDRVILDHVDGWVKPGQITALMGASGA
GKTTLLNCLSERVTTGII----TDGERLVNGHALDS--SFQRSIGYVQQQ
DVH-LETTTVREALQFSAYLRQSNKISKKEKDDYVDYVIDLLEMTDYADA
LVGV--AGEGLNVEQRKRLTIGVELVAKPKLLLFLDEPTSGLDSQTAWSI
CKLMRKL-ADHGQAILCTIHQPSALIMAEFDKLLFLQKGGRTAYFGELGE
NCQTMINYFEKYGADPCPKEANPAEWMLQVVGAAPG--------------

-------SH-AK-QDYFEVWRNSSEYQAVREEINRMEAELSKLP------
---RDNDPEALLKYAAPLWKQYLLVSWRTIVQDWRSPGYIYSKLILVISS
SLFIGF-SFFK--SKNNLQGLQSQMLAVFMFFVPFTTFIDQMLPYFVKHR
AVYEVREAPSRTFSWFAFIAGQITSEIPFQIVVGTISYFCWYYPVGLYAN
AEPTDSVNSRGVLMWMLLTAFYVYTSTMGQLAISLNELIDNAANLATTLF
TL-CLMFCGVLAGPNVIPGFW-IFMYRCNPFTYLIQAILSTGLANAKVTC
APRELVTLKP--PMG-ETCSSFIGPYTE-----------AAGGYFST-NS
----DGTCSVCRIDS--TNQF--LESINAL-FSQRWRNFGIFVAFIGINI
ILTIFFYWLARVPKGNREKKMKK---------------------------

>C5M884CtCDr1

--------------------------------MSDSKTSSQREWDQLEDNR
DSSSENHSINEYHGFDAHATEDIQNLARTLSHNSFK--DDSSA--GLMKY
LSHMSEVPGVSPFA-EEEINEQLNPDSDKFNAKFWVKNMR-KLVDSDPDY
YKPSKLGIAYRDLRAYGVANDSDYQPTVTNGLWKAIVEGIRFFQKEDE--
----SRC----FNILKPMDAIMKPGELTVVLGRPGAGCSTLLKTIAAQTY
GFHIGKE--SKI------TYDGLTQD---DIKKHYHGDVI--YSAETDIH
FPHLTVGDTLEFAARLRTPQNRGE-----GIDRETYAKHMASVYMATYGL
SHTRNTSVGNDFVRGVSGGERKRVSIAEASLSGANIQCWDNATRGLDAAT
ALEFIRALKTSAAILESTPLIAIYQCSQDAYDLFDNVVVLYEGYQIFFGK
ASKAKEFFLK-MGYKC-PQRQTTADYLTSLTNPAEREP---LPG-YED--
KVPRTPQEFEAYWKNSPEYAELIKDIDNYFVEC-----------------
E---KLNTKEIYHDSHVARQSNHIRPGSP--YTVSFYMQVRYGVARNFLR
MKGDPSIPIFSVFGQCVMGLILSSVFYNL----PQTTGSFYYRGASMFFA
VLFNAFASLL-EIMSLFEAR-PIVEKHKKYALYRPSADALA-SIISELPV
KLVMSLAFNLIFYFMVNFRRNAGRFFFYWLMCGWCTLVMSHLFRSIGAVS
TSLAGAMTPATVLLLAMIIYTGFVIPTPNML---GWSRWINYINPVGYVF
ESLMVNEFHDREFEC--STYIP----SGG-A-YESIPRENRACSAV-GST
PGSSIVNGTD-YLAQAYRYYNSHKWRNLGITIAFAVFFLGIYIFLTEFNK
GAMQKGEIVLFLRGSLKKRRKA-AADKSKDIETGNVVEKVNFQDVAEASN
SERMSE--------------------------------KGSM---GSDEIP
SNREIFFWKNLTY---QVKIKK-EDRVILDHVDGWVKPGQITALMGASGA
GKTTLLNCLSERVTTGVI----TDGERMVNGHALDS--SFQRSIGYVQQQ
DIH-LETSTVREALRFSAYLRQSSKISKKEKDEYVDYVIDLLEMTDYADA
LVGV--AGEGLNVEQRKRLTIGVELVAKPKLLLFLDEPTSGLDSQTAWSI
CKLMRKL-ADHGQAILCTIHQPSALIMAEFDRLLFLQKGGRTAYFGDLGR
NCQTMIDYFEKYGADPCPKEANPAEWMLEVVGAAPG--------------
-------SH-AK-QDYFEVWRNSDEYRAVHDEITRMETELVKLP------
---RDEDPEAKFKYAAPIWKQYLLVTWRTIVQDWRSPGYIYSKLFLAISS
ALFNGF-SFFK--ATNSLQGLQNQMFAIFMYFIPFNTLVQQMLPVFVKQR
DIYEVREAPSRTFSWFAFITAQISSEIPYMTVVGTISFFCWYYPVGLYRN
AEPTDAVDQRGVLMWMFLTGFFVYTSTMGQLCMSFNELADNAANLATLLF
TM-CLNFCGILATKDALPGFW-IFMYRCNPFTYLVQGLLSTGLANTEVTC

SSYEYVTVQP--PSG-QTCDNFLGPYAQ-----------AAGGYFLD-NS
----DGSCSFCQMRS--TNSF--LTSVNAI-YSERWRNFGIFVAFIAINI
CLTVFFYWWARVPKGTREKKHKK---------------------------

>A3LZU3

-----------------------------MSDASIGKESQEQKKNIPSNS
STVSDTSSINEYQGFNRSTAADVQELARTITHESYTFENDQASSIGLIKY
LSHMSQVPGVNPYV-EEETNPELDPNSESFNAKFWVKNLR-KLYDSDPEY
YQPSKLGIAYRDLRAYGVAADSDYQPTVTNAIWKFAVETSRTFQKEDP--
----SRY----FDILKPMDAIMKPGEVTVVLGRPGSGCSTLLKTISAHTY
GFHVGEE--SRI------TYDGLSPQ---DIVDNHRGDVV--YSAETDVH
FPHLTVGDTLEFAARLRTPQNRGL-----GVDRETYAKHMASVYMATYGL
SHTRNTNVGNDFVRGVSGGERKRVSIAEVSLSGANLQCWDNATRGLDAAT
ALEFIRALKTSATILEATPLIAIYQCSQDAYDLFDNVVLLYEGYQIFFGK
AEDAKTFFVN-MGYEC-PQRQTTADYLTSLTNPAERIV---IPG-YEN--
SVPRTAKEFEAYWKSSPEYKSLVEEIEQHFQDV-----------------
E---TNNVKQSYLDSHVAKQSKHLSPKSP--YTVSFFMQVRYIMGRNFLR
LKGDPSIAIFSVFGQGVMGLILSSVFYNL----SQTTESFYYRGAAMFFA
VLFNAFASLL-EIMSLFEAR-PIVEKHKKYALYRPSADALA-GIITELPT
KLCMSVSFNFVFYFMVNFRRTPGNFFFYWLMAGWCTLVMSHLFRSLGAVS
TSLAGAMTPATVLLLAMVIYTGFVIPTPKML---GWSRWINYINPVGYVF
ESLMVNEFHERDFEC--ATFVP----TGP-N-YVNVPEDARVCNTV-GAR
PGSNIVSGTD-YLALSYQYYHSHKWRNLGITIAFAVFFLFVYIALTEFNK
GAMQKGEITLFLRGSLKKHKKEAKKGKANVADIET-ADTNEKIGFSDELD
ATKNKS-------------------------------SSNS---GDNSLP
SSKDIFHWKDLTY---QVKIKS-EERVILNHCDGWVKPGQLTALMGSSGA
GKTTLLNCLSERVTTGVI----TDGVRMVNGHSLDS--SFQRSIGYVQQQ
DLH-LPTSTVREALRFSAYLRQPNSVSKKEKEEYVEYIIDLLEMYDYADA
LVGV--AGEGLNVEQRKRLTIGVELVAKPKLLLFLDEPTSGLDSQTAWSI
CKLMRKL-ADHGQAILCTIHQPSALLLKEFDRLLFLQSGGQTVYFGDLGE
NCSTLIEYFEKYGSHPCPKEANPAEWMLEVVGAAPG--------------
-------SH-AN-QDYHEVWKNSTEYKDVHEELAKMERELVLLP------
---KDDSPDSHLKYAAPIWKQYLIVSARVIQQNWRSPGYIYSKLFLVLSS
ALFNGF-SFFK--ADRSMQGLQNQMFAIFMFFIPFNTLVQQMLPYFVRQR
DVYEVREAPSRTFSWFAFITGQITAEIPYHVAVGTLSFFCWYYPVGLYQN
AVPTDSVNSRGVLMWLLLTSFFVYSGTMGQLCMAFNELADNAANLATLLF
TM-CLNFCGVLAGPDVLPGFW-IFMYRANPFTYLIQAILSTGLANTSVVC
SPNELVPLNP--PNG-QTCLEYMQPYID-----------ATGGYLVDENA
----TSGCQFCQMAK--TNDF--LKSVNAL-YSERWRNFGIFIAFIAINI
ILTVVFYYWARVPKGTRQKKSSK---------------------------

>B5RUI0

----------------------------MSTGSLSSAGRETKDINSQYEL
TDSSENNEIHEYEGFDNTATGDIRELARTVSHISREQTNKTSDSEDIVRY
LSHFSSIPGVEPY---TEAEDTLNPDSDSFDARLWVKNLR-KLHDSDVEY
FKPSSLGVAYRNLRACGVAADSDYQPTVLNGITKYLTDGFRYLQKDDP--
----SRY----FDILKSMDGIMRPGEVTVVLGRPGSGCSTLLKTIASHTY
GFKIGEE--SKI------SYDGLTPK---DIENQFRGDVV--YSAETDTH
FPHLTVGDTLEFAAKMRTPQNR-G-----NVDRETYAKHMASVYMATYGL
SHTRNTNVGDDFVRGVSGGERKRVSIAEVSLCGSNIQCWDNATRGLDSAT
ALEFIRALKTSATILDATPLIAIYQCSQDAYDLFDNVVVLYEGHQIFFGK
ADEAKEYFIN-MGWEC-PQRQTTADFLTSLTNPAERVP---RPG-FEN--
SVPYTPKEFETHWKNSPQYKKLVEDVEEYFQKT-----------------
D---SGNHGEEYHKAHVARQSNHISPKSS--FTVSFFMQTRYIMGRNILR
TKRNPSVAIQSIAGQAFIGITLGSMFYNL----SATTETLYYRCATLFGA
VLFNAFSSIL-EIMSLFEAR-PIIEKHKQYALYRPSADALA-GIITELPT
KLASSIAFNFFIYFLSNLRRDAGRFFFFWLMCCMCTLVMSHLFRSLGAIS
TSFAGAMTPATVLLLAMVIFAGFVLPTPSML---GWSRWINYLNPIAYVF
EALMANEYTDRDFEC--SQFVP----SGP-G-YEDRNSVHRICAAT-GSK
AGSDVLHGDD-YLSVSYEYYNFHKWRNFGITVGFIIFFLFVYITLTEFNK
GSMQKGEVALFLKSSLTDQKKKSGKSETTSKDIEN-SAIPDEK--ISQKD
QLEANK-------------------------------ETET---AEKALP
SSNDIFHWRDLTY---QVKIKS-EDRVILNHVDGWVKPGQLTALMGSSGA
GKTTLLNCLSERVTTGVI----SDGVRMVNGHSLDS--SFQRSIGYVQQQ
DLH-LPTSTVREALRFSAQLRQPNSVTTKEKNDYVEYIIDLLDMYPYADA
LVGV--AGEGLNVEQRKRLTIGVELVAKPKLLLFLDEPTSGLDSQTAWSI
CKLMRKL-ADHGQAILCTIHQPSALLLQEFDRLLFLQKGGKTVYFGDLGE
NCQTLINYFEKYGAHHCPEEANPAEWMLQVVGAAPG--------------
-------SH-AN-QDYHEVWRSSSEYQGTQAELDNMERELVNLP------
---VDESPEAKKSYAAPIWKQYLIVTKRVFQQNWRSPTYIYSKLFLVVSS
ALFNGF-SFFK--ADRSMQGLQNQMFAMFMFLIPFNTLVQQMLPYFVKQR
DVYEVREAPSKTFSWFAFVAAQITSEVPYQIFCGTIAFLCWFYPVGFYQN
AVPTNSVDQRAVLMWMYICSFYVYTSTMGQLCMSFNELADNAANLATLLF
TM-CLNFCGVLAGPDVLPGFW-IFMYRCSPFTYFIQGMLSTGLANTNAEC
SKAEFLHFKP--NEG-QSCGEYMSDYIK-----------QAGGYLVDEKA
----SSECQFCPMAS--TNDF--LASVNSF-YDERWRNWGIFICFIAINI
ILTIFFYWLARVPKGNREKKKK----------------------------

>C4Y3K0

----------------------------------MASTPEQPSVEKDYSD
AVSSENASVNEYRGFQGH-EGEIRQLARTLTQNSVGSDKSKSSE-GLLRY
LSHMSQVPGVPPF--EDGVDDTLNPESENFNAKFWVKNMR-KLYQSDPDY
YKPSKLGLAYRDLRAYGVATDSDYQPTVTDALWKMAVEGIRSLRKEDE--
----SRY----FDILKPMDGYFKPGEVTVVLGRPGSGCSTLLKTIACNTY
GFHIGKE--SKI------SYDGFTPD---EIAKHHRGDVV--YSAETDMH
FPHLSVGHTLEFAARLRTPQNR-G-----NVSREEYAKHMASVYMATYGL
SHTRNTKVGNDFVRGVSGGERKRVSIAEASLSGANIQCWDNATRGLDAAT
ALEFIRALKTAAAILDATPLIAIYQCSQDAYDLFDNVIVLYEGYQIYFGK
AASAKAFFEN-MGYEC-PQRQTTADYLTSLTNPAERIV---RPG-YEN--
KVPRTAKEFSDYWRASQEYKDLIAGIDNYMAEM-----------------
E---KGESKALYKESHNAKQSKNVHPGSP--FTVSFGMQVKYIVQRNFLR
MKGDPSIPLFSVVGQGIMGLILSSVFYNL----QRVTDSFYYRGASMFFA
VLFNAFASLL-EIMTLFEAR-PIVEKHKKFALYRPSADAMA-SIVSELPV
KLAMSTFFNFTFYFMVHFRRNPGRFFFYWLACGFCTLCMSHMFRSLGAVS
TSLAGAMTPATVILLAMVIFTGFVIPIPNML---GWCRWIQYINPVSYVF
ESLMVNEFAGVEYEC--SQYIP----SGP-G-YPQVATENNICNVV-GAV
RGRATVSGTA-FLAKSYDYHNSHKWRNIGIVIAYIVVFLGVYVSLTEFNK
GAMQKGEIVIYLKGSLKKMKKKTEANKATSDDLEN-----NLPNEKIDYK
EAAGDE-------------------------------NENS---SSEKIE
EQRDIFHWRDLTY---SVKIKS-EDRVILNHVDGWVAPGQVTALMGASGA
GKTTLLNCLSERVTSGTI----TDGQRMVNGHGLDS--SFQRSIGYVQQQ
DIH-LPTSTVREALTFSAYLRQPYSVSRAEKDAYVDYIIDLLEMRAYADA
LVGV--AGEGLNVEQRKRLTIGVELVAKPKLLLFLDEPTSGLDSQTAWSI
CKLMRKL-ADHGQAILCTIHQPSAILLKEFDRLLFLQKGGKTVYFGDLGE
NCQELINYFEKYGAHPCPKEANPAEWMLEVVGAAPG--------------
-------SK-AS-QDYFEVWKNSTEYANVQKELDRMETELVKLP------
---RDESPDAKLTYAAPLWKQYFIVTWRTIQQDWRTPGYIYAKLFLVISS
SLFNGF-SFFK--AGTSQQGLQNQMFSMFMFFMPFQTMIQQMLPFYIKQR
EIYEVREAPSRTFSWAAFISAQITSEVPFQIAVGTLSFFCWYYPAGFYKN
AEPTDMVNQRGATMWLLVISFYVYTTTMGQLCVSFVELADNAANLANLLF
IM-CLNFCGVLAGPNFLPGFW-IFMYRCNPFTYLIQAMLSTALANTKVVC
ATRELLTFDP--TSG-KTCGEYMKDYIA-----------KAGGYLVNESA
----TSDCQYCKMAT--TNAF--LSSVNAK-YDQRWRNYGIFVAFICINI
ILTIFFYWLARVPKGTRQKK------------------------------

>O74676

-------------------------------MADADTSSNSSKTNEDRSQ
EGFGTYQGYTDRVASEVQELARIISHASIQQLKLKRQHSRQESQKSNEQE

SELSGKLGVIPVDENGNFVDQRLNPNSPEFNAAYWIQNAH-KLVSSDIDY
FKPVTIGVAYKNLRAYGSASDADYQSTLVNLIPKYLSLFFREYILRHT-G
---------PTFDILKPMDGLIKPGELTVVLGRPGAGCSTFLKTIASQTY
GYHIDKD--SVI------RYNSLTPH---EIKKHYRGEVV--YCAETENH
FPQLTVGDTLEFAAKMRTPQNRPL-----GVSRDAYARHLAAVVMAVYGL
SHTRNTKVGNDFIRGVSGGERKRVSIAEITLNNAMVQCWDNSTRGLDSAT
ALEFIRALKASADIVHTTPLVAIYQCSQDAYDLFDKVVLMYQGYQIYFGS
AKKAKQYFID-MGYEC-PQRQTTADFLTSLTNPAERIV---RQG-FEG--
KVPQTPQEFYEYWKKSPEGQQIVADVDQYLTEH-----------------
---SSAAEKEAIKEAHQARQSDHLKPASP--YTVSFFMQVRYIAHRNILR
IKGNPSIHLFQIFGNIGMSFILSSIFYNL----PTATSSFYHRTAALFFA
VLFNAFSCLL-EIFSLYEAR-SIVEKHKKYALYHPAADAFA-SIVTELPT
KFIIAIGFNLVYYFMVNFRRTPGNFFFYLLINFSATLAMSHIFRTIGAAT
KTLQEAMTPAAILLLALTIFTGFVIPTPNMH---GWCRWINYLDPLAYAF
ESLIANEFHNRDFEC--SQYVP----SG--GSYPTAG-PNRICTPV-GSV
PGQDFVDGTR-YMEMSFDYRNSHKWRNFGIVIGFIVFFFCTYILLCEINK
GAMQKGEILLFQQRALKKRKKANNDIESGEIEKVTPEFDNEYENNQD--K
M---LQ
SGGDTFFWRDLTY---QVKIKS-EDRVILDHVSGWVKPGQVTALMGASGA
GKTTLLNALSDRLTTGVV----TEGIRLVNGRPLDS--SFQRSIGYVQQQ
DLH-LETSTVREALEFAAYLRQPKSVSRKEKNEYVDYIIRLLEMEQYADA
VVGV--SGEGLNVEQRKRLSIGVELVAKPKLLVFLDEPTSGLDSQTAWSI
CKLIRKL-ADNGQAILCTIHQPSAILLAEFDRLLFLQRGGQTVYFGDLGK
NFTTLINYFEKYGAPKCPPEANPAEWMLEVIGAAPG--------------
---------SKANQDYYDVWLKSSEFQEMNSELDLMSEELVKKP------
---LDDDPDRLKPYAAPYWEQYLFVTKRVFEQNWRTPSYLYSKFLLVVTS
SLFNGF-SFYK--ADRSLQGLQNQMFSVFMFLVILHTLIQQYLPTFVSQR
DLYEVRERPSKTFSWITFIAAQVTAEIPWNIICGTLGYFCWYYPVGLYQN
ATYTNTVHQRGAFMWFAIVLFFIYTSTLAQLCISFLEIDDNAANLSVLLF
TM-CLAFCGVLVTKEQLPGFW-VFMYRCSPFTYLVSVMLSVGLVDAPVTC
AAKEYLRFSP--PQG-YTCMQYMEPYMK-----------VAGGYLLNENS
----TTECEFCTMKV--TNVF--LKMIGSD-YSKRGRDIGIYIAFIGINI
IGTFILYWFARVPKNFDIKLRRKR--------------------------

>Q6BHY4

--------------------------------------MNSSTHYDDVSQ
KSSTVNDSADDNKIPEYHGFDKEVSSEVQNLARMMSRPSIDKASSLARTL
SNMSQVPGLNPMAKDEGELDPRLDPDSDSFDSKFWVKNLR-KMHNSDPAY
YKPASLGVAYKDLRAYGIATDADYQANVGNVVYKTISQTIKGFFDKNNDD
---------AKFDILKPMDGLIRPGEVTVVLGRPGAGCSTFLKTISSNTH
GFTVAKD--SVL------SYDGLKPN---DIIKHFRGDVV--YCAETESH
FPQLTVGQTLDFAAKLRTPQNRPE-----GVSREEYAAHMTKVIMATYGL
SHTRNTKVGNDFIRGVSGGERKRVSIAEVALSFASLQCWDNSTRGLDSAT

ALEFIKALKTSATVLNATPMIAIYQCSQDAYDLFDKVILLYEGYQIFFGD
CKQAKLYFLE-MGYDC-PQRQTTADFLTSLTNPSERVV---RPG-YEN--
KVPRTPEEFYTYWQNSPERKALLGEIDDYLNKT-----------------
---DNEERLQQFKDAHNTKQSNHLRPASP--YTVSYGLQVKYIMGRNIMR
TKGDPSITLFSIFGNTVMGLIISSIFYNL----DDTTGSFYYRTVAMFFA
VLFNAFSSLL-EIFALYEAR-PIVEKHKTYALYHPSADAFA-SIITELPP
KLLVSISFNLVLYFMVNFRRNAGNFFFYLLVNFTATLSMSHLFRTIGSAT
KSLSQAMTPASVLLLALTIFTGFVIPTPEML---GWCRWINYLDPIGYAF
EALIANEFHGRDFDC--SQFVP----SG--PGYPTSG-NSIICSVV-GSQ
PGSDIVNGDD-YIRGSYEYYFSHRWRNWGIVVGFVVFFLFVHIIICEYNK
GAMQKGEILLFQRSALKKNKRQRKDIESGNIEKVGPEFNNEKTPDNEIDN
K---LP
SSGDIFHWRELTY---QVKIKS-EDRVILNSVDGWVKPGQVTALMGASGA
GKTTLLNALSDRLTSGVI----TSGVRMVNGHELDA--SFQRSIGYVQQQ
DLH-LQTSTVREALTFSAYLRQPKSVPKSEKDSYVDYIIRLLEMEKYSDA
VVGV--SGEGLNVEQRKRLTIGVELVAKPKLLVFLDEPTSGLDSQTAWSI
CKLIRKL-ADHGQAILCTIHQPSAILLKEFDRLLFLQRGGKTVYFGDLGD
NCQTLIDYFEKYGAPKCPPDANPAEWMLEVIGAAPG--------------
---------SHASQDYYDVWMNSTEYREVKGELDVMEQELVKKP------
---KDDSPESMKTFAVPMWQQYINVTHRVLQQYWRTPSYTYSKVLMSIFS
SLFNGF-AFFK--ANNTMQGLQNQMFSVFMFFVIFNTLTQQYLPNYVSQR
DLYEARERPSKTFSWVAFITAQITAEIPWQILTGTLAFFCWYYPIGLYGN
AEATDTVSQRGALMWIIIVLFFIYCSTMAQLCISFMEVADNAANLASLLF
TM-CLTFCGILASPDAMPGFW-IFMYRCNPFSYLVSAILSVALQDSSVTC
SDKELLRFEP--EGG-QTCGEYMQSYIQ-----------NAGGYLISNDT
----TGSCEYCTISS--TNVF--LESVSVD-KTKRSRDIGIFFCFIVINM
IGTVFFYWLARVPKRSRQK-------------------------------

>C5MBT4

---MSDKTQI
NREGNQLTVDNYGGFDAHILEDIQNLAREFTHESTTNGNND-----LQRL
HTNMSEVPGINPYTDEAHSREKLNPESDNFNAKLWVKNLR-KLYDSDLEY
YRPTKLGVAYSNLRAYAVANDYNYQPTVANVFWKLINDGVHYLKKHDN--
----SIY----FDILKPMDGIMKPGELTVVLGRPGAGCSTLLKTLASQTY
GFHIGKE--SKI------IYDGISST---KIENHYRGNVI--YSAETDAH
FPHLTVGQVLEFAARMRTPQNRGE-----GVDRETFAKHMASVYMATYGL
LHTRNTNVGNDFVTGVSGGERKRVSIAEASLSGANVQCWDNATRGLDAAT
ALEFIKALKTSAVILESTPIVAIYQCSQDAYDLFDNVVVLYEGHQIFFGK
ANKAKEYFIR-MGYDC-PQRQTTADFLTSLTNPSERRA---LPG-YEN--
KVPRTPEEFEAYWKRSPEYTMLINEIDSYFIEC-----------------
E---KLNIKQTYHESHVARQSNHTRPTSP--YTVSFFMQVKYVMQRNFLR
MKADPSIPLIMIFSQLVMGLLLASVFFNL----KKNTTTFYYRSGSLYFA
LVFNAISSLL-EIVALFEAR-PIVEKHKKFALYRPSADALA-SIITELPV

KLSMSLCFNIPFYFMVGLRSGAGRFFFYWLIGILCTLVMSHIFRSIGSMF
TSLAGAMTPAGVILLAIIVFTGFVIPYPSML---GWSKWIKWINPVSYMF
ESLMVNEYHDREFPC--GQIVP----SGS-G-YDNLPLEYKVCSSI-GGI
PGSDVVRGDD-YLRVGFEFFNSHKWRNFGISFAFAVVLLFLYIALTELNK
GAIQKGEIVLFLRSSLKKHKRRFTEFDIESGSTVE-------NISIQIVQ
KASIEQ-------------------------------NSIR---DSCNLP
SDKEIFFWKDLTY---QVKIKR-EDRVILDHVDGWVKPGQITVLMGASGA
GKTTLLNCLSNRVTTGVI----TAGVRMVNGHELDS--SFQRSIGYVQQQ
DIH-LQTSTVREALQFSAYLRQSNKISKKEKDEYVDYVIDLLEMTNYADA
LVGV--AGEGLNVEQRKRLTIGVELVAKPKLLLFLDEPTSGLDSQTAWSI
CKLMRKL-ADHGQAILCTIHQPSAIIMAEFDRLLFLQKGGQTVYFGALGD
NCQTMIDYLENHGADPCPKDENPAEWMLEIVGAAPG--------------
-------SH-AK-QDYFEVWRNSNEYEAVRNQLNRMETELVMLP------
---RDEDPETLLKYAAPIWKQYLLVSWRAIVQDWRSPRYIYSKFFLIVIS
SIFIGF-SFFK--AKNDIQGLSNQMLAVFMYTVPLTTIVDQVLPFFVRQR
EVFEVREAPSRTYSWFAFITGQVTSELPYQIVVGTIAFFCWYYPVGLYEN
AEPTHTVTGRGVLLWLFITSFYVFASTFGQLCISFNELIENAGCLSATLF
LL-CLMFCGVLAVPESMPRFW-IFMYRCTPFTYWIQGVLATGLAHNKVEC
SSREFLVVTP--PVG-QTCSSFLDPYIS-----------VAGGYYLV-NE
----KEGCSFCAMNS--TDQF--LNSIHAL-YSERWRNFGIFISFIVSNI
VLAVFLYWLARVPKGAKSSTRK----------------------------

>Q4WDV4

---MALNSTD
NRWSTGEDTPSEAQLPDGEERLDAAPDEKVTAEDIDRRLTNLVRKISAQS
RRSHH------SFLFGAGENSSLNPQSPSFDARKWARAFY-NARYRQDDG
HPPRVVGVAFKNLNVFGYGSPVDYQMSVGNALLKVPTMVRQALG-GGK--
-----QR----VDILHDVEGLVLPGEQLCVLGPPGSGCSTFLRTIAGETH
GLNVDAA--SYI------NYHGISPK---QMSTAFRGEAI--YTAEVDAH
FPMLSVGDTLYFAALARAPQVIPG-----GLSRQEYAKHLRDVIMAMFGI
GHTINTRVGNDFVRGVSGGERKRVTIAEAALGYSPLQCWDNSTRGLDSAN
AVEFCRTLRTQSDVFGITSCVAIYQAPQAAYDLFDKVLVLYEGWQIYFGA
AHEAQAYFEQ-LGFQC-PESQTTADFLTSMCSPAERIV---KPG-FEH--
MAPRTPEEFAQRWKESPQRQSLLHAIEKYSTEH-----------------
PL--DGPDLHQFALSRRAEKSHRQREKSP--YTLSYRGQVKLCLWREWQR
LKNDPSVTLAMLIGNFFEALIIASIFYNL----TGDTSSFYYRGALLFMM
VLLNAFASVL-EILTLYEKR-TIVEKQSRYAYYHPSAEALS-SFIMSLPY
KFVNSSLVNLTLYFMSNLRREPGPFFFFLLISTSMMLAMSMFFRWFASLT
KTIDQALAPSSIILLALVLYTGFTIPVSYMR---GWASWIRWLNPVSYGF
EAVMINEFHGREFPC--SSFVP----SGP-G-YEDVSRTQRVCSTV-GAT
SGSDVVSGDV-FVRSSYGYVNSHRWRNFGIIIAMTVFLAVCHFVTTELVA
SKRSKGEVLVFRRGSAHIARAKQGQRDEEQPSASAVPSEKYSEAPTPVEG
--VE

TQTSIFHWEDVCY---DVKIKN-ETRRILDHVDGWIKPGTLTALMGVSGA
GKTTLLDVLASRTTVGVV----T-GETLVDGRQRDS--SFQRKTGYVQQQ
DLH-LATTTVREALEFSALLRQPPQYSREEKLEYVEKVIDLLHMRDYADA
IVGV--PGEGLNVERRKRLTIGVELAARPKLLLFLDEPTSGLDSQTSWSI
CNLMETL-TRNGQAILCTIHQPSAMLFQRFDRLLLLAKGGKTVYFGEIGS
GARTLMDYFVRNGGPPCPKGANPAEHMLEVIGAAPG--------------
-------AH-TD-IDWPAVWRNSPEYQQVRQELSRLRQLASQPSS-----
--VHSDDPSSYSEFAAPFPAQLGQVGRRVFQQYWRTPSYLYSKAILTVGS
SIFIGF-SFFK--GDNTAQGLQNQVFGVFVFLFVVIQLIFQIIPTFVTQR
TLYESRERQSKTYSWQAFVLSNIAVEFAWNTIAAVLCFLAWFYPVGLYRN
AEYTDSVHSRSTLVFLIIWATFLFASSFAHLLIAGVESAELASALANIMG
IM-MYAFCGILAGPHALPGFW-IFMYRVNPFTYLVSGLLSASLGDAPMHC
AANEFLAFSP--PAN-RTCGEYMEDYMA-----------LAGGYLLDSAA
--RGDEQCQYCRVDN--TSQY--LRNFSID-FATRWRDFGLLWVYVAVNT
FGAVFLYWLCRVPKGKKRL-------------------------------

>B8N6A8

-----------------------------------MFSSPGHEPPSSDMV
LAMDGGTSSGTQTYANSERSDLEQKDKLAENRAEIDEQLTNLARKLTTKS
EQLHH------RSPFEAPEGGCLDPNSPNFRARDWAKAFY-NARYNADEN
CPPRVAGVAFKNLNVSGYGSPVDYQMSVGNALLKLPTQVYQFLG-GKK--
-----RK----INILQGLDGLVLPGEQLCVLGPPGSGCSTFLKTIAGETH
GFQVDPA--AYI------NYHGITPK---QMSTDFRGEAI--YTAEVDAH
YPQLSVGDTLYFASLARAPRHLPG-----GISSQEYATHLRDVIMAMFGI
SHTINTRVGNDFVRGVSGGERKRVTIAEAALSYAPLQCWDNSTRGLDSAN
AVEFCRTLRTQSDVFGMTSCVAIYQAPQAAYNLFDKVIVLYEGHQIYFGT
AHDAKSYFER-LGFLC-PESQTTADFLTSMSSPTERIV---RPG-FES--
LAPRTPEEFAKLWKASPERQSLLRQIDQYATEH-----------------
PF--DGADLDRFSQSRKTEKSKNQRQKSP--YTLSYWGQIRLCMWRELQR
LKNDPSVTIVMLINNFFEALIISSIFYNL----SGNTSSFFSRGAILFMM
VLLNAFSSML-EILSLYAKR-TIVEKHNRYALYHPSAEAIS-SMIMDMPY
KIVNSILMNITLYFMANLRREPGPFFFNYLISFMMVMSMSMFFRLFASLT
KTIQQALAPSSIILMALVLYTGFAIPVSYMR---GWASWIRHLNPVAYGF
EAIMVNEFHGRTFPC--ASFVP----SGV-G-YENISKDERVCSVV-GSV
PGSDLVDGTT-FVKSTYGYENSHRWRNFGIILALTIFLALCQIIATELVA
SERSKGEVLVFRRGSSQKARAKQHQHDEERTQAPVIQNEKHSEGPDSTIG
--VE
KQTSIFHWENVCY---DVKIKS-ETRRILDHVDGWIKPGTLTALMGSSGA
GKTTLLDVLANRTTVGVV----G-GDMLVDGRPRDS--SFQRKTGYVQQQ
DLH-LHTSTVREALEFSALLRQPPQYTREEKLDYVEKVLDLLNMRDYADA
IVGI--PGEGLNVEQRKRLTIGVELAARPKLLLFLDEPTSGLDSQTSWSI
CNLMETL-TKNGQAILCTIHQPSAMLFQRFDRLLLLAKGGKTVYFGDIGR
ESRILMDYFTRNGGPALPPGSNPAEHMLEVIGAAPG--------------

-------AK-SE-IDWPAVWRNSPEYQNVRHELSNLRALANQPSP-----
-VSDTNDKSSYAEFAAPFATQFVQVGLRVFQQYWRTPAYIYSKVLLTIGC
SLFIGF-SFFR--ADNTAQGLQNQMFGVFVFLFVVIQLIIQIIPSFVTQR
TLYEARERQSKTYSWQAFVVTNILVELAWNSIMAIFCFLVWFYPVGLFHN
AEYTDTLHYRSTLTFLFIWVTFLFASSLAHMLIAGIESEEIASSLSNILA
IM-MYAFCGILAGPDALPGFW-IFMYRVNPFTYLVSGLLSTSLGEAPMHC
AEKEFLSFST--PAN-LTCGEYMQDYIS-----------TNGGYLLNSKA
--QGGEDCHFCATGN--TTQF--LQHVNID-FSTRWRDFGLMWVYVVFNI
FAAISLYWLCRVPKGKKNK-------------------------------

>Q2UCF2

--MA
LAMDGGTSSGTQTYANSERSDLEQKDKLAENRAEIDEQLTNLARKLTTKS
EQLHH------RSPFEAPEGGCLDPNSPNFRARDWAKAFY-NARYNADEN
CPPRVAGVAFKDLNVSGYGSPVDYQMSVGNALLKLPTQIYQFLG-GKK--
-----RK----INILQGLDGLVLPGEQLCVLGPPGSGCSTFLKTIAGETH
GFQVDPA--AYI------NYHGITPK---QMSTDFRGEAI--YTAEVDAH
YPQLSVGDTLYFASLARAPRHLPG-----GISSQEYATHLRDVIMAMFGI
SHTINTRVGNDFVRGVSGGERKRVTIAEAALSYAPLQCWDNSTRGLDSAN
AVEFCRTLRTQSDVFGMTSCVAIYQAPQAAYNLFDKVIVLYEGHQIYFGT
AHDAKSYFER-LGFLC-PESQTTADFLTSMSSPTERIV---RPG-FES--
LAPRTPEEFAKLWKESPERQSLLRQIDQYATEH-----------------
PF--DGADLDRFSQSRKTEKSKNQRQKSP--YTLSYWGQIRLCMWRELQR
LKNDPSVTIVMLINNFFEALIISSIFYNL----SGNTSSFFSRGAILFMM
VLLNAFSSML-EILSLYAKR-TIVEKHNRYALYHPSAEAIS-SMIMDMPY
KIVNSILMNITLYFMANLRREPGPFFFNYLISFMMVMSMSMFFRLFASLT
KTIQQALAPSSIILMALVLYTGFAIPVSYMR---GWASWIRHLNPVAYGF
EAIMVNEFHGRTFPC--ASFVP----SGV-G-YENISKDERVCSVV-GSV
PGSDLVDGTT-FVKSTYGYENSHRWRNFGIILALTIFLALCQIIATELVA
SERSKGEVLVFRRGSSQKARAKQHQHDEERTQAPVIQNEKHSEGPDSTLG
--VE
KQTSIFHWENVCY---DVKIKS-ETRRILDHVDGWIKPGTLTALMGSSGA
GKTTLLDVLANRTTVGVV----G-GDMLVDGRPRDS--SFQRKTGYVQQQ
DLH-LHTSTVREALEFSALLRQPPQYTREEKLDYVEKVLDLLNMRDYADA
IVGI--PGEGLNVEQRKRLTIGVELAARPKLLLFLDEPTSGLDSQTSWSI
CNLMETL-TKNGQAILCTIHQPSAMLFQRFDRLLLLAKGGKTVYFGDIGR
ESRILMDYFTRNGGPALPPGSNPAEHMLEVIGAAPG--------------
-------AK-SE-IDWPAVWRNSPEYQNVRHELSNLRALANQPSP-----
-VSDTNDKSSYAEFAAPFATQFVQVGLRVFQQYWRTPAYIYSKVLLTIGC
SLFIGF-SFFK--ADNTAQGLQNQMFGVFVFLFVVIQLIIQIIPSFVTQR
TLYEARERQSKTYSWQAFVVTNILVELAWNSIMAIFCFLVWFYPVGLFHN
AEYTDTLHYRSTLTFLFIWVTFLFASSLAHMLIAGIESEEIASSLSNILA
IM-MYAFCGILAGPDALPGFW-IFMYRVNPFTYLVSGLLSTSLGEAPMHC

AEKEFLSFST--PAN-LTCGEYMQDYIS-----------TNGGYLLNSGA
--QGGEDCHFCATGN--TTQF--LQHVNID-FSTRWRDFGLMWVYVVFNI
FAAISLYWLCRLPKGKKNK-------------------------------

>A1C5R3

---------------------------------------MARKELRSYPS
RKVDSQSGTSVNDSTDSLDTPRTKDLLNGKGPGQIDERLTDLARKISTAS
HQPNH------SFMFDVRDDSPLNPLSSSFNARKWAHEFY-NVKYSGNDG
SGPRAAGVAFTNLNVFGYGSPVDYQMSVGNALLKVPTMLRQALG-GKK--
-----QR----INILRDIEGLVLPGELLCVLGPPGSGCSTFLRTIAGETH
GLNIDSA--AYI------NYHGISPQ---DMLTAFRGEAT--YTAEVDAH
FPMLSVGDTLYFAALARAPRTIPG-----GMSRSEYARHLRDVIMAMFGI
GHTLNTRVGNDFVRGVSGGERKRVTIAEAALGYAPLQCWDNSTRGLDSAN
AVEFCRTLRTQSDVFGITSCVAIYQAPQAAYNIFDKVIVLYEGRQIYFGP
AAEAKKYFES-LGFHC-PAFQTTADFLTSMSSPSERIV---KEG-YER--
HAPRTPEDFAQRWKESRERQALLDQIEAYRNEH-----------------
PL--NGKDLEEFSLSRQREKSKHQRHKSP--YTLSYWGQIKLCMWREWQR
LRNDPSVELAMLIGNFFEALIIASIFYNL----PVNTSSFFYRGALLFML
VLLNAFAGVL-EIFTLYEKR-TIVEKQSRYAYYHPSAEAIS-SLIMSFPY
KITNALLVNLTLYFMSNLRREPGPFFFFLLISFSMMMGMSMFFRWFASLT
KSIDQALAPSSIILLALVLYTGFAIPVSYMR---GWAAWIRWINPVAYGF
EAVMVNEFHGREFPC--MKFVP----SGP-G-YEGVSSTARVCSVV-GSV
PGSDVVQGTA-FVQSSYGYENSHRWRDFGIIVAMIIFLAVCHLITTELVA
SKRSKGEVLVFRRGSAHVSRLKQTQSDEERPVPSTIWSEKGIDEINPVSG
--VE
KQTSIFHWEDVCY---DIKIKD-EPRRLLDHVDGWIKPGTLTALMGVSGA
GKTTLLDVLASRTTMGVI----T-GDMLVDGRQRDG--SFQRKTGYVQQQ
DLH-LATSTVREALEFSALLRQPSQFSHAEKLAYVETVIDLLHMREYADA
IVGV--PGEGLNVEQRKRLTIGVELAARPKLLLFLDEPTSGLDSQTAWSI
CNLMETL-TKNGQAILCTIHQPSAMLFQRFDRLLLLAKGGKTVYFGEIGP
ESRTLMDYFARNGGPSCPPGSNPAEHMLEVIGAAPG--------------
-------AH-TD-IDWPAVWRNSPEYQQVHRELSQLRQLADTSSR-----
--MDSADSSNYGEFAASFPAQVYEVGLRVFQQYWRTPSYIYSKALLTIGS
SLFIGF-SFFK--ADNTAQGLQNQMFGVFVFLFVIIQLILQIIPTFVTQR
TLYESRERQSKTYSWQAFVLSNIFVEIAWNSLSAVFCFLVWFYPVGVYRN
AEYTDTVHSRSMLVFLIIWAAFLFASSFAHLLIAGVSSAEIASALSNIMS
IM-MYAFCGILAGPHALPGFW-IFMYRVNPLTYLVSGLLSASVGDAPMHC
AENELLAFVP--PAN-QTCGEYMESYMG-----------SRGGYLLSSVA
--R--DECRYCQVDN--TNQF--LANFSID-FSTRWRDFGLLWVYIAVNT
AGAMFLYWLCRVPKRRKGQ-------------------------------

>Q0CSU1

------------------------------MTLPLPPKENSWRSRLSDMH
ASSDTIDSQTQLFDSTGSSAVKGATRGAKRDLPWDEAGVHNLAQVLTAQS
VSPDC------DSPFDPNNQTCLNPNSPEFKARDWAKAFY-NIR-YTAEG
LSPRMAGFAFRNLNVHGYGTATDYQMSVGNAALKLGSNLANLLGITKK--
-----PR----VNILQGLDGLVLPGEQLCVLGPPGSGCSTFLKSIAGDTL
GLEVDPE--TFL------NYNGITAK---QMMTRFRGEAI--YTAEVDQH
FPMLTVGETLYFAALARAPRTIPG-----GLSREEYATHLRDVIMAMFGI
SHTKDTRVGNDFVRGVSGGERKRVTISEAALSYAPLQCWDNSTRGLDSAN
AIEFCKTLRLQSDIFGTTSCVAIYQAPQAAYELFDKVIVLYEGRQIYFGR
AYEAKAYFEA-LGFEC-PESQTTPDFLTSMTSPTERVV---KPG-YEH--
LVPRTADEFAQRWRDSPERRDLLNQIQRYTVDH-----------------
PF--DNADMRRFAEARKSEKSTKQREKSP--YTLSYWRQIQLCIWREFRR
LKADPGVSIFSMVLSLAQFTIIASVFYNL----QPVTASLTQRGALMFMM
IVMTALSSML-EIMALYEKR-SVVEKHSRYALYHPSAEAIA-SSVTDIPS
KILTCVCSNIVIYFMANLRRTPEAFFFYLLTAFTTLMCMSMFFRFFASIT
KTIAQALAPVAIINLGLSLYTGFAIPTPYIL---GWSKWIKYINPISYAF
EAIMVNELHGLDLPC--SSFVP----SGP-G-YEDVDPMGRSCSTV-GSV
PGSPNVSGDA-FLEQSYEYAWSHRWRDFGILVGFTVFFTMFYLIGSELVT
GERSKGEVLVFRRNHMHHNRVRRQQVDEEQTEPGSPFSEKAEKSSAQFSQ
--VE
KQTAVFHWQNVCY---DITANG-EQKRLLDNVDGWIKPGSLTALMGVSGA
GKTTLLDVLASRKTTGVI----T-GDMLVDGRQRDG--SFQRKTGYVQQQ
DLH-LSTCTVREALEFSALLRQPSHYTHQQKLDYVDTVIDLLGMEEYADA
VVGV--PGSGLNVEQRKRLTIGVELAARPKLLLFLDEPTSGLDSQTSWSI
CNLMEKL-TNNGQAILCTIHQPSAMLFQRFDRLLLLASGGKTVYFGEIGR
NSKILMDYFVRKGGAPCPPGVNPAEHMLEVIGAAPG--------------
-------AK-SD-IDWPTVWRESEEYREVHRELERLRELANHPSA-----
-VVDPNDKSIYEEFAAPFTTQAYLVTLRVFQQMWRTPSYIYSKILLCLGC
ALFIGF-SFFN--SNNTAQGLQNQMFGTFLFLIIYVQLIQQIMPMFVTQR
TLYEARERQSKTYCWQSFILSNLFVELAWNTLMAVGSFLVWYYPMGVWRN
ALYTDALNSRSMLAFLVIWEGFLYATSASYLFIAGMDSDQAAGGVSMLCN
IM-GFIFCGILASPDTLPRFW-IFMYRVSPFTYIVNSFLTTALANAPMYC
SPAEMLSFAA--PQN-QTCAQYMASYME-----------TAGGYLVNPDT
KGGGTENCQFCAMDS--TNQF--LKNVNLN-FENRWRDLGIVWAYIVFNT
AGAIFFYWLVRVPKGKKLKSS-----------------------------

>B6QUR9

-----------------------------MSLAINPNPPQSQSVVSHNNT
TQEHGATANDVPVDAAADLAKRSSDETLMERDEIVDAQVGELARQLTRQS
TRFAAKGG-LQNPFVIDDPESSLNPHSPNFRARDWIKMLF-ELRSQDRGT
FPEKQAGISFRNLSVHGFGSPTDYQKNVLNSLLEIGTLVRRAVG-AKM--
-----QT----VHILRDFEGLVRSGEMLVVLGRPGSGCTTLLKTIAGEMN
GINMSED--AVV------NYQGVPVK---EMHNNFKGEAI--YTAETDVH
FPQLTVGDTLKFAALAKSPRNRFE-----GVTRDQHATHMRDVIMAMLGL
SHTINTRVGNDFVRGVSGGERKRVSIAEAALSEAPLQCWDNSTRGLDSAN
ALEFCKNLALMSKYASTTACVAIYQASQSAYDCFDKVTVLYEGRQIYFGG
ATEAKQFFVD-MGFEC-PERQTTADFLTSLTSPQERKV---RPG-FEG--
RVPETPDQFVTAWKNSKARAKLMREIEQFESQY-----------------
PL--GGSSRDAFIDARRAAQSKRQRTMSP--YTISVWDQIALCTHRGFQR
LKGDASLTLSGLIGNFILALIVASVFYDL----GEDTASFYGRGALLFYA
VLLSGFSSAL-EILTLYAQR-PIVEKQARYAFYHPFTEAIA-SMLCDSPY
KIINSFTFNIPLYFMTNLRRTADAWWTFWLFSVVTTYTMSMLFRTLAATS
RSLSQALVPAAVLILGMVIYTGFVIPTKYML---GWSRWMNYINPIAYSF
ESFLVNEFANRDFNC--SVMVP----SGG-A-YDSVPLQYRSCSTV-GAA
AGSNTVQGSV-YLEESFAYVKGHEWRNLGILFVFMAGLAAAYLLSTEYIS
EVKSKGEVLLFRRGHKPTNLAFPGSSSDLESSVGGAVSEKKVSGLVTAGT
SSSTSH-------------------------------AGTATPPAEVQIQ
RQTAIFHWQDVCY---EVKIKS-ETRQILDHVDGWVKPGTCTALMGVSGA
GKTTLLDVLATRVTMGVV----T-GEMLVDGRARDQ--SFQRKTGYVQQQ
DVH-LPTSTVREALQFSALLRQPGHLSRKEKLNYVDEVIKLLDMEAYADA
VVGV--PGEGLNVEQRKRLTIGVELAARPQLLLFLDEPTSGLDSQTSWSI
LDLIDTL-TRHGQAILCTIHQPSAMLFQRFDRLLFLAKGGKTIYFGDIGE
NSKVLSSYFERNGATPLSQGENPAEWMLEVIGAAPG--------------
-------SH-TD-IDWPVVWRKSPEHTKVKEHLAELKATLSVKEP-----
-APQSDDPGAFREYAAPFRVQLWETMKRVFSQYNRTPIYIYSKLALSVLS
ALYVGF-SFFH--AKNSIQGLQNQMYSVFMLMTVFGNLCQQIMPLFVTQR
AIYEVRERPAKTYSWQAFMISNIFVELPWNTLMAVLMFVCWYYPIGMYNN
AKPTDSVTERGGLMFLFVWVFLLLTSTFAHLIVAGIETAETAGNIVTLLF
SL-CLIFCGVLATPQAMPRFW-IFMYRVSPLTYLVQGMLSTGLSGTTVEC
ADVEYITFDP--PPGFSTCIDYMGGFIN-----------ATGGYLREPDA
----TSNCQYCIISS--TDTY--LAAINTY-FSQAWRNFGLIWVFIIFNI
AGATLIYWLARVPKGKKFAGSS----------------------------

>B8MQM1

-----------------------------------MSFAINPNPHQSTSV
FSHNTAPVHETTAEDASDLEKKSSDETLMEREEIVELRVGELARQLTRQS

TRFSAKGGSLQNPFLTDDPESTINPHSPNFRARDWIKMFL-EMRHQDPER
YPEKHAGISFRNLNVHGFGSPTDYQKDVLNMLLEVGTLVRWAFG-VKM--
-----HK----VQILRDFEGLVKSGEMLVVLGRPGSGCSTLLKTIAGEMN
GINMSED--AVV------NYQGVPVK---EMHNNFKGEAI--YTAETDVH
FPQLSVGDTLKFAALARSPRNRFE-----GVTRDQYATHMRDVVMAMLGL
SHTINTRVGNDFVRGVSGGERKRVSIAEATLSLAPLQCWDNSTRGLDSAN
ALEFCKNLALMSKYASTTACVAIYQASQSAYDCFDKVTVLYEGRQIYFGP
TTEAKQFFVD-MGFEC-PDRQTTADFLTSLTSPAERRV---RPG-FEG--
RVPETPDEFAAAWKKSEARAKLMREIEAFEAQY-----------------
PL--GGSSRDAFIDARRATQAKRQRSMSP--YTISVWEQISLCTVRGFQR
LKGDSSLTLSGLIANFIVALIVASVFFNL----GDDSNSFYGRGALLFYA
VLLSGFSSAL-EILTLYAQR-PIVEKQSRYAFYHPFTEAIA-SMLCDTPY
KVLNSFTFNIPLYFMTNLRRTASAWWTFWLFSLVTTYTMSMLFRTIAATS
RSLSQALVPAAILILGMVIYTGFVIPTKYML---GWSRWMNYINPIAYSF
ESLLVNEFADRDFAC--SVMVP----SQG-P-YDSVPMQYRSCSTV-GAS
AGSSTVSGSA-YLKLSFDYQKSHEWRNLGILFAFMIFFCGVYLVATEYIS
EIKSKGEVLLFRRGHKPANLSFPGSSSDLESSIGGISEKKASGSAPGTAN
SESILN-------------------------------AGTATPPAEAKIQ
RQTAIFHWEDVCY---DIKIKG-EPRRILDNVDGWVKPGTCTALMGVSGA
GKTTLLDVLATRVTMGVV----S-GDMLVDGRHRDQ--SFQRKTGYVQQQ
DVH-LPTSTVREALEFSALLRQPGHLSRKEKLDYVDEVIRLLGMESYADA
VVGV--PGEGLNVEQRKRLTIGVELVARPQLLLFLDEPTSGLDSQTSWSI
LDLIDTL-TRHGQAILCTIHQPSAMLFQRFDRLLFLAKGGKTIYFGDIGK
NSSILSSYFERNGAAPLPQGENPAEWMLEVIGAAPG--------------
-------SH-TD-IDWHKVWRESPEYVKVKEHLAELRSTLSLKEP-----
-EPQANDPGAYREYAAPFSVQLWETMRRVFAQYYRTPVYIWSKFALCVLT
TLYIGF-SFFH--AKNTIQGLQNQMYSVFMLMTIFGNLCQQIMPLFVTQR
SLYEVRERPAKTYSWQAFMMSNIIVELPWNTLMSVLMFLCWYYPIGLYNN
AKPTDAVTERSGLMFCLIWVFMLFTSTFAHMVIAGIENAETGGNIATMLF
SL-CLIFCGVLATPQAMPGFW-IFMYRVSPFTYLVQGMLSTGLSGTHVQC
SSVEYLTFDP--APGFSTCIDYMKDYID-----------LAGGYLLDNNA
----TSHCQYCTIGE--TDTF--LASVNAF-FSQAWRNFGLMWVYIGFNI
VAAVGIYWWARVPKGKKFSGSS----------------------------

>O42690

----------------------------------MAKTSQAEGQPYKGYY
NNKSQGQPYHGYYSGFNKSASAQIHHLARSLTQGVQSHYDDTYTTATMHP
NGINP---------ISDKTDPTLDPESPSFSSKRWVQNMW-KLYQSDSEY
YKPGKLGVAYKNLRVYGDAIESDYQTTVSNGVLKYARNIFNKFRKDND--
----DYS----FDILKPMEGLIKPGEVTVVLGRPGAGCSTFLKTIACRTE
GFHVADG--SVI------SYDGITQD---EIRNHLRGEVV--YCAETETH
FPNLTVGETLEFAALMKTPQNRPM-----GVSREEYAKHVVDVVMATYGL
SHTKNTKVGNDFIRGISGGERKRLSIAEVTLVQASIQCWDNSTRGLDAAT

ALEFISSLKTSASILNDTPLIAIYQCSQNAYDLFDKVIVMYEGYQIFFGS
SQRAAAYFKK-MGFVC-QDRQTTPDFLTSITSPAERII---KPG-YER--
LVPRTPKEFYRYWRRSPERQALLEEIDEYLDNC-----------------
E---NYDQKQKIFEANNAKKAKHTYNKSS--YTVSLPMQVRYIMKRYWDR
MRGDIIVPLSTVAGNIAMALILSSVFYNL----QPNSSSFYYRTSVMYYA
LLFNAYSSVL-EIYNMYEGR-AIVQKHREYALYPPMADAIG-SIISDFPL
KVVCSVLFNLILYFMVNFKREPGAFFFYLLISFCSTLFMSHLFRTIGAFT
NSLAEAMTPSSLLLFALSTFSGFAIPVTYML---GWCKWIRWVNPLAYAY
EALISNEFHGRVFDC--SNIVP----SGF-G-YPKTG-NSVVCASI-GAL
PGEFKVDGDL-YLKLAFDYSYSNVWRNFGVLMAFIIFLFGTTIFFVQTNK
SSISKGETLVFRRKNIRKMRKMEE-DEEAYMDGMAPLDFSGSTEISDYSY
DYMDRK--LL
DTSNIFHWRNLTY---TVKIKS-EERVILNNIDGWVKPGEVTALMGASGA
GKTTLLNALSERLTTGVI----TSGTRMVNGGELDS--SFQRSIGYVQQQ
DLH-LETSTVREALKFSARLRQPNSVSIAEKDSYVEKIIDLLEMRTYVDA
IVGV--PGEGLNVEQRKRLTIAVELVARPKLLVFLDEPTSGLDSQTAWSI
CKLIRKL-ANHGQAILCTIHQPSAILLEEFDRLLLLQK-GETVYFGEFGA
NCHTLIEYFERNGASKCPQHANPAEWMLGVIGAAPG--------------
-------TQ-AN-QDYFETWRNSPEYRAVQNELHRLEEMPGLAS------
---GEKEPDTNQAYAASFWKQYIFVVHRLFQQYWRTPSYIYSKFAMAVLC
SLFNGF-TYYK--SQNSMQGLKNQMLSIFSMFVVLTTLAQQYVPLFVTQR
DLYEARERPSKTFSWLAFIAAQITAEIPYQVLAATISFFSWYYPVGLYRN
AVYSGAVTHRGVLMWLIMTLMFIYSSTLAQFCISWNQLADYAANWISLLL
TI-SMIFCGVIATKDSMPKFW-VFLYRCTPLTYLTSAMMSIGLGDSFVKC
APTEILTFPPQTPGV-QKCQDYMGAYIS-----------IAGGYLLNPEA
----TDNCKFCIMDK--TNQF--LDFMNIS-IHNFGRDTGIFIVFIVFNM
AATVFSYWLFRVPKGNREKGSFFDK--------LPFLNGGG---------
-----------DTNHENV--------------------------------

>Q68AM7

-------------------------------MSPQAETISTEAWSTDSAD
ERPWSGTTNQLDPVFEKEGDSTDISERDRAQIGILARQLTTASTKTGVQG
AVSNP---------FKGSNDPRLDPFSKEFNPKKWVETVL-HLNSQNPER
YLPRKAGFSFRNLSVHGFGSPVSYQKDFLNIFLQVADMVTGLI--NSK--
----DQK----IQILRNHNGLLRNGEMLLVLGRPGSGVSTFLKTIAGQTK
GLYLDEA--SEF------NYQGIPRK---KIRGEFRGDVI--YQAETDTH
FPNLTVGQTLLYAALAKTPQNRLS-----GVSRDEYARHIRDVTMAVFGL
THTMNTKVGNDFIRGVSGGERKRVSIAEVCLAQSPIQCWDNSTRGLDSAT
ALKFVQTLRLSADITSMATVVALYQASQQSYETFDKVAVLYEGRQIYYGP
VELAKNYFVN-LGYHC-PDRQTTPDFLTSLTNPVERVA---RSG-FEA--
KVPRSPDDFAKAWEESSLHKELMQDIVEFEKEH-----------------
PV--GGPAVDSFKKSRQAEKASWMTPNSP--YTISVPLQVLLCIRRGFRR
IQGDMTFFSITIGGNLVISLLLGSVFYML----EDTSASFQSRCILLFFA
LLFNALNSSL-EILSLYAQR-SIVEKHATYAFYHPLSEAVA-SMICDIPS

```
KILSTIAFNIPLYYMANLRTESGHVITYLLIVFSSTLVMSMIFRTIGQST
RTIAEALTPAALMVIAMVLYTGFILPIRNMQ---GWLRWLHYINPLAYSY
EALVANEFHGRNFEC--AGFIP----AGP-M-YQNITAADRTCSVV-GAS
AGSSVVRRRPLYCYELWVLLLQHVWRNFGILIAFIICFMIGYLLSAEYIS
SDVGKGEILLFQRSHFSAIKKTQKADEEVGSSGLHEKY------RQDETN
GEASTG------------------------------------------IT
AQKNIFHWRDLCY---EVSIKG-KTRRITDHVNGWVKPGKLTALMGASGA
GKTTLLDVLANRVTMGVV----T-GGIYNNGLPRDA--SFQRRIGYVQQQ
DLH-LETATIREALEFSAFLRQPAHVSKAEKLQSVEEILDLLEMRSYADA
VVGV--PGEGLNVEQRKRLTIGVELAAKPDLLFFLDEPTSGLDSQTAWSI
LLLLRKL-TDHGQAILCTIHQPSSMLFQQFDRLLLLAAGGRTVYFGDIGE
NSKTMTGYFERHGADHCDENDNPAEWMLRVIGAAPG--------------
-------SA-TK-IDWPATWLGSQEYADVKEELISLERKDG-VE------
---TNSSADPSLQFASPFHVQLWACTKRVFEQYWRTPSYLYSKLTMCFVT
ALFIGL-SFLQ--TKVTELGLQHQMFAVFMLLVIFPFLAYQQMPNYILQR
DLYEVRERPSKTYSWITFILAQVIVEIPWNSLAALITFIPFYYLIGMNHN
AAPTHQTTERGGLMFLLIWGFLMHCGTFTTMVVASAATAEIGAILALLLF
VF-CLIFCGVMATPASLPGFW-IFMYRVSPLTYIISGMMSTGLANIDVHC
SDIETTLVQP--PSG-ETCGSYLGAYLQ-----------IAGGAIYNPNA
----TSDCQFCTIAD--SNVF--LESVSSS-YTDRWRNFGLIWAYVAFNV
AATLILYWYVRVRGSPGLSHVVSWV--------QKASKHLS---------
-----------WKKAQ----------------------------------
--------------------------------------------------
--------------------------------------------------
-----------------------------
>A2R253
--------------------------------------------------
--------------------------------------------------
--------------------------------------------------
--------------------------------------------------
--------------------------------------------------
--------------------------------------------------
--------------------------------------------------
--------------------------------------------------
--------------------------------------------------
-MTVPGNSGTNPDGLNAEPYQGDISTEDQRKIMKLARRLTAESTRSRHDG
MPPNP---------FTTSCDPSLDPNSPKFNARHWARTIL-HWSSQDPDR
FPQRTAGVSFRSLGVHGYGRSTAYQKDFLNAILQVGDIVSGLV--NSR--
----NRK----LQILKDHDGLLRSGEMLLVLGRPGSGVSTLLKTIAGQTK
GLSLDDS--TEF------NYQGIPWD---LMHRKFRGDVT--YQAETDVH
FPHLTVGQTLQYAALARTPHNRLP-----GVSRETYATHLRDVVMAIFGI
SHTVNTKVGDDFIRGVSGGERKRVSIAELALTQSCIQCWDNSTRGLDSAT
ALEFVRTVRLSVDVAGTAAVVALYQASQQAYDVFDKVALLYEGRQIYFGP
IDQAKSYFTE-LGYEC-PERQTTADFLTSLTNPVERVV---RSG-FES--
RVPRTPGEFAKCWEQSVLRARLLGEISDFEREH-----------------
PI--GGPMLQKFESSRNAERSPLMTSNSP--YTISVLQQIALCMRRGYRR
ILGDPSFFIVTVLGNFILSLILGSVFYHL----SDTSVSFTDRCILLFFA
LLFNALNSAL-EILALYAQR-PIVEKHASYAFYHPMSEAMA-SMICDLPC
KILSTLAFNLPLYYMSNLRRDSGHVVIYLLFAFLSTLTMSMIFRTIAQLT
RTVAQALTPIALGVVGLIVYTGFVLPTRNMQ---VWLCWLNYINPIAYSY
ETLVANEFHHREFVC--ASFVP----SGP-G-YESISDTERTCSVA-GAT
SASSVVSGDA-YVEANYGYYYSHTWRNFGILVAFILFFMTTYLLIAEFVK
FSYSKGEVLVFQRKHRVAHIGGEPADDEESTVKKETAASHNCVDSNEGAE
EDQSLK------------------------------------------FR
```

FESNTLHWRDVCY---DVPIKG-EMRRIADHIDGWVTPGTLTALMGASGA
GKTTLLDLLASRVKTGVV----S-GNICVNGTPRDA--SFQRRVGYVQQQ
DVH-LETSTIREALQFSALLRQPASTSRAEKLQYVEEVIDLLEMRSYADA
VVGV--PGEGLNVEQRKRLTIGVELAAKPDLLLFLDEPTSGLDSQTAWSI
SLLLRKL-SNHGQAILCTIHQPSAILFQQFDRLLLLAKGGRTVYFGPIGP
NSKTLIGYFEQHGARPCADEENPAEWMLEVIGAAPG--------------
-------SS-SV-RDWPVTWKESREFQETRKELGRLEQSGS-PS------
---LEDESTSVQQYAAPFYIQLGLCTKRVFEQYWRSPSYIYAKLILCFGA
ALFIGL-SFLN--TKVTVLGLQHQTFAIFMLLVIFAFLAYQTMPNFIKQR
DLYEVRERPAKTYAWSAFMLANIVVDIPWNSLAAVLIFLPFYYIIGMYHN
AEETHTVNERSGLMFLLVWSFMMHCGTFTIMVVASVATAEVGATLALLLF
SM-SLIFCGVMASPASLPGFW-IFMYRVSPMTYLVSGMLSAGLANTAVHC
SDLELVVVQP--PAN-ETCANYLADYME-----------IAGGAVYNPQA
----TADCEYCQMTN--SNVY--LASLSTS-YAERWRNFGLMWAYIAFNI
FAALFLYWFVRVRSGSATSVFRRFA--------KTFSRSSQ---------
-----------RT-------------------------------------

>B6GXJ0

----------------------------MALPVLPSLGFSSATLMPDQME
NMPDNGAVDTPGSETPVSESGDDRDRQITELARSISHVSQKGSVKKSETS
GVNTF---------LDSTSDPELDPNSGLFKPQKWVQNIL-QITSRDPDQ
FPTRTAGVSFRNLNVFGYGTAADYQMNFANFWLKGAGWFRRIL-GLQK--
----KVR----IDILRDFEGIVHSGEMLVVLGRPGSGCSTFLKTIAGETH
GLYLDKEKGSEV------HYDGISWD---VMHSRFRGEVI--YQAENEVH
FPQLTVGDTLLFAAHARAPETRLP-----GVTRDQYAIHMRDVVMTMLGL
THTMNTKVGNEFIRGVSGGERKRVSIAETTLCRCPLQCWDNSTRGLDSST
ALEFVKSIRLSTDYSGSTAIVAIYQASQSIYDQFDKAIVLYEGRQIYFGR
AGDARRFFVE-MGFHC-PDRQTTADFLTSLTSPSERLV---RPG-YED--
SVPRTPDEFAARWKDSPERKQLLAEIEVNAAGD-----------------
----GKAKLQEFDRSRAADKSKLTRAASP--YTLSYPMQIRLCLWRGFLR
LKADSAMTVATIVGNNTMALIISSIFYEL----AYRTDSFYMRGALLFFS
IMISAFSSSL-EIMIMWQQR-PIVEKHFKYALYHPSAEAIS-AYIVELPW
KALLGVTFNLIIYFLPHLRRTAGHFFIFFLFSMTTTLVMSNIFRFIGAIS
RSVAQAMPPASVFMLILVIYTGFTIPVRDMH---PWFRWLNYVNPIAYAF
EALMINEFSGRSFPC--SNYVP----DGS-KIYEDAPLSSKICSQK-GAV
AGQDFIDGET-YINTTYEYYSPHLWRNFGILCAFFVVFFVLYIFCSELIR
AKPSKGEVLVFPRGKMPAFVKNVRKEDPEEVIASEKG-------AVASEP
GDSTAA--IV
RQTSVFHWENVCY---DIKIKG-TKRRILDSVDGWVKPGTLTALMGVTGA
GKTSLLDVLADRVTIGVV----S-GEMLIDGRLRDD--SFQRKTGYVQQQ
DLH-LETSTVREALVFSALLRQPATISRQEKVAYVEEVIHMLGMEEYANA
VVGV--VGEGLNVEQRKRLTIGVELAAKPDLLLFFDEPTSGLDSQTAWSI
CTLMRNL-ADHGQAVLCTIHQPSAMLMQQFDRLLFLAKGGRTVYFGDLGP
NMQTLIKYFEDKGSPKCPPNANPAEWMLEVIGAAPG--------------

-------SH-AD-RDWAEQWTNSAERAEVHSELAEMKKELSKKP------
---VPVRAAGYGEFAMPIWYQFLVCSQRMFQQYWRSPSYLYAKVLTCTVS
PLFLGF-TFWR--MSTSLQGMQNQMFAIFMLLVLFPGLVQQMMPSFVTQR
ALYEVRERPSKAYSWKAFMLGSILVELVWNILMSVPAFLCWYYPIGFYHN
AERTNAVVKRSGIMYVLILQFMMFTSTFSSMVIAGIEEPDTGSNIAQFMF
SL-CLVFNGVLANSSDMPRFW-IFMNRVSPFTYFVSSVLSTGLSGTTVEC
SSIEWLTVSP--PDG-QTCGSYLDPYIE-----------VMHGTLLNHEA
----TVDCKICPMSS--TDQF--LGSLNMS-YSDVPRNIGLMFAYVGFNI
VAALVLYWLFRVPKHWSRKVKET---------------------------

>Q09GQ4

------------------METISVGKESDVSSDGNVAYGGFNEGTNKQIR
DLAREFTRQTSVGDGVQDSDDATDSNEVEKFGVDHASPNSPPKSNQYDLL
RTLTSMSQVPGVNPVDQTIDPRLDPNSDEFESKFWVKNMR-KLLDSDPDY
YRPTSLGFAAKNLIAKGISSDADYQANFLNYPFKVVRDTYMDLFRGND--
---ESRY----FEILKSMDVLIKPGTLTVVLGRPGAGCSTFLKTVAAQTY
GFKVDDS--SII------SYDGLTPK---EINKNYRGEVI--FSAEMDNH
FPHLSVGQTLEFAAKMRTPQNRFP-----GVSRNEYAKHMSEVYMATYGL
SHTVNTKVGDNFIRGVSGGERKRVSIAEASLCGANLQCWDNATRGLDAAT
ALEFVRALKTSAHILDTTPLIAIYQCSQDAYDLFDNVVLLYEGYQIYFGP
GDRAKDFFER-MGYEC-PDRQTTADFLTSITSPAERVA---KKG-WEN--
KVPQTPKEFSDYWRASAEYKELVADIDEYLSHC-----------------
H---NNNTREEFAEAHAIKQANHARPSSS--FRVSYWMQIKLIAQRNIWR
TKGDPSIMMFSVIANIIMGLIISSLFYNL----SATTGTFYYRSAAMFFA
VLFNAFSSLL-EVMSLFESR-PIVEKHKMFALYHPSADAFA-SIFTELPA
KIATSLGFNLMFYFMVNFRRNPGRFFFYLLMNFMATLVMSHIFRSIGSCF
KTLSESMPPATVFLTAMVIYTGFALPTPSMH---GWSRWINYLDPVAYVF
EALMANEFDGRRFEC--SQFIP----S-----YPNADLANQVCSVV-ASV
PGFSYVNGTD-YIYESYRYKITHKWRNFGIVVGFIIFFLFVYVTLVELNK
GAMQKGEIILFQQSKLREMRKEKKSKQISDIEGGSEKPAGVYDHGNEDSE
DGVNN---LT
VGSDIFHWRDVCY---EVQIKD-ETRRILNHVDGWVKPGTLTALMGASGA
GKTTLLDVLANRVTMGVV----S-GSMFVNGRLRDQ--SFQRSTGYVQQQ
DLH-LQTSTVREALRFSAYLRQSRTISKKEKDEYVESIIDILEMRSYADA
VVGV--AGEGLNVEQRKRLTIGVELAAKPKLLLFLDEPTSGLDSQTAWSV
CQLMRKL-ADHGQAILCTIHQPSALLLKEFDRLLFLAKGGRTVYFGDLGE
NCQTLINYFESHGAHPCPAEANPAEWMLEVIGAAPG--------------
-------SH-AN-QDYHEVWMSSDERRAVQEELHRMETELLQIP------
---VDDSAEAKRSFASSYLIQYICVTKRVLQQYYRTPQYIWSKLFLAGAN
SIFNGF-SFYR--AGTSLQGLQNQMLSIFMLSVMLNTLVQQMLPLYITQR
SIYEVRERPSKTFSWWVFLAAQVTAEFPWNLICGTISYFCWYYPIGLQNN
ASVTHTTAERGALTWLLIVGFFNYASSLGLMCIAGVEQEQNGANISNLLF
TM-CLNFCGILKYP---TGFW-KFMYRANPFTFWIASVLGAGVGDTPLVC

SSKEIVYFAP--PKG-ETCTTYIQPYID-----------EAGGYLVDSER
----EGYCGFCTASN--TNAY--LKSVHVE-YSKRWQNWGIFICFIAINN
IFMFLFYWLARVPKKDNRVKDASAL--------KSDEKATL---------
-----------GPQTTSEA-------------------------------

>O13407

-------------------MSQPVEDPSHDQARNDNAQTTTDTGNASM--

------------PKTNGHDQESSATGISSSPADTLMDKEKQAAPTPEPST
EKAAVPDPAASVASDTAEDEFFDDNDSEQRRRNEMVQQLARTYTSRSNAS
AAADEYGNANPFLIASEDPDSPLNPSGNNFKAYAWAKAIA-GMVAAEGGS
F--RTIGICFQNMNVFGFGAATDFQKTVSNVWLEAANM-LRTAVGMGK--
----TTR----IDILRGFNGVVRNGEMLVVLGPPGSGCSTFLKTIAGETN
GLNVDQS--AYF------NYQGLSAE---EMHKRHRGEAI--YTAEVDVH
FPQLSVGDTLTFAANARAPRRAPP-----GVSKTLFANHIRDVVMAIFGI
SHTINTRVGNEYIRGVSGGERKRVTIAEAALSGAPLQCWDNSTRGLDSAN
AIEFCKTLRVCTRLFQTTACVSIYQAPQSAYDMFDKAVVLYEGYQIYFGP
ADEAKQYFVN-LGFEC-PAR-TTPDFLTSMTAPHERIV---RPG-FEG--
KAPRTPEEFAIAWENSAEYTALQADIEEYKSSH-----------------
PI-N-GPDAEAFRKSRAAQQGRGQRPNSP--YTLSFYQQTKLCLWRGWKR
LLGDPTLTVGALFANTLMALVISSIFFNL----QMTTSSFFQRGALLFFA
CLLNGFAAAL-EILILFAQR-PIVEKHDRYALYHPSAEAVA-SMLCDMPY
KVFNAIVFNLVLYFMANLRREPGAFFFYLLISFATVLAMSMMFRTIASMS
RSLSQAMVPAAAIILILIIFTGFVIPLDYML---PWCRWLNYIDILAYSF
ESLLINEFAGQRYTC--TEFVP----RAEFPGYGDLSGTNRVCQAV-GSV
AGQPFVKGED-YLYSSFRYESANKWRNFGILIAFMIFFCSRTWLRPRMCE
RKKSKGEVLVFRRGQRPAAIKDAKTDPEAGPPKV-GG-AVVAANMTGENA
GF--IQ
RQTSTFGWRDVCY---EVQIKK-ETRRILDHVDGWVKPGTLTALMGVSGA
GKTTLLDCLADRTSMGVI----T-GEMLVDGHQRDA--SFQRKTGYVQQQ
DLH-LQTTTVREALNFSALLRQPAHVPRAEKLAYVDEVIRLLDMQEYADA
VVGV--PGEGLNVEQRKRLTIGVELAAKPPLLLFVDEPTSGLDSQTSWAI
LDLLEKL-TKSGQAILCTIHQPSAMLFQRFDRLLFLAKGGKTVYFGDIGE
NSKIMTDYFERNGGFPCPHDANPAEWMLEVIGASPG--------------
-------TT-SD-IDWHQAWRESPECADVHAELDRLKEQVPNTPP-----
---PTEDKASYREFAAPFHQQIYAVTHRVFQQYWRTPSYIYAKAALCAVT
ALFIGF-VFYD--APNTQQGLQNQMFAIFNILTVFGQLVQQTMPHFVIQR
DLYEVRERPSKVYSWKVFMLSQIIVEIPWNSLMAVIMFFCWYYPVGLERN
AILADQVTERGALAFLYLWGFLIFTSTFTDLMIAGFETAEAGGNIANLFF
SL-CLIFCGVLANPDTMPRFW-IFMYRVSPFTYIVSGLLSVAVANSEVRC
ASNEFLHFDP--LNG--TCAEFMRNYINGTTIPGLGRIPGAGGYLRPDTE
--SSRSNCAFCPIKD--TNIF--LQGAHAN-YNDRWRNFGLIFVYIIFNI
IAALFVYWAVRVPKKKLGGKDAAAGVGAGAGAARASASNEK---------
-----------GKMQREKGEVEGLTTAVLGTSVAGSDAPMTTTTEGEGER
AKRRTSGDEVVR--------------------------------------

>B2VWD9

---------------------MSAGNFASPEIVKTPQHNGSDHHTDTESG
ASTIAAVEGTPREHHLDDRKDGSYNTSEADEIAEIARREEAVHQLARRLT
SQSQQSSGNQNPF--NAALNSALDPHGENFNARAWTRAML-NLQMNDENA
APPRTAGVCFHDLNVYGFGTDTDYQKTVGNIWLDVVGM-VKSFTG-DK--
----GRK----IDILQKCDGLVESGEMLVVLGPPGSGCSTFLKTITGETH
GFFIDEN--SAI------NYQGISPE---LMHHNYRGEAI--YTAEVDVH
FPAMTVGETLYFAALARRPRIIPG-----GVTKEQYAAHQRDVIMALFGI
SHTINTRVGNDFIRGVSGGERKRVTIAEASLSRAPLQAWDNSTRGLDSAN
AIEFCKTLRMETEINGTTACVAIYQAPQGAYDLFDKVLVLYEGRQIYFGK
TSEAKSYFVN-MGFEC-PSRQTDADFLTSMTSALERVV---RPG-FED--
RVPRTPDEFAKRWLESADRAALLQDIAAYEQKY-----------------
PI-G-GEASERFKESRNLQKAKGMREKSP--YTLSYAQQITLCLWRSFVR
LKADPSITIIQLVFNMIMALIIASVFYNL----QETTISFFSRSALLFFA
ILMNAFGSAL-EILTLYAQR-SIVEKHSRYALYHPSAEAFA-SMLMDMPY
KICNALSFNMVLYFMTNLRREPGNFFFFVLLSFTLTLTMSMLFRSMAALS
RSLVQALAPAGILMLGLVMYTGFAIPPSYML---GWSKWMRWINPVAYGF
ESLMVNEFSGRQFRC--NTFVP----SG--PGFESITGLGRACNAI-GSV
PGQPYVSGDA-YINSSFNYHAKNKWRNFGLLWVFMFGLMLVYLTGTEYIT
AKKSKGEVLVFRRGHKLSASKSKPQDDLEAAP---AG-RNVVAQNSSDEI
AI--ID
RQTAIFQWENVCY---DIKIKN-EHRRILDHVDGWVKPGTLTALMGVSGA
GKTTLLDCLATRTTMGVI----T-GEMLVDGKPRDG--SFQRKTGYAQQQ
DLH-LSTTTVREALIFSAVLRQPAHVPRQEKLDYVDEVIKLLEMEEYADA
VVGV--PGEGLNVEQRKRLTIGVELAAKPALLLFLDEPTSGLDSQTSWAI
LDLLDKL-KKNGQAILCTIHQPSSMLFQRFDRLLFLAKGGRTVYYGDVGE
NSQILVDYFVRNGGPPCPPSANPAEWMLEVIGAAPG--------------
-------SH-TE-IDWPQVWRDSPEKAEVKRHLAELKNTLRQTKS-LERT
VSNQERDKAAYREFAAPFLVQMRETLVRVFQQYWRTPTYLYSKLFLCGVS
AAYIGF-SLFQ--MPNTQTGMQNQMFGIFMLLTIFGQMLQQMMPHFVTQR
ALYEVRERPSKAYSWKAFMLSNIIVEIPWNALMAVVIYFCWYYPIGLYKN
AQPTDEVAYRGFQFFLMVLMFLLFTSTFSSMVIAGMETAEAGGNIAQLVF
SL-CLIFCGVLAQPGTFPKFW-IFMYRVSPFTYMVSGLLSSGLANNVVNC
AQNELIRFEP--PQG-TTCGDYMSPYIA-----------TNGGKLS--NP
--NATNDCRFCTIAT--TNKF--LANISSS-YSDYWRNFGILWVYVVFNI
IMALVLYWLVRMPKPKKEEETPVGNGAGELDGPSHSASGAQ---------
-----------AEKNDNDPARY--------ASITGTHTPVSKELTEKL--

>Q1E7P5

--MA--

------------NAPPPFGVSALAADTGANFQDTVSSQTEVNGSEFQAKA
YGDETKPSTASSVTAGKISELDDGDTGITKDQEHEIVDLARRVSRLSRRS
SVYDHDNVVNPFL--DAEANPELNPGDKQFKPRKWLKTIL-QIQSRDPER
FPKRTAGVSFRNMNVYGYGTAADYQADVGNTPLKAVGG-IKQLLGLGK--
----KVR----IDILRNFEGLVKSGEMLVVLGRPGSGCSTFLKTLAGETH
GLYLDEG--NDI------QYQGVSWN---QMHKNFRGEVI--YQAETETH
FPQMTAGDTLYFAARARAPVNRLP-----GVTRQQYAEHMRDVVMSMLSL
SHTMNTKVGNEYIRGVSGGERKRISIAETTLSGSPLQCWDNSTRGLDSST
ALEFVKSLRLSTQYSGTTAIVAIYQAGQAIYDIFDKAIVLYEGRQIYFGN
AVRAKHYFIE-MGFEC-PDRQTTADFLTSITNPAERRI---RPG-FQN--
RVPQTPDEFAERWRKSPERQALLAEIEEYNAEF-----------------
PL-D-GAQLEKFQQSRQAEKSKAVRKSSP--YTLSFWQQVQLCIWRGFVR
LKGDMSMTLTSVIGNIVLSLIISSVFYNQ----PDNTSSFFGRGSLLFFA
ILMNGFASSL-EILTLWHQR-PIVEKHDKYALYHPSAEAIS-SMIVDLPS
KAIVAIVFNLILYFMVNLRRTPGHFFVFFLFSISTTLTMSNIFRTIGAIS
RTLAQALVPSSIFMLALVIYTGFTIPVRDMR---PWFKWISYLNPIQYAF
ESLMINEFHDRRFPC--AMFVP----MG--PSYANATGDSIICGAT-GAR
PGEDFVDGDT-FLNITYGYYASHLWRNLGIIFAFFFFFLVTYIGATELIK
AKPSKGEILVFPRGKVPSYLKSKKNSDDPESAETVNQ-KQKLESTGHDQV
GA--IV
KQTSIFHWQDVCY---DIKIKN-QPRRILNNIDGWVKPGTLTALMGVSGA
GKTTLLDVLADRVTMGVV----T-GEMLVDGRIRDD--SFQRKTGYVQQQ
DLH-LEISTVREALVFSALLRQPATTPRAEKIAYVEEVIKMLGMEEYADA
VVGV--LGEGLNVEQRKRLTIGVEIAAKPDLLLFFDEPTSGLDSQTAWSI
CTLMRKL-ADHGQAILCTIHQPSAMLMQEFDRLLFLAAGGKTVYFGELGK
NMSTLIDYFENHGSPKCPPNANPAEWMLEVIGAAPG--------------
-------SH-TD-KDWSQVWNNSPERAEVRRQLAEMKAELSEKPQ-----
----APRSAGYGEFAMPFWQQYLVVQHRMFQQYWRSPEYIYSKLCLCIVP
TLFIGF-TFYK--EPTSLQGLQNQMFAIFMFLILFPNLVQQMMPYFVTQR
SLYEVRERPSKAYSWKAFMLSSVLVELPWNTLMAVPAFFCWYYPIGFHRN
AIASDAVTERSGTMFLLVWIFLMFSSTFSSMIIAGVELAETGGNLAQLLF
SL-TLIFCGVLATPQAMPRFW-IFMYRLSPFTYYVSAVLSTGVANTNVNC
SAREFLRLIP--PAG-QTCGQYLHDYML-----------LAGGALL--NP
--ESTSACDFCPVKD--TNTF--LEQVNIR-YSERWRNIGILFVYIFVNV
IGAIGFYWLLRVPKTGLFKKKAKKD-------------------------

>Q96VL9

--------------------------------MATSAPFGVLPQSEAVAE
KVEEVVQEKTYQNIDHKRKDSDSESSTLDSTREREDATIRTLARQITQKS

MEGAYDHNINPFL--GSN-DPRLDPSSEKFSSRAWTKHLV-HLQSRDPER
YPNRTAGIAYKNLNAHGFGEPTDYQKTFGNYPLEIIGM-SKRVLGLSK--
----PTK----IQILRDFDGLIRSGEMLVVLGRPGSGCSTLLKTISGETS
GFFVDSN--TYI------NYQGIPME---TMHNDFRGECI--YQAEVDVH
FPQLTVAQTLGFAAKAKAPRNRIP-----GVTRDQYAEHLRDVTMATFGL
SHTFNTKVGNDFIRGVSGGERKRVSIAEAAVGGSPLQCWDNSTRGLDSAT
ALEFVKTLRNSTELSGSTAVVAIYQASQSIYDLFDKVAVLYEGRQIYFGD
INAAKTFFVN-LGFDC-PARQTTADFLTSITSPAERIV---RPG-FEG--
RTPYTPDEFAAVWQKSEDRAQLLREIDQFDAEF-----------------
PI-G-GQALDDFKNSRKAVQAKGQRIKSP--YTISLPMQIRLCVERGFQR
LRGDMSLLLTGLIGQSVMALIIGSVFYNL----ADDTNSLYSRGALLFFS
ILMAAFQSAL-EILTLYAQR-PIVEKHTKYAFYHPVAEACA-SMLCDIPN
KVFSTIFFDLALYFMTNLRREPGYFFVFFLFTFLCTLTMSMYFRSIASLS
RSLSEAMAPAAIFILAIVTYTGFAVPIRDMH---PWFRWINYLDPVSYGF
EALMINEFHGRKIPC--SVFVP----SG--GNYGNVGADERICSTT-GAA
AGADYVDGDR-YLEVNYGYNHSHLWRNLGVMIAFMFLGLFIYLSASEFIS
AKKSKGEVLLFRRGRIPYVSKASDEEAKIDDRMT-AA-TVTRTKTVPDAP
PS--IQ
KQTAIFHWDDVHY---DIKIKG-EPRKLLDGVDGWVKPGTLTALMGVSGA
GKTTLLDVLASRVTMGVV----T-GQMLVDGRQRDI--GFQRKTGYVQQQ
DLH-LATSTVREALAFSAILRQPKATPHAEKIAYVDEVIKVLEMEEYADA
IVGV--PGEGLNVEQRKRLTIGVELAAKPALLLFLDEPTSGLDSQTAWSI
CALLRKL-ADNGQAILCTIHQPSAILFQEFDRLLFLARGGRTVYFGEIGE
HSKVLTNYFERNGAHPCGDLANPAEWMLEVIGASPG--------------
-------AS-NT-IDWPETWKNSPERQQVKSHLAELKTTLSQKQV-----
----EHDPTSLNSFAAGFGTQMQVVLVRVFQQYWRTPPYLYSKTALCLCV
GLFLGF-SFYD--TKTSLQGMQNQLFAIFMLFTIFGNLVQQILPHFVTQR
SLYEVRERPSKTYSWKVFILSNIIVELPWNTLMAVIIFVTWYYPIGLYRN
AEMTNAVNERSGLMFAFIWMFLMFTSTFADFIIAGIDTAENAGNIANLMF
SL-CLIFCGVLASPTALPGFW-IFMYRVSPFTYLVSGMMSTGLANTEVVC
DSIEYLHFNP--PSA-KTCGEYLGPYMS-----------AAGGYLN--NP
--EAMTDCEFCSIKD--TNVF--LAGVNSY-YSDVWRNWGLMWVYLAFNI
AGAIGMYWLVRVPKGASQGEDNEEGAKKGWFSKKKADA------------

>Q0CEM0

----------------------------MGLQPAPPVPFGASAVAASAGL
EFENAIQQHIHRNGEGAGSTDTTGRDSEDGIGPEGQGKIAALARTFSQMS
QKSALGEGTNTFL--NPNHDPELDPQSDKFSSRKWTKNLL-HITSRDPDR
YPRRTTGVSFRNLNAFGYGTAADYQADVANMWLKGFGW-LRGALGFGN--
----KVR----IDILRNFEGFVKSGEMLVVLGRPGSGCSTFLKTIAGETH
GLWLDQG--TDI------EYQGISWD---EMHSRYRGEVI--YQAETEIH
FPQLTAGDTLLFAAHARAPANRFP-----GVTREQYATHMRDVVMAMLGL
SHTMNTKVGNEFIRGVSGGERKRVSIAETMLCGSPLQCWDNSTRGLDSST

ALEFVKCLRLSTEYTGSTAIVAIYQASQAIYDCFDKAIVLYEGRQIYFGS
ASDARRFFIE-MGFDC-PDRQTTADFLTSLTSPTERRA---RKG-FEH--
LVPRTPDEFAERWQQSAERKQLLADIKAFRNEF-----------------
PI-G-GNKQEEFSRSRAAEKAKATRAASP--YTLSYPMQVRLCLHRGFLR
LKGDMSMTLASVIGNSIMSLVIASVFYNL----DGTTNSYFSRGALLFFS
ILLNAFASAL-EILTLWQQR-PIVEKHDKYALYHPSAEAIS-ALIVDLPS
KALVSVAFNLILYFMTNLRRTPGHFFVFYLFSVTTTLTMSNIFRWIGAIS
RSLAQAMVPSSIFMMILVIYTGFTIPVKNMH---PWFRWLNYLNPIAYAF
ESLMINEFSGRDFPC--AQYMP----SG--PGYENVPMSSKVCVGN-GAV
AGQDHINGDA-YINTSYQYYKEHLWRNYGIIVAFFFFFLFAYVICSELIR
AKPSKGEILVFPRGKIPTFAKKAAAPGDLETAPT-SE-KQSLDTGSSDHT
AS--LA
KQTAIFHWQDVCY---DIKIKG-ETRRILDHVDGWVKPGTLTALMGVTGA
GKTSLLDVLANRITMGVI----T-GDMLVDGRPRDD--SFQRKTGYVQQQ
DLH-LETSTVREALIFSAILRQPSSVPRKEKLAYVEEVIKMLNMEEYAEA
VVGV--LGEGLNVEQRKRLTIGVEIAAKPDLLLFFDEPTSGLDSQTAWSI
CSLMRKL-ADHGQAVLCTIHQPSAILMQQFDRLLFLAKGGKTVYFGELGE
NMETLIRYFENKGSSKCPPNANPAEWMLEVIGAAPG--------------
-------SH-AD-QDWPEVWNLSPERMEVRRELATMREELSKKPL-----
----PPRTKEYGEFAMPLWTQFLICLQRMFQQYWRTPSYIYSKAAMCIIP
PLFIGF-TFWR--EPLSLQGMQNQMFSIFMLLIIFPNLVQQMMPYFVTQR
ALYEVRERPSKAYSWKAFMMASILVELPWNILMAVPAYFSWYYPIGLYRN
APPGETV-DRGGTMFLLILIFMMFASTFSSMIIAGIEHPDTGSNIAQMMF
SL-CLIFNGVLASPKALPGFW-IFMYRVSPFTYLVSAVLSVGLAGNEVKC
ADYEILHIPP--PEG-QNCSSFLGPFVQ-----------MAHSTLL--TP
--ESTTDCQMCTLQS--TDQF--LAQLSMS-FDDRWRNVGLLFIYVVFNA
VAAVFLYWLIRVPKKSSVKEKKD---------------------------

>Q0D099

------------------MSLLGTINPNLNPSRSVGGRGQGDDDDDHNNR
RHHLGGHPQSSTLSTAETALEKESEYGDDSSEEEVVEQITRLAQQLTRES
TRFSTSDNALNSFL-GVDEDSTLNPHGPNFKARHWMKNLL-ALSSRDPEL
YPRREAGVAFKGVSVHGFGSPTDYQKDVFNAVLQIGAL-FRMATG-TG--
----KQK----IQILRDFDGLVRSGEMLVVLGRPGSGCTTFLKTLAGEMN
GIYMDES--SHM------NYQGISPK---QMMTQFRGEAI--YTAETDVH
FPQLSVGDTLKFAALARCPRNRFP-----GVTKEQYALHMRDAVMAMLGL
SHTINTRVGNDFVRGVSGGERKRVSIAEATLSGSPLQCWDNSTRGLDSAN
ALEFCKTLNLMTKYAGATVAVAIYQASQSAYDVFDKVTVLYEGRQIYFGR
TDEAKQFFTD-MGFEC-PDRQTTADFLTSLTSPSERIV---KKG-YED--
RVPRTPDEFAAAWKNSEAHAKLIREIDEYNQEY-----------------
PL-G-GEALGKFIESRKAMQAKSQRVGSP--YTVSVYEQVNLCMVRGFQR
LKGDASLTISQLIGNFIMALIIGSVFYQM----KDDTSSFYSRGALLFFA
VLLNAFSSAL-EILTLYAQR-PIVEKQSRYAMYHPFAEAIA-SMLCDMPY

KIGNAIIFNITLYFMTGLRQTPGAFFTFLLFSFVTTLTMSMLFRTIASSS
RTLSQALVPAAILILGLVIYTGFTIPTKNML---GWSRWMNYINPIAYGF
ESLMVNEFHNRRFPCAQSGFVP----SGAELGYANVPLANKICSTV-GAV
AGSQFLEGDD-YLHQSFAYYNNHKWRNLGIMFAFMIFFMVTHLATTEYIS
EAKSKGEVLLFRRGQAPPAESNDIEMTSNIG----AT-AKTNESPEGAA-
--IQ
RQEAIFQWQDVCY---DIKIKG-EPRRILDHVDGWVKPGTCTALMGVSGA
GKTTLLDVLATRVTMGVV----T-GEMLVDGRPRDQ--SFQRKTGYVQQQ
DLH-LHTTTVREALRFSAILRQPAHVSRQEKLDYVEEVIKLLGMEAYADA
VVGV--PGEGLNVEQRKRLTIGVELAAKPQLLLFLDEPTSGLDSQTSWSI
LDLIDTL-TKHGQAILCTIHQPSAMLFQRFDRLLFLAKGGKTVYFGEIGD
KSSTLSSYFERNGAPKLPADANPAEWMLEVIGAAPG--------------
-------SH-SD-IDWPAVWRESPERAAVREHLAELKSTLSQKPV-----
-QQSQNDPNSFNEFAAPFTVQLWECLVRVFSQYWRTPVYIYSKACLCILT
AMYIGF-SFFH--AHNSQQGLQNQMFSIFMLLTIFGNLVQQIMPNFCTQR
SLYEARERPSKTYSWQAFMTANIMVELPWNTLMSVLIYVCWYYPIGLYRN
AEKTNAVSERGALMWLLIWSFLMFTSTFAHMMIAGIELAETGGNLANLLF
SL-CLIFCGVLATPEVLPGFW-IFMYRVSPFTYLVSGMLSTGVSGADAIC
ENYEFLRFPP--PAN-QTCGDYMSNYIA-----------AKGGYLQ--DP
--GSTTDCNFCSISN--TDTF--LAAVHSD-FKDAWRNFGLMWVYIVFNV
FAAVGIYWLARVPKGSRTKN------------------------------

>Q6MY55

------------------MSLLGTINPNINPERTVAGRGTQEEEGEIARV
E-----HNHHNNAASVSTDETVLERSKEIGDEDVAVEEVTRLARQLTRQS
TRFSTSGNVENPFL-ETKEDSTLNPLSPNFKAKNWMKNLL-ALSSRDPER
YPKRVAGVAFKNLSVHGYGSPTDYQKDVFNSVLEVGTL-VRRIMG-TG--
----KQK----IQILRDFDGLVKSGEMLVVLGRPGSGCSTFLKTISGEMN
GIYMDEK--SYL------NYQGISSK---QMRKQFRGEAI--YTAETDVH
FPQLTVGDTLKFAALARAPRNRLP-----GVSREQYAVHMRDVVMAMLGL
THTMNTRVGNDFVRGVSGGERKRVSIAEATLSGSPLQCWDNSTRGLDSAN
ALEFCKTLNLMTKYAGATVAVAIYQASQSAYDVFDKVTVLYEGRQIYFGR
TDEAKEFFTN-MGFEC-PERQTTADFLTSLTSPAERVV---KPG-FEG--
KVPQTPDEFVRAWKSSEAYAKLMREIEEYDREF-----------------
PI-G-GESLNQFIESRRAMQAKNQRVKSP--YTISVWQQIELCMIRGFQR
LKGDSSLTMSQLIGNFIMALVIGSVFYNL----PDDTSSFYARGALLFFA
VLLNAFSSAL-EILTLYAQR-PIVEKQARYAMYHPFAEAIA-SMLCDMPY
KITNAIIFNLTLYFMTNLRREPGAFFVFLLFSFVTTLTMSMLFRTMAASS
RTLSQALVPAAILILGLVIYTGFTIPTRNML---GWSRWMNYIDPIAYGF
ESLMVNEFHNRQFLCPDSAFVP----SSG--AYDSQPLAYRVCSTV-GSV
SGSRYVQGDD-YLNQSFQYYKSHQWRNLGIMFGFMFFFMFTYLTATEYIS
ESKSKGEVLLFRRGHAQPTGSHDVEKSPEVS----SA-AKTDEASSKEAT
GA--IQ

RQEAIFQWKDVCY---DIKIKG-EPRRILDHVDGWVKPGTCTALMGVSGA
GKTTLLDVLATRVTMGVV----S-GEMLVDGRPRDQ--SFQRKTGYVQQQ
DLH-LHTTTVREALRFSALLRQPAHVPRQEKIDYVEEVIKLLGMESYADA
VVGV--PGEGLNVEQRKRLTIGVELAAKPQLLLFLDEPTSGLDSQTSWSI
LDLIDTL-TKHGQAILCTIHQPSAMLFQRFDRLLFLAKGGKTVYFGEIGE
KSSTLASYFERNGAPKLPPDANPAEWMLEVIGAAPG--------------
-------SH-SD-IDWPAVWRDSPERRAVHEHLDELKRTLSQKPI-----
-DPSKADPGSYDEFAAPFTIQLWECLLRVFSQYWRTPVYIYSKTALCVLT
ALYIGF-SFFN--AQNSAQGLQNQMFSIFMLMTIFGNLVQQIMPNFCTQR
SLYEVRERPSKTYSWKAFMAANIIVELPWNTLMAFLIFVCWYYPIGLYRN
AEPTDSVHERGALMFLLIWSFLLFTSTFAHMMIAGIELAETGGNLANLLF
SL-CLIFCGVLAPPQSLPGFW-IFMYRVSPFTYLVSAMLSTGVSGTNAVC
EPVEFLHFDP--PSN-MTCKDYMADYIS-----------TRGGYLE--NP
--SATSDCTFCTISS--TDTF--LSAVSSH-YSDAWRNFGIMWAYIIFNI
FAAVFIYWLARVPKGKRTKGST----------------------------

>Q2U0M6

------------------MSLLGTINPNLNPARAVGAQGDAEGAAPVSGG
EPM-PIFEGNDSARTSDTALDKLGKDESKYDEQIAEAEVTRLAQQLTRQS
TRFSVSQNAENPFL-ETKEDSTLNPLGPNFKAKNWMKNLL-ALTSRDPER
HPRREAGVSFRNLSVHGYGSPTDYQKDVFNMVLQVGAL-FRAVTG-TG--
----KQK----IQILRDFDGLVKSGEMLVVLGRPGSGCSTFLKTLAGEMN
GIYMDDK--SDL------NYQGIPAK---QMRRQFRGEAI--YNAETDVH
FPQLSVGDTLKFAALTRCPRNRFP-----GVSREQYATHMRDVVMAMLGL
THTINTRVGNDFVRGVSGGERKRVSIAEATLSGSPLQCWDNSTRGLDSAN
ALEFCKTLNLMTKYAGATVAVAIYQASQSAYDVFDKVTVLYEGRQIYFGR
TDEAKEFFTT-MGFEC-PERQTTADFLTSLTSPSERIV---KKG-YEG--
KVPRTPDEFAAAWKNSEAYAKLIREIEEYNREF-----------------
PL-G-GESVQKFVESRRAMQAKNQRVGSP--YTVSIYEQVRLCMIRGFQR
LKGDSSLTMSQLIGNFIMALIIGSVFYNL----QHDTSSFYSRGALLFFA
VLLNAFSSAL-EILTLYAQR-PIVEKQARYAMYHPFAEAIA-SMLCDMPY
KITNAIIFNITLYFMTNLRREPGPFFVFLLFTFVTTMTMSMLFRTIAASS
RTLSQALVPAAILILGLVIYTGFTIPTRNML---GWSRWMNYLDPIAYGF
ESLMVNEFHNTKWKCSSAELIP----N-----YEGASLANKICSTV-GAV
AGSEYVYGDD-YLEQSFQYYESHKWRNLGIMFAFMVFFLATYLTATEYIS
EAKSKGEVLLFRRGHYSRG-AADVETHNEVS----AT-EKTNESSDGAGA
-A--IQ
RQEAIFHWQDVCY---DIKIKG-EPRRILDHVDGWVKPGTCTALMGVSGA
GKTTLLDVLATRVTMGVV----T-GEMLVDGRLRDQ--SFQRKTGYVQQQ
DLH-LHTTTVREALRFSAILRQPAHVSRQEKLDYVEEVIKLLGMEAYADA
VVGV--PGEGLNVEQRKRLTIGVELAAKPQLLLFLDEPTSGLDSQTSWSI
LDLIDTL-TKHGQAILCTIHQPSAMLFQRFDRLLFLAKGGKTVYFGEIGE
RSSTLASYFERNGAPKLPVEANPAEWMLEVIGAAPG--------------

```
-------SH-SD-IDWPAVWRESPEREAVRNHLAELKSTLSQKSV-----
-DSSHSDESSFKEFAAPFSVQLYECLVRVFSQYWRTPVYIYSKAVLCILT
SLYIGF-SFFH--AENSRQGLQNQMFSIFMLMTIFGNLVQQIMPNFVTQR
ALYEARERPSKAYSWKAFMTANILVELPWNALMSVIIFVCWYYPIGLYRN
AEPTDSVHERGALMWLLILSFLLFTSTFAHMMIAGIELAETGGNLANLLF
SL-CLIFCGVLATPETLPGFW-IFMYRVSPFTYLVSGMLATGVGRTTAVC
EKVEFLHLTP--PAN-TTCYDYMSDYIG-----------SFGGYLE--ND
--NATDSCSFCQISS--TDTF--LSAVSSY-YEDRWRNFGIMWAFIVFNI
AAAVFIYWLARVPKGSRSKN------------------------------
--------------------------------------------------
--------------------------------------------------
--------------------------------------------------
-----------------------------
>Q5BFA9
--------------------------------------------------
--------------------------------------------------
--------------------------------------------------
--------------------------------------------------
--------------------------------------------------
--------------------------------------------------
--------------------------------------------MSSF--
--------------------------------------------------
------------LGTGTFNTSVSPSQAVESRGIENHGNAITETETLHNES
HAESPGEKLASSSNSILSSTETAREKDERDYELDAEEEVTRLAQQLTHQS
TKYSTH-NIENPFL-EVGEDSTLNPHSPNFKAKNWMKNLL-ALSSRDPER
YLPRQAGVSFTNLSVHGYGSPTDYQKDVFNSVLQIGGL-VRSMMG-HG--
----KQK----IEILRNFDGLVKAGEMLVVLGRPGSGCSTFLKTIAGEMN
GIFMDEK--SQL------NYQGIPAK---QMRKQFRGEAI--YTAETDVH
FPQLSVGDTLKFAALARCPRNRLP-----GVSREQYAVHMRDVVMAMLGL
SHTINTRVGNDFVRGVSGGERKRVSIAEATLSASPLQCWDNSTRGLDSAN
ALEFCRTLNLMAKYSGATMAVAIYQASQSAYDVFDKVTVLYEGRQIYFGR
TDDAKQFFID-MGFEC-PERQTTADFLTSLTSPAERIV---RKG-YEG--
RVPQTPDEFAAAWKNSDAYAQLMREIEEYNQEF-----------------
PL-G-GESVNKFIESRRAMQSKNQRVKSP--YTMSVMEQVNLCMIRGFQR
LKGDASLTLSQLIGNFIMALVIGSVFYDL----DNDTGSFYSRGALLFFA
VLLNAFGSAL-EILTLYAQR-PIVEKQARYAMYHPFAEAIA-SMLCDMPY
KITNTFTFNIPLYFMTNLRREPGAFFIFLLFSFVTTLTMSMLFRTMAATS
RTLSQALVPAAILILGLVIYTGFTIPTRNML---GWSRWMNYIDPIAYGF
ESLMVNEFHGRLFPCSESELVP----S-----YGDT--ANRVCAVV-GAT
PGELMVNGTT-YLRESYQYTKSHEWRNLGIMFAFMAFFLFTYLTATEYIS
EAKSKGEVLLFRRGQAPPS-VNDVETHSPAT----AG-EKVDQSTQDVAN
------------------------------------------------IQ
RQTAIFHWKDVCY---DIKIKN-EPRRILDHVDGWVKPGTCTALMGVSGA
GKTTLLDVLATRVTMGVV----T-GEMLVDGRPRDQ--SFQRKTGYVQQQ
DLH-LHTTTVREALRFSALLRQPAKTPRQEKLDYVEEVIKLLGMEAYADA
VVGV--PGEGLNVEQRKRLTIGVELAAKPQLLLFLDEPTSGLDSQTSWSI
LDLIDTL-TQHGQAILCTIHQPSAMLFQRFDRLLFLAKGGKTVYFGEIGE
KSSTLASYFERNGAPKLPADANPAEWMLEVIGAAPG--------------
-------SH-SD-IDWPAVWRESPERQAVHQHLAELKETLSQKPT-----
-ETSASDPSEYNEFAAPFSVQLWECLVRVFSQYWRSPVYIYSKAALSILT
SLYIGF-SFFQ--AQNTRQGLQNQMFSIFMLMTIFGNLVQQIMPNFVTQR
ALYEVRERPSKAYSWKAFMTANILVELPWNTLMAVIMYFCWYYPVGLYRN
AEPTDSVHERGALMFLLILAFLLFTSTFAHMIIAGIETAETGGNIAQLLF
SL-CLIFCGVLAGPDVLPGFW-IFMYRVSPFTYLVSAMLSTGVSGTTAYC
```

EQVEYLTLYP--PSN-TTCSEYMDPYIS-----------QVGGYLQ--NP
--DATSECTFCQISS--TDTF--LSAVYSN-YDDAWRNFGLMWAYIAFNI
AAAVFIYWLARVPKGKKN--------------------------------

>Q3ZZY6

--MA--

------------STYMGGTSVQPAKLPGLGPNLTANNQTTTSTHDPQTPV
NPSSDTTPTNYSQDHMASKDETPAKSTTEDEEDSELERRHSIVRDLARQY
TNQSQMSAISGNPF-TADENSPLNPRSDKFRALAWAKAIS-KLHSGTG--
FINRKAGVCYQNLNVFGYGQPTDYQKNVANIWLDVASL-PRQLMG-YG--
----KTR----IDIIRDFDGVVKNGEMLVVLGPPGSGCSTYLKTISGETS
GIYINDD--AYF------NYRGITAH---EMHTRHRGEAI--YTAEVDVH
FPHLTVGDTLDFAAHARAPRSIPG-----GIDRETFISHSRDVVMAMFGI
SHTVNTKVGNEYVRGVSGGERKRVTIAEASLSGAPLQCWDNSTRGLDSAN
AVEFCKTLRLQTQMSDTAAMVSIYQAPQAAYDIFDKVVVLYEGRQIFFGG
TKAAKRYFED-LGFEC-PARQTVPDFLTSMTSPQERII---RKG-FED--
RAPRTPDEFAAAWKASQANILLQQEIEEYKRDF-----------------
PI-N-GPEAEEFRANRRAAQAKNQRKKSP--YTLSYWQQTKLCVWRGFKR
LVTDPTITLTQLFGNFIMALIVGSVFYNI----NQTTSSFFQRGALLFLA
CLSNAFSSAL-EILTLYAQR-PIVEKHDRYALYHPSSEAVA-SMLCDMPY
KL-NAFTYNLTLYFMTNLRREPGPFFFFLFMTFLVTLCMSMIFRTIASAS
RTLSQAMVPAALLILALVTFTGFVIQIDYML---GWCRWINYVNPLAYAF
ESLMVNEFHNRKFEC--NLFIP----T-----YPDAAPENRVCSTV-GSV
QGEPMVSGDR-YINLSFSYYHAHKWRNVGIVIAFTLLFLVTYMFFAEAVS
AKKSKGEVLVFRRGHRLAKNKADAESSPAGR----VA-ITEKEGYGEGQP
-S--NF
KSTSVFHWNNVCY---DVKIKS-ENRRILDNVAGWVKPGTMTALMGVSGA
GKTTLLDCLADRTSMGVI----H-GDILVDDKLRDA--SFQRKTGYVQQQ
DLH-LSTTTVREALNFSAIMRQPKHIPVKQKIAYVDEVIKMLDMQDYSEA
VVGI--LGEGLNVEQRKRLTIGVELAAKPPVLLFVDEPTSGLDSQTSWAI
LDLLEKL-ARSGQAILCTIHQPSAMLFQRFDRLLFLASGGKTVYFGDVGD
HSRTMTRYFESHGAEPCPPEANPAEWMLEVIGAAPG--------------
-------SE-TN-LDWPQIWKDSSEFAEVQKHLKELSHHQVEKSQ-----
-D---EDPHLYDEFAATFTTQLKYVTARVFEQYWRTPSYIYSKAALCTLV
ALFIGF-SFYQ--APNTALGLQNQMFAIFQVLTVFGQLTQQQMPHFVIQR
DLYEVRERPSKTYSWQVFILSQIIVEIPWNTLMAAIMYFCWYYPIGLYRN
AEPTDAVAERGALMFLLFLVFMLFTCTFTDFIIAGCSSAETGGNIANLLF
MM-CLIFCGVLATPDSFPKFW-IWMYRVSPFTYLISAILSTAVANTEVVC
AQNEYVVFPP--PKG-QICGEYMQAYIK-----------RAGGYLV--DE
--SSNSTCTYCTIGD--SNVF--LAGVRSH-YDERWRNFGLMWVYIIFNV
FAALALYWLVRVPKNKKGEKEKKE--------------------------

>B2WML3
MASLNGAPEDTVAPGLVDTSITKEGFKITTRKLPILKAGPIEEMTKKLGI
APPEMIFGDNLVRIENIESGWYIEFNAFDALDRVDKTGEKMFKVSYSKEW
QQNRQKQFEDIKEVVKPFDWSYSTDYKGTTPPTPAFEPTETPIPLALLKR
PDPIQFFD-ELVLYEDELADNGIAMLSCKIRVMPQRLLLLVRFFMRLDDV
VFRIRDTRIFVEFGDKVILREYTAREEKYEDVRKKLAGRKEDVLAIMRDP
NRLAEHIPIVEHTLEGLQLNTQRAYHLAISYKPVRANTQIYSGQKTVRDR
LRLPVLTIILVRDRQALANFGRLSVRNPSEHTYRTLQLAIRSSFTHAFAR
VSQEPGESEGTIPVSPGDFKVLRDSYRRCEMRQFVATHHGRPTLQRRALH
VEHFKFTTKYFPHTHRKALPGQPQAARRTFLMAHEEVIQLAQVSTGSSSN
SSQTHVGQNGFPSKTKGEISSSGSYEAAPGTLQRQPTRPEIDDEGRRELV
RIFSTASPVTRQMSVAQPGDPTVDPSSDAFDLTKFLHMFR-N--QLEGEG
VEMKKLNVVYKNLNVFGSGKALQLQDTVTDLFLAPFRA-KEYF---GK--
----SER----KQILHDFDGIIRSGELCVVLGRPGSGCSTLLKALTGELH
GLD-ADD--SII------HYNGIPQS---RMVKEFKGETV--YNQEVDKH
FPHLTVGQTLEFAAAVRTPSNRPL-----GMSRDEYAKFMARMVMAVLGL
SHTYNTKVGSDFVRGVSGGERKRVSVAEMMLAGSPFASWDNSTRGLDSAT
ALKFVRALRVGADMTGGTCAVAIYQASQSVYDCFDKATVLYEGRQIYFGP
ANEARGYFER-QGWYC-PPRQTTGDFLTAITNPLERQA---RKD-MKD--
QVPRTPEDFEKYWRNSPEYRALLEDIKDFEAEN-----------------
PI-NENGGLQQLRQQKNYTQAKGARPKSP--YLISVPMQIKLNTRRAYHR
IMGDIASTATQVVLNVIIALIVGSIFFGS----SKGSNSFQGRGSAIFLA
ILFNALTSIG-EISGLYAQR-PVVEKHNSYAFYHPATEAIA-GVVMDMPV
KFANAVVFNIILYFLARLRTTPGQFFIFFLVTYIVTFVMVAIFRTTAAVT
KTASQAMAGAGVLILVLVVYTGFVVRIPSMP---KWFGWMRWINPIFYAF
EILMANEFHGVEFPC--DRTIP----SG--AGYTQ-DGGNFICDAQ-GAI
AGQNFVSGDR-FIAAAYQYTWSHVWRNFGILCAFLIFFMVTYFVAVEVNS
STTNTAEQLVFRRGHVPAHLQSGDKASDEESGET-----RQGGQDAPGDI
SA--IE
EQKGIFTWRDVVY---DIEIKG-EPRRLLDHVSGFVKPGTMTALMGVSGA
GKTTLLDVLAQRTTMGVI----T-GDMFVNGKPLDP--AFQRSTGYVQQQ
DLH-LETSTVREALQFSAMLRQPKSVSKQEKHDYVEEVIKMLNMSDFAEA
VVGV--PGEGLNVEQRKLLTIGVELAAKPKLLLFLDEPTSGLDSQSSWSI
ISFLKRL-SSAGQAILCTIHQPSAILFQEFDRLLFLARGGKTVYFGELGE
NSRRLLDYFENNGARQCGEDENPAEYMLEIVNAGQN--------------
-------NN--G-KDWFEVWKDSEEAQGVQREIDRLHESKKHEDL-----
----NLAAETGGEFAMPLTTQIVECTYRAFQQYWRMPSYVFAKFGLVSIA
GLFIGF-SFWK--ADGTKAGMQNIILSVFMVTTIFSSLVQQIQPLFITQR
SLYESRERPSKAYSWSAFMLANIVVEIPYGIVAGILTFASFYYPVV----
--GAGQSSERQGLVLLFFIQLLLFTSTFAAMTIATLPNAETASGLVSLLT
IM-SILFNGVLQTPSQLPKFW-MFMYRVSPFTYWVGGMTTSMVGGRPIVC
SASEVSVLSP--PSG-QTCGQYLNAFVN-----------ATGGALQN--P
--DATADCRYCSYTN--ADQF--LATVSLY-YTERWRNFGIMFAYIFFNI
FVAVATYYLFRVANLSQIVASFKKS--------KKSKGGSK---------
-----------AGEG-------------------------VEMAAQQGAH
PGNRSGEKDA--

>A5H456
---MNASNSSLIAS---DPDGICEKILDVILEYALHKFG---DTKQKLAL
GRPK--FLETIAGF--VVQGRCIQM-CLPAFP--FKSSNKIDKVLGTLP-
--------DKAEELALGRLNTMCAKVQAIHAPGAAL----TIISDGLVYN
-DLLSISDKDTWAYGEALRSMAIAHEFQHIRF--ARIRDLIKF--PGSEV

LNEITYVASATNF-RRSLLNEFGKDD---IDIDHEIA-TSEDTKMTYLGY
RRFLE-----------SDLKHIFPLGSDRSANSYKRNVKFLAKEMIIRGY
AFAGAV------RAAFPEHLRLSIHQSTGEHKISISLLHTKTGFTTPWHC
SVARLADGTWV---------------------------------------
------------SAQKADFELQDTMRMICENGRPSYFQEEEASEQHFVPT
QAFGLGGAKMDGSHSLGSATELSLYANETLSQSTSEFDFATLKEQVVVAN
THAVGPTTEQLINTFLRHDYPPLDPKSDSFEARSWLKNFE-GLIEANRAS
NSKQIVGVSYRDLSVEAFWQPSDYQKTFWNQPIAIIDT-IAQKLTASR--
----NVK----RSILKKCDGLIRHGEMLLVLGQPGSGCSTLLKSIAGELD
QLRLGNT--TYM------NYQGVPGH---VMHKEFRGEAV--YQAETDVH
FHQLTVKETLEFAARARAPCESIP-----GVNRDTYVTHVRDAYIAMFGL
RHIADTKVGNAFLRGVSGGEVKRVSIAEAAVARSAIQCWDNSTRGLDSAA
ALDFVQTLRTSADIAGTTIAVTLYQAPQSVYNLFDKVSVLYEGRQIFFGP
ASEAKEYFID-LGFEP-KPRQTTADFLTSVTSPAERRI---RKD-FVG--
RIPATPDDFFVVWQKSQQFKHLQDDIDKFNESN-----------------
PI-G-GPSLEEFRNARRSLQEKSQRSRSP--FTLSLPSQIDLCVWRGFQR
LKRDMGILISSIIFNSILSIVIGSVFYGL----PNDNAALYSRGVLLYFS
IMLAAFASAL-EILVLYAQR-PIVEKQARYAFCHPFAEAIA-SMLCDLPN
KITTAIGSSLPLYFMTHLRRTPGHFFVFLVFTFACTLTMSMYFRCIAALS
RTLAQAMAPASVFSLALVIYTGFAIPTRYMR---PWLRWLNYLNPVGYAF
ESLMINEFHDRSIPC--SEYVP----HG--EAYNDIQARERICATS-GST
AGAEAIDGDV-YLAVNFGYHASHLWRNLGIMLALMILGCSIYLLATEYVT
EQKPKGETLLFQRGGIPRNRPQDEESVGNGNIETTSVLMAEPTCKGRVDV
TF--RP
EQESVFHWDDVSF---DIGTKG-SSKRILQGVDGWIRPGTLTALMGVSGA
GKTTLLDVLADRVSVGVV----S-GNMLVDGLPRGP--DFRRQTGYAQQQ
DLH-LASSTVREALNFSALLRQPRTVPNDEKIAYVEEVIAILDMEAYSDA
VVGV--PGEGLNVEQRKRLTIAVELVAKPAVLLFLDEPTSGLDSQTAWSI
CSLLRKL-ADNGQAILCTIHQPSAPLLGLFDRLLYLAMGGRTVYFGALGA
SCSAVIDYFQDKGARPCGGDENPAEWILDVTNTPRN--------------
-------ID--G-TAWADVWDTSEERQAVKAELARMKPSITSPIT-----
----AIDAD--RPYAAAFGTQLGHLLRRGFSHYWRTPSYLWSKVALCVFS
ALFIGV-SFWK--MPNSIQGTQNQLFAVFLLLTIFTNFCQQMMPHAITRR
ELAEARELPSKVYSWQTFILSDIVVEVPWNSLMAVLVFACWYYPIGLQQN
AIDAGQTGERAILMFLFILAFFNFAGTFTSMAVALMSTAESAGNITNLLF
SL-SLIFCGVLATPQALPGFW-IFMYRISPLTYLVSGVLSVGLANTRIHC
LDEELLHFSA--PPS-TNCSTYLAPYIQ-----------LEGGYLQEVPD
--SNPAQCVFCTGSQ--TNIF--LKSVSAQ-YGDRWRNLGIVWAYVAFNL
VATVIFYWLARVP----------KK--------SRSK-------------

>O74637

-----MEARRHDPTASVNTEDTANEKSEVGDEKYTDAEVTRLAQQLTRQS

TRFSVSPQNAENPFIETHEDSTLNPHSGNFKAKNWMKNLL-AIQSRDPER
YPKRQAGLAFKNLSVHGFGSPTDYQKDVANSVLEIGAF-FRTMAG-TG--
----KQK----IQILRDFDGLVKNGEMLIVLGRPGSGCSTFLKTIAGEMN
GIFKDAN--SHM------NYQGISDK---EMRNQFRGEAI--YTAETDVH
FPQLSVGNTLKFAALARAPRNRLP-----GVSRDQYAEHMRDVVMAMLGL
SHTINTRVGNDFIRGVSGGERKRVSIAEATLCGSPLQCWDNSTRGLDSAN
ALEFCKTLNLMSKYSGTTCAVAIYQASQSAYDVFDKVTVLYEGRQIYFGR
TTEAREFFTN-MGFHC-PDRQTTADFLTSLTSPAERVV---KPG-FEK--
MVPRTPDEFAKGWKNSAAYKELQKEIDDYNTQY-----------------
PI-G-GESFQQFVESRKAMQSKGQRAKSP--YTLSVAEQVQICVTRGFQR
LKSDYSLTISALIGNTIMALIVGSVFYQL----PDDVTSFYSRGALLFFA
VLLNSFSSAL-EILTLYAQR-PIVEKQARYAMYHPFAEAIS-SMLCDMPY
KILNAITFNVTLYFMTGLRQNAGAFFTFMLFSFVTTLTMSMIFRTIASYS
RTLSQALVPAAILILGLVIYTGFTIPTRNML---GWSRWMNYIDPIAYGF
ETLIVNEFHGRNFPCNPESFIP----AG--DSYADVGRFNKICSAK-GAV
AGQNFVSGEA-YYTASFQYSNSHRWRNMGIMIGFMVFFMVTYLVGTEYIS
EAKSKGEVLLFRRGYAPKNSGNSDGDVEQTHGVSSAEKKDGAGSGGEQES
AA--IQ
RQTSIFQWQDVCY---DVHIKN-EERRILDHVDGWVKPGTCTALMGVSGA
GKTTLLDVLATRVTMGVV----S-GEMLVDGRPRDQ--SFQRKTGYVQQQ
DLH-LHTTTVREALRFSAILRQPRHVSHQEKLDYVEEVIKLLGMEHYADA
VVGV--PGEGLNVEQRKRLTIGVELAAKPQLLLFLDEPTSGLDSQTSWSI
LDLIDTL-TKHGQAILCTIHQPSAMLFQRFDRLLFLAKGGRTVYFGEIGE
HSSTLSNYFERNGAPKLSPEANPAEWMLEVIGAAPG--------------
-------TH-SD-IDWPAVWRESPERKAVQNHLAELRNNLSLKPV-----
-ATTDNDPAGFNEFAAPFAVQLWQCLIRVFSQYWRTPIYIYSKTALCSLT
ALYVGF-SFFH--AQNSMQGLQNQMFSIFMLMTIFGNLVQQIMPHFVTQR
SLYEVRERPSKTYSWQAFMSANILVELPWNALMSVLIFLCWYYPVGLQRN
AS-ADDLHERGALMWLLILTFMLFTSTFSHMMIAGIELAETGGNLANLLF
SL-CLIFCGVLATPDKMPHFW-IFMYRVSPFTYLVSAMLSTGTSGAKVEC
ESVELLHFEP--TAG-KTCFEYMNTYMNGLVVNGTQVAAPAGGYLVDNNA
----TSNCAFCTIAD--TDTY--LASVLSY-YKDAWRNFGIMWAFIIFNI
FGAVCIYWLARVPKGTRSKKTKTA--------------------------

>Q5ATN6

-------MDPITDSRPKAVDRDSTATSCNSTLDENQIENDREVTNLARVL
SERSKHSHHQLP--FNFGKDPSLDPQNAQFNARAWAKAFY-NVRYSSTDA
PPPRVAGVAFKNLNVAGYGSPVDYQMSVGNALLKLPTL-AWQWLG-GK--
----KQR----TEILQDVDGLLLPGEQLCVLGPPGSGCSTLLKTIAQETH
GFEVDPS--SYV------NYQGITPK---QMSKKFRGEAI--YTAEVDAH
FPQLVVGDTLYFAALARTPRHVPG-----GMSREEYAVHLRDVIMSTFGI
SHTINTKVGNDFVRGVSGGERKRVTIAEAALSYAPLQCWDNSTRGLDSAN

AVEFCKTLRTQGDVFGITSCVAIYQAPQAAYELFDKVTVLYEGRQIYFGP
AKEAKDYFIR-LGFVC-PEAQTTPDFLTSMSSPVERVI---RPG-FEN--
LVPRTADDFAQRWKESPERQALLRDIDRYNTEH-----------------
PF-N-ATDLDLFSSSRDAEKSKNQRPKSP--YTLSYWGQIRICLWRDFQR
LKNDPSVTLAMLILNFFEALIISSVFYNL----PGNTSSFFSRGALLFMM
VLLSAFSSVL-EIITLYEKR-TIVEKHSRYALYHPSAEAIS-SMIMDMPY
KIVNSLLNSLVLYFMGNLRREAGAFFFLYLISFAMMMGMSMFFRFFASLT
KSIEQALAPSSIILLALVLYTGFAIPVSYMR---GWASWIRWLNPVSYGF
EAVMVNEFNGREFPCV--SFVP----SG--PGYENVSPQERVCSTV-GAV
PGSDVVQGAD-FVRSSYGYEYSHRWRNFGIIVALTVFLTMCHLVTSELVS
SQRSKGEVLVFRRGKAQK-MREKRHHTDEEQIAAPAAQNEKVIQEEPGLV
SG--VE
QQSSIFHWEDVTY---DIKIKG-ETRRILDRVDGWIRPGTLTALMGVSGA
GKTTLLDVLASRTTIGVV----G-GNMLVDGRSRDE--SFQRKTGYVQQQ
DLH-LHTSTVREALKFSALLRQPPQYSRAERLAYVETVIDLLNMREYADA
IVGV--PGEGLNVEQRKRLTIGVELAARPKLLLFLDEPTSGLDSQTSWSI
CNLMETL-TKNGQAILCTIHQPSAMLFQRFDRLLLLAKGGKTVYFGEVGQ
GARTLMDYFVRNGGSACPPGANPAEHMLEVIGAAPG--------------
-------AQ-TD-IDWPEVWRSSPEYQQVRSELARLRELANRPSP-----
-VSDPNDKSSYAEFAAPFSEQLLQVGRRVFQQYWRSPSYIYSKALLTVGC
SLFIGF-SFFK--ADNTRQGLQNQMFGVFVFLFVVVQLVMQVIPSFVTQR
TLYESRERQSKTYAWQAFVLSNIIVEFVWNTIMAIFCYLVWFYPVGLYQN
ARYTDTIHSRSTLTLLIIWAVFLFASSFAHMLIAGVDSAEMASALSNIFF
IM-MYAFCGILAGPNALPGFW-IFMYRVNPLTYLVSSLLSATLGDAPMHC
ANNEVLTFFA--PPG-LTCREYMEDYRS-----------VNGGYLLNPDA
--QGSDQCQFCSLEY--TNQY--LSSLSID-FNNRWRDFGLLWVYVIVNT
VGAIVFYKLFRVPRAKKD--------------------------------

>B8M520

---------------------MTSPQHGRNEALNTTSEGYYNQTEKSKEE
DEIRRTEGLNPSDDEPPTVFEPVRPGDREELTRIASFLSSRRPSTLGRR-
--DSAAITRMDTIDEMALEDPRIDPGNPKFDAYLWSRKRL-RILQQEG-M
-EFPRAGVTFKNLNVSGSGEALQLQETVGSYLTAPLRP-GKFFSFAKK--
----TEH----KTILRNFDGVIKGGELLMVLGRPGSGCSTFLKTISGELH
GLNLDKD--STI------HYNGIGMK---KMHSEYKGEVL--YNQEVDKH
FPHLTVGQTLEFAATARAPSKRVL-----GQTRADYVRDVTQVVMAVFGL
SHTYNTIVGNDYVRGVSGGERKRVSIAEMALARAPIAAWDNSTRGLDAAS
ALEFVKALRMASNLAGSCHSVAIYQASQAIYDVFDKVTVLYEGRQIYFGP
CNRAEQYFSK-MGWAK-PSRQTTGDFLTSVTNPQERRA---RDG-MEK--
QVPRTPDEFEAYWKKSPEYAAVLQEIKDHEAQF-----------------
PV--GHVAEKDLAEKKHGQQAKHVRPKSP--YLMSIWMQIRLCTKRAYQR
IWNDKATTLTTVLGRIFMALIVGSIFYGT----PAATAGFQSKGAVLFFA
VLLNALISIT-EINSLYDQR-PIIEKQASYAFVHPFAEAMG-DIMADLPI

KFASAAAFNIVLYFLAGLRYEPSQFFIFFLFTFIATLAMSAIFRTLAAAT
KSLAQAMALAGVMVLAIVIYTGFVIPGPQMH---PWFSWIRWINPVFYTF
EALVANEFHGREFIC--SNFVP----A-----YPNLAGDTFVCSAT-GSV
TGRRTVSGDQ-YIQYQYNYSYSHEWRNFGILIAFWIFFMFTYLTCTELNS
ATSSTAEFLVFRRGRVPAYMTKSDN-DVKNGTIEVP--TGDSESAKEEVV
NM--LP
EQRDIFTWRNVCY---DIPVKG-GQRRLLDNVSGWVKPGTLTALMGVSGA
GKTTLLDVLAQRVSIGVV----T-GDMFVNGKPLDA--SFQRKTGYVQQQ
DLH-LQTSTVREALRFSAALRQSKSTPLSEKYEYVEDVIKMLNMEDFAEA
VVGT--PGEGLNVEQRKLLTIGVELAAKPALLLFLDEPTSGLDSQSSWSI
CAFLRKL-ADHGQAVLSTIHQPSAILFQQFDRLLFLAKGGKTVYFGDIGK
DSRTLLDYFEANGARKCDAAENPAEYMLEIIGAGAS--------------
-------GK-AT-QDWPTVWKESQEAKNIQTELDEIHAHHAATTP-NG--
-TETLTTKSDQSEFAMPFIDQVWHVVIRVFQQYWRDPQYIFAKLVLGLAS
SLFIGF-SFFL--PNNSIQGFQDVLFSTFMLTSIFSTLVQQIMPRFVNQR
SLYEVRERPSKAYSWAAFLIANIVVEIPYQIMLGILVWACYYFPIFGANQ
S------ALQQGLMLLFVVQFFIFASTFADLVIAAMPNAQMAGTIATLAF
SL-TLTFNGIMQPPNALPGFW-IFMYRVSPLTYLIAGMTGNGLDGRVVRC
SDHELSVFNP--PSG-TTCGAYLQRYLE-----------LAPGKLY--NP
--DAMSDCHYCSLQS--ANQV--LASSNIY-PGERWRNWGIGWAYIGFNI
FGAVALYYAFRVKHYNPTSIVRGIHDAGRFVGRMFSRKS-E---------
-----------TPTGRKAQDGRVF--------------------------

>A1CQ14

---------------------------MEHHYGTHNDAIQPGTSSRGISD
VTQESHGPNEKRGREDSAVLENAPPEYRAELARIASNFP-KHRPTDSGT-
--IGAGIERKDTQEDIDLNDPVYDPTSDQFDHYKWVRMVL-KLLDKEG-F
PRPPSTGIVFQHLTVSGSGSALQYQHTVDSILLAPFRP-QEYLSFAKH--
----SPQ----RQILRNFDGLLKSGELMIVLGRPGSGCSTFLKSLCGELH
GLKLSKD--SEI------QYNGISME---RMHKEFKGEVL--YNQEVDKH
FPHLTVGETLEFAAAARTPEHRLR-----GISRHRHAKLVTQVVMTIFGL
SHTYNTKVGDDYIRGVSGGERKRVSIAEMALSGAPVGAWDNSTRGLDSAS
ALEFVKALRVSANLVGTSHAVAIYQASQAIYDVFDKAIVLYEGREIYFGP
CNEAKEYFIN-MGWHC-PPRQTTGDFLTSVTNPQERQA---REG-MEN--
RVPRTRDDFENYWKNSSQYARLQKEIAEHLREY-----------------
PP--GGESAQALYRVKRFKQAKHVRPKSP--YIISIPMQVKLCTIRAYQR
IWNDKPSTLTIVIGRISMALIIGSMYYGT----PIATAGFQSKGAALFFA
VLMNALISVT-EINSLYDQR-PIIEKQASYAFVHPFAEAFG-GIVSDIPV
KFVSAVMFNIIFYFLAGLRYEPSQFFIFFLFTFISTLAMSGIFRTLAAAT
KTLSQAMALAGVIVLAIVIYTGFVIPVTQMSS-IPWFSWIRWINPVFYTF
EALVANEFHGRRFEC--SQFVP----A-----YPSLSGNSFICSVR-GAV
AGERTVSGDS-YIETQYSYTYSHVWRNFGILIGFWIFFTAIYLIASEVNS
ATSSKAEFLVFRRGHVPPQIRDLDKVGRENEMPGPS--TANTSSEAEKDV
SV--IP

EQHDIFTWRDVCF---DIPVKG-GQRRLLDNVSGWVKPGTLTALMGVSGA
GKTTLLDVLAKRVSIGVV----T-GDMLVNGKPPDN--SFQRKTGYVQQQ
DLH-LPTTTVREALRFSAVLRQPKSVPRKEKYRYVEEIIDMLNMKDFAEA
IVGT--PGEGLNVEQRKLLTIGVELAAKPALLIFLDEPTSGLDSQSSWSI
CSFLRKL-ADHGQAVLSTIHQPSAILFQEFDRLLFLQKGGKTVYFGEIGE
QSRILLDYFERNGARVCNSSENPAEYMMEIIGAGAS-------------
-------GK-AS-QDWSVVWNESPEAKKVQEEINRINHERASASS-SD--
-NSA--T--QHGEYAMPFISQLWYVTHRVFQQYWREPGYIWAKIILGAAS
SLFIGF-TFFK--PDSSLQGFQDVLFSAFMLTSVFSTLVQQIMPKFVMQR
SLYEVRERPSKAYSWAAFLIANAAVEIPWQILTGVIAWASYYFPTYGANQ
P------SHRQGLMLLFVVQFYVFTSTFASLVIAALPDAETGGTIATLLF
IM-TLTFNGVMQSPQALPGFW-IFMYRVSPLTYLIAGITATGLHGRAIQC
ARAELSIFNP--PPG-TSCGQYLAPYLQ-----------AAPGQLY--NP
--TATQDCQYCQLRS--ADQY--LATSNIY-YGQRWRNYGLGFAYIGFNI
MGTVMLYYMFRVKHYNPTSLVRGIVSGARFVCRVFKRRSGS---------
-----------TPRGREAENGRLL--------------------------

>B8NJP1

--------------------------------MTEEKRTGSSRSSSIHEDV
TDMRQQYKRRGSKFDDASVFESINPENRAELTRIASNFPLQRRATGSQE-
--SEARLQRKDTLEDIGLDHPSLDPTSGQFDQYKWTRMRL-KLMDKEG-I
PRPPSTGVVFQNLNVSGSGSALQYQSTVGSILLEPFRP-SGWLSFAKK--
----SPE----KHILRNFDGLLKSGEMLIVLGRPGSGCSTFLKTLCGQLH
GLKLRKS--SEI------QYNGVSME---KMHKEFKGEVL--YNQEVDKH
FPHLTVGQTLEFAAAARTPENRLL-----GLKRQQFAKHITKVAMAVFGL
LHTYNTKVGDDYIRGVSGGERKRVSIAEMALSGAPMGAWDNSTRGLDSAS
ALEFVKALRLSSNLVGTSHAVAIYQASQAIYDVFDKAIVLYEGREIYFGP
CDEARDYFTG-MGWHC-PPRQTTGDFLTAVTNPQERQA---RDG-MEN--
KVPRTPDDFEKYWKKSPQYAALQQEIDEYHMEY-----------------
PV--GGEAEQSFGEMKRVKQAKHVRPESP--YIISIPMQVKLCTIRAYQR
LWNDKPSTLTTVLGRIFMALIIGSMYFGT----PTASAGFYSKGAALFFA
VLMNALISIT-EINSLYDQR-PIVEKQASYAFVHPFTEAFG-GIVSDIPV
KFVSAVIFNIIFYFLAGLRYEPSQFFIFFLFTFLSTLAMSGIFRTLAAAT
KTLAQAMAMAGVLVLAIVIYTGFVIPVPQMHD-IPWFSWIRWINPIFYTF
ESMIANEFHGRQFIC--SQFVP----A-----YPSLSGDSFICSVR-GAV
AGERTVSGDA-FIESQYTYTYTHEWRNLGILIGFWIFFSVIYLLATEINS
QTSSKAEFLVFRRGHVPAHMRDLDKTQGDSGSTEVA--QSHKEKETENAA
SV--IP
KQRSIFTWRNVCY---DIPVKG-GQRRLLDHVSGWVKPGTLTALMGVSGA
GKTTLLDVLAKRVSIGVV----T-GDMLVDGKTLDN--SFQRKTGYVQQQ
DLH-LATTTVREALRFSALLRQPKSVSRKEKYDYVEEVIEMLNMQDFAGA
IVGT--PGEGLNVEQRKLLTIGVELAAKPELLIFLDEPTSGLDSQSSWSI
VAFLRKL-ADHGQAVLSTIHQPSALLFQQFDRLLFLAKGGKTVYFGEIGD
QSRTLLDYFEGNGARACGPEENPAEYMLEIIGAGAS-------------

```
-------GK-AS-KDWSAVWNESPESSNVQKEIDRIYQERASASN-GS--
-GDT--HHGKPAEYAMPFMYQLWYVTHRVFQQYWREPAYVWAKILLATLS
SLFIGF-TFFK--PNSSQQGFQDILFSAFMLTSIFSTLVQQIMPKFVVQR
SLYEVRERPSKAYSWAAFLIANVIVEIPYQILAGVISWACYYYPIYGANQ
A------SQRQGLMLLFIVQFYIFTSTFATFIISALPDAETGGTIATLLF
IM-ATTFNGVMQPPNALPGFW-IFMYRVSPLTYLIAGMTATGLHGRAIRC
DTAELSVFNP--PSG-STCGDYLAPYLQ-----------SAPGVLY--NP
--SATQGCEYCQLRN--ADQY--LASSNIY-YSERWRNYGLGWAYIGFNV
MGTVALYYLFRVKHYNPTSLVRGVANGAKLVCRVFKRRSGT---------
-----------TPRGREAENGRLV--------------------------
--------------------------------------------------
--------------------------------------------------
-----------------------------
>Q0D180
--------------------------------------------------
--------------------------------------------------
--------------------------------------------------
--------------------------------------------------
--------------------------------------------------
--------------------------------------------------
--------------------------------------------------
--------------------------------------------------
--------------------------------MAHPESTSDSSRSSSHEP
DEIAPIRTFSRRQSRGAREDSTVMDHMRPEDREQLTRIASGFPRHRPVD-
--SGAAIERRDTLDELNPDDPVLDPSSDKFDLYKWARMRM-KLMDTEG-L
PRPASAGVVFQNLNVSGSGSELQYQDTVASTLLAPFRP-QEYLSFAKR--
----SPE----KHILRNFDGLLQSGELLIVLGRPGSGCSTFLKTLCGELH
GLKLRKS--SEI------QYNGISME---RMHKEFKGEVV--YNQEVDKH
FPHLTVGQTLEFAAAARTPERRLH-----GVNRQQYAKHITQVIMAVFGL
SHTYNTKVGDDYVRGVSGGERKRVSIAEMALSGAPIAAWDNSTRGLDSAS
ALEFVNALRLSANLAGSCHAVAIYQASQAIYDVFDKAIVLYEGREIYFGP
CDEARDYFID-MGWDC-PPRQTTGDFLTSVTNPQERKP---RQG-MEN--
KVPRTPDEFEKYWKNSPLYAELHKEIKEHMEEF-----------------
PP--GGESEQVFGERKRQRQAKHVRPKSP--YVISIPMQVKLCTIRAYQR
IWNDKPSTLTTVIGRIAMSLIIGSIYYGT----PNATAGFQSKGAALFFA
VLMNALISIT-EINSLYDQR-PIVEKQASYAFVHPFTEAFG-GIVSDIPV
KFVSATVFNIIFYFLAGLRYEASQFFIFFLFTFLSTFAMSGIFRTLAAAT
KTLAQAMAMAGVIVLAIVIYTGFVIPVPQMSD-IPWFSWIRWINPVFYTF
EALVANEFHGRRFTC--SQFVP----A-----YPQLSGDSFICNVR-GAV
AGERTVSGDA-FIESQYRYTYAHEWRNLGILIGFYIFFTVVYLLATELNS
ATSSKAEFLVFRRGHVPAYMRDANKRGKES--VATD--NPQHQAETEKDA
SA----------------------------------------------IP
KQHAIFTWRDVCY---DIPVKG-GQRRLLDHVSGWVKPGTLTALMGVSGA
GKTTLLDVLAKRVSIGVV----T-GDMLVDGKGLDN--SFQRKTGYVQQQ
DLH-LATTTVREALRFSALLRQPISVSKKEKYKYVEEVIAMLGMEDFAGA
IVGT--PGEGLNVEQRKLLTIGVELAAKPALLIFLDEPTSGLDSQSSWSI
IAFLRKL-ADHGQAVLSTIHQPSALLFQQFDRLLFLAKGGKTVYFGEIGD
QSRTMLDYFEANGARTCGASENPAEYMLEIIGAGAS--------------
-------GK-AS-KDWAAVWNESQEAKDIQKEIDRIHQERASASD-EH--
-GDD-SPGSEYGEYAMPFPNQLWHVTHRVFQQYWREPAYVWAKLLLATLS
SLFIGF-TFFK--PNSNLQGFQDVLFSAFMLTSIFSTLVQQIMPKFVVQR
SLYEVRERPSKAYSWAAFLIANVAVEIPYQILAGVIAWACYYFPIYGASQ
A------SHRQGLMLLFVVQFYMFTSTFAGLIISALPDAETGGTIATLLF
IM-ALTFNGVMQPPNALPGFW-IFMYRVSPLTYLIAGLTATGLHGRPIEC
```

SSEELSVFNP--PAG-MTCGQYLASYLQ-----------AAPGTLY--NQ
--DATQGCEYCQLRN--ADQY--LASSNIF-YGERWRNYGLGWAYIGFNI
MGTVALYYMFRVKHYNPTSLMVKHIDV-----------------------

>B8NW48

---------------------------------MASHKKSEDPLVVKDRQ
EQECESSDSTIASENASEHRSPMGLIDEDGIETLNRIASQSSRRRSSVY-
--PPNVPTRTSTLATISENDPAVDPQGPSFDLNKWLKMVL-RESERQG-R
-EAHRTGIVFKNFTVSGTGAALQLQDTVSSMLSAPFRI-GEMMK-NRH--
----SPP----KRILNEFNGLLKSGELLLVLGRPGSGCSTFLKSLCGELH
GLSMSKE--SVI------HYDGVPQQ---RMIKEFKGEVV--YNQEVDKH
FPHLTVGQTLEFAALARTPAQRIR-----DMSREEFAKHITQVVMAVFGL
SHTYNTKVGNDFVRGVSGGERKRVSIAEMALAHSPLAAWDNSTRGLDSAT
ALKFVEALRLFADLSGSAHAVAIYQASQSIYDIFNKVVVLYEGRQIYYGP
AKDAKSYFER-QGWEC-PQRQTTGDFLTSVTNPSERKA---RPG-MEN--
QVPRTAEDFEAYWRKSPEYQKLMSEISHYEQEH-----------------
PLEEEGDALATFQQKKREIQAKHTRPQSP--YLLSVPMQIKLNTKRAYQR
VWNDISSTVSTVISQIIMALIIGSVFYGT----PDATAGFTAKGATLFFA
VLLNALIAMN-EINSLYSQR-PIVEKHNSYAFYHPATEAIA-GVVSDIPV
KFVIAVVFNLILYFLAGLHRSAGQFFLYLLVTFIVMFVMSAVFRTMAAIT
QTVSQAMGLAGILILALIVYTGFVLPVPSMH---PWFEWIHYLNPIYYAF
EMLIANEFHGRDFIC--SQFIP----A-----YPSLSGNSFVCSSA-GAK
AGQRAISGDD-YILVNYQYSYGHVWRNFGILIAFLVGFMMIYFIATELNS
STSSTAEVLVFRRGHEPAYLRTDSKKPDAESAVELS-AMKPTTESGEGDM
SI--IP
PQKDIFTWRDVCY---DIEIKG-EPRRLLDHVSGWVKPGTLTALMGVSGA
GKTTLLDVLAHRTSMGVI----T-GDMFVNGRGLDQ--SFQRSTGYVQQQ
DLH-LETATVRESLRFSALLRQPPNVSIQEKYDYVEDVIRMLKMEDFAEA
VVGV--PGQGLNVEQRKLLTIGVELAAKPKLLLFLDEPTSGLDSQSSWAI
CAFLRRL-ADSGQAVLCTIHQPSAILFQQFDQLLFLARGGKTVYFGPIGQ
NSNTLLNYFESNGARKCANDENPAEWMLEIVNNGTN--------------
-------SE--G-ENWFDVWKRSSECQGVQTEIDRIHREQQSKTQ-AS--
-DKD-NESWSKSEFAMPFWFQLYQVTYRVFQQYWRMPEYIASKWVLGILA
GLFIGF-SFFQ--AKSSLQGMQTIVYSLFMLCSIFSSLVQQVMPLFVTQR
SLYEVRERPSKTYSWKAFLIANIIVEIPYQIMMGILTYACYYYAV-VGVQ
D------SERQGLVLLLCIQFFIYASTFAHMAIAAMPDTETASAIVVLLF
AM-SLTFCGVMQTPTALPGFW-IFMYRVSPFTYWVSAMAATQLHDRVVQC
SPSEMSIFDP--PSG-QTCGEYMSSFMS-----------MAGGQLS--NP
--NATSDCNYCSVAV--ADDF--LSSVNIY-WSERWRNFGLMWVYIVFNI
FLATMLYYTFRVKKWNLSGLKERFSKKK----------------------

>Q2TXA7

---------------------------------MASHKKSEDPLVVKDRQ
EQECESSDSTIASENASEHRSPMGLIDEDGIETLNRIASQSSRRRSSVY-
--PPNVPTRTSTLATISENDPAVDPQGPSFDLNKWLKMVL-RESERQG-R
-EAHRTGIVFKNFTVSGTGAALQLQDTVSSMLSAPFRI-GEMMK-NRH--
----SPP----KRILNEFNGLLKSGELLLVLGRPGSGCSTFLKSLCGELH
GLSMSKE--SVI------HYDGVPQQ---RMIKEFKGEVV--YNQEVDKH
FPHLTVGQTLEFAALARTPAQRIR-----DMSREEFAKHITQVVMAVFGL
SHTYNTKVGNDFVRGVSGGERKRVSIAEMALAHSPLAAWDNSTRGLDSAT
ALKFVEALRLFADLSGSAHAVAIYQASQSIYDIFNKVVVLYEGRQIYYGP
AKDAKSYFER-QGWEC-PQRQTTGDFLTSVTNPSERKA---RPG-MEN--
QVPRTAEDFEAYWRKSPEYQKLMSEISHYEQEH-----------------
PLEEEGDALATFQQKKREIQAKHTRPQSP--YLLSVPMQIKLNTKRAYQR
VWNDISSTVSTVISQIIMALIIGSVFYGT----PDATAGFTAKGATLFFA
VLLNALIAMN-EINSLYSQR-PIVEKHNSYAFYHPATEAIA-GVVSDIPV
KFVIAVVFNLILYFLAGLHRSAGQFFLYLLVTFIVMFVMSAVFRTMAAIT
QTVSQAMGLAGILILALIVYTGFVLPVPSMH---PWFEWIHYLNPIYYAF
EMLIANEFHGRDFIC--SQFIP----A-----YPNLSGNSFVCSSA-GAK
AGQRAISGDD-YIQVNYQYSYGHVWRNFGILIAFLVGFMMIYFIATELNS
STSSTAEVLVFRRGHEPAYLRTDSKKPDAESAVELS-AMKPTTESGEGDM
SI--IP
PQKDIFTWRDVCY---DIEIKG-EPRRLLDHVSGWVKPGTLTALMGVSGA
GKTTLLDVLAHRTSMGVI----T-GDMFVNGRGLDQ--SFQRSTGYVQQQ
DLH-LETATVRESLRFSALLRQPPNVSIQEKYDYVEDVIRMLKMEDFAEA
VVGV--PGQGLNVEQRKLLTIGVELAAKPKLLLFLDEPTSGLDSQSSWAI
CAFLRRL-ADSGQAVLCTIHQPSAILFQQFDQLLFLARGGKTVYFGPIGQ
NSNTLLNYFESNGARKCADDENPAEWMLEIVNAGTN--------------
-------SE--G-ENWFDVWKRSSECQGVQTEIDRIHREQQSKTQ-AS--
-DKD-NESWSKSEFAMPFWFQLYQVTYRVFQQYWRMPEYIASKWVLGILS
GLFIGF-SFFQ--AKSSLQGMQTIVYSLFMLCSIFSSLVQQVMPLFVTQR
SLYEVRERPSKTYSWKAFLIANIIVEIPYQIMMGILTYACYYYAV-VGVQ
D------SERQGLVLLLCIQFFIYASTFAHMAIAAMPDTETASAIVVLLF
AM-SLTFCGVMQTPTALPGFW-IFMYRVSPFTYWVSAMAATQLHDRVVQC
SPSEMSIFDP--PSG-QTCGEYMSSFMS-----------MAGGQLS--NP
--NATSDCNYCSVAV--ADDF--LSSVNIY-WSERWRNFGLMWVYIVFNI
FLATMLYYTFRVKKWNLSGLKERFSKKK----------------------

>Q4WE99

--MSVNSQGK
YLSEDPGSQDEETIYESTVRSPIDLIWPDETENLRQIATQQSEKSRRRS-
--EAGAPDVTTR-T-IAEDDPALDPQSAEFNLEKWLRIIV-ADAQGRG-L
-SPPQAGIVFKQLNVSGSGAALQLQDTLGSTLALPFRL-PELLR-QRH--
----SPS----RLILKSFNGLMKSGELLLVLGRPGAGCSTFLKTLCGETH
GLDVDPK--SVL------HYNGVSQT---RMMKEFKGEIV--YNQEVDKH
FPHLTVGQTLEFAAAARTPSHRFH-----DMSRDEYAKYAAQVIMAVFGL
SHTYNTILGNDFVRGVSGGERKRVSIAEMALAATPLAAWDNSTRGLDSAT
ALKFIESLRLLADLAGTAHAVAIYQASQSIYDLFDNVTLLYEGRQIFFGP
TSTAKGFFER-QGWEC-PPRQTTGDFLTSITNPQERRP---RAG-MEK--
IVPHTPEDFEKYWLQSPEYRRLQEQIERFETLH-----------------
PPGDDEKAAAHFRKRKQGVQSKSSRKGSP--YLISVPMQIKLNTRRAYQR
LWNDISSTLSTVIGNVVMALIIGSVFYGT----ANTTAGLSSRGATLFFA
VLLNALTAMS-EINSLYSQR-PIVEKQVSYAFYHPSTEAIA-GVISDIPV
KFVLAVVFNIILYFLANLRREASQFFIYFLITFIIMFVMSAVFRTMAAVT
KTASQAMGLAGVLILALIVYTGFVLPVPSMH---PWFEWIHYINPIYYAF
EILVANEFHGRDFPC--ASFVP----A-----YADLSGDSFSCSTS-GSV
AGQTTVNGDR-FIYYNFKYSYNHVWRNFGILMAFLIGFMAIYFLASELNS
STTSTAEALVFRRNHQPQHMRAENGKSTSDEESGIEMGSVKPAHETTTGE
LT--LP
PQQDIFTWRDVCY---DIEIKG-EPRRLLDHVSGWVKPGTLTALMGVSGA
GKTTLLDVLAHRTSMGVI----T-GDMFVNGKALDT--SFQRKTGYVQQQ
DLH-LETATVRESLRFSALLRQPPTVSIQEKYDYVEEVIRMLRMEEFAEA
IVGV--PGEGLNVEQRKLLTIGVELAAKPKLLLFLDEPTSGLDSQSSWAI
CSFLRRL-ADSGQAILCTIHQPSAILFQEFDQLLFLAKGGKTVYFGPVGD
NSRTLLDYFESNGARKCGELENPAEYMIEVVNAKTN--------------
-------DK--G-QYWYDVWNQSPESRAVQEEIDRIHEERKATHQ-ED--
-DDQ-A----HTEFAMPFWFQLYVVSRRVFQQYWRMPAHIASKWGLAIMA
GLFIGF-SFFD--AKASLAGMQTVLYSLFMVCSVFASLVQQIMPLFVTQR
SLYEVRERPSKAYSWKAFLIANIVVELPYQIVMGILTFACYYFPIVGASQ
S------TERQGLVLLYCIQFYVYASTFAHMVIAAIPDTQTASPIVILLF
SM-MLTFCGVMQSPSALPGFW-IFMYRLSPFTYWVGGMGATQLHDRNVIC
SATELSIFDP--PAN-QTCYEYMAEYMK-----------LAGGQLQ--NP
--NATSECKFCSLTV--ADQY--LAGSEIE-WSQRWRNFGIIWAYVVFNI
FMATVLYYLFRVRKWDIASIKARFGRK-----------------------

>A1D089

--MSVNSQDK
YLLEDPGSQDEETTYESAVRSPMDLIRPNETENLRRIATQQSEKSRRRS-

--EAGAPDVHTT-PAVAEDDPALDPQSVEFNLEKWLRTIV-ADAKGRG-L
-SPPQAGIVFKQLNVSGSGAALQLQDTVGSTLALPFRL-PELLR-QRH--
----SPS----RLILKSFNGLMKSGELLLVLGRPGAGCSTFLKTLCGETH
GLDVDPT--SVL------HYNGVSQA---RMMKEFKGEIV--YNQEVDKH
FPHLTVGQTLEFAAAARTPSHRFR-----DMSRDEHAKYAAQVIMAVFGL
SHTYNTKVGNDFVRGVSGGERKRVSIAEMALAATPLAAWDNSTRGLDSAT
ALKFIESLRLLADLAGTAHAVAIYQASQSIYDLFDNVTVLYEGRQIFFGP
TSTAKGFFER-QGWEC-PPRQTTGDFLTSITNPQERRP---RAG-MEK--
IVPHTPEDFEKYWIQSPEYQRLQGRIEEFETLH-----------------
PPGDDEKAAAHFRKRKQDVQSKNSRPGSP--YLISVPMQIKLNTRRAYQR
LWNDISSTLSTVIGNIVMALIIGSVFYGS----PDTTAGLSSRGATLFFA
VLLNALTAMS-EINSLYSQR-PIVEKQVSYAFYHPSTEAIA-GVISDIPV
KFILAVVFNIILYFLANLRREASQFFIYFLITFIIMFVMSAVFRTMAAVT
KTASQAMGLAGVLILALIVYTGYVLPVPSMH---PWFEWIHYINPIYYAF
EILVANEFHGRDFPC--ASFVP----A-----YADLSGDSFSCSAS-GSV
AGQTTVSGDR-YIFYNFKYSYDHVWRNFGILMAFLIGFMSIYFLASELNS
STTSTAEALVFRRNHQPEHMRAENVKSTSDEESGIEMGSVKPAHETTGE
LT--LP
PQQDIFTWRDVCY---DIEIKG-EPRRLLDHVSGWVKPGTLTALMGVSGA
GKTTLLDVLAHRTSMGVI----T-GDMFVNGKPLDT--SFQRKTGYVQQQ
DLH-LETATVRESLRFSALLRQPPTVSIQEKYDYVEDVIRMLRMEEFAEA
IVGV--PGEGLNVEQRKLLTIGVELAAKPKLLLFLDEPTSGLDSQSSWAI
CSFLRRL-ADSGQAILCTIHQPSAILFQEFDQLLFLAKGGKTVYFGPVGD
NSRTLLDYFESNGGRKCGELENPAEYMIEVVNARTN--------------
-------DK--G-QDWFDVWNQSSESRAVQKEIDRIHEERKSIHQ-ED--
-DDQ-A----HTEFAMPFWFQLYVVSRRVFQQYWRMPEYIASKWGLAIMA
GLFIGF-SFFD--AKTSLAGMQTVLFSLFMVCSIFASLVQQIMPLFVTQR
SLYEVRERPSKAYSWKAFLIANIVVELPYQIVMGILTFACYYFPVVGASQ
S------PERQGLVLLYCIQFYVYASTFAHMVIAAIPDTQTASPVVVLLF
SM-ALTFCGVMQSPSALPGFW-IFMYRVSPFTYWIGGMASTQLHDRNVVC
SATELSILDP--PAN-QTCYEYMAEYMK-----------LAGGQLQ--NP
--DATSDCKFCSLTV--ADQY--LAASEIE-WSQRWRNFGIIWAYVVFNI
FMATVLYYLFRVRKWDLASIKARFGRK-----------------------

>Q6C1A3

---------------------MDNGPIDPNRDVEQEHPVQVDHTEKHESS
SSGAPLTQMSETSEDNTYTATPVPLTDVENMDPDSRDVNDLQRVLSNPES
LHKLQSYTEQISRRMTQDERESLKNNEDTFDLARILDGFV-QKSHEQG-V
-HMRSAGVGWRNLTTDGIDQSTVFVPSVDELLRALATLPVQIAKAFKK--
----KQT----RHIIQNNNGVLKAGEMCLVLGRPGSGCSTFLKTITGQVG
GY-TGVE--GDI------SYDGLSQK---DMLEYFKSDII--YNGELDVH
FPHLTVEETLNFAVGCRTPRQRLD-----GLTRDQYIKNYVQLLATVFGL
RHTYNTKVGNDFVRGVSGGERKRVSIAEALATRASIFAWDNATRGLDAST

ALEYSQAIRATTNILNNASFVAIYQAGEHIYNLFDKVTVLYSGRQIYYGP
ADHAKDYFQR-MGYEC-PPRQTTAEFLTAVTDPLGREP---YPE-MVG--
KVPTTADEFEKYWLASPEFRVVQAEYDDYVGSH-----------------
N---AEETFQNMQDSLSKDKMKRQRKKSP--YLISFAMQMRLLTQRGFER
LKGDMAYQTINVCANIIQALVIGSLFYNI----TESTAGAFSRGGVLFFT
LLFNALASMA-EISHSFSQR-PIIVKQKSYSFYHPAGEALQ-ALLTDIPG
KLVTMICFTLIVYFLTHLNRTAGQFFAHLFILFVTTQCMTAFFQVLASAT
PSVEVANSLAGIGILIIVVYSGYMIPTPTMH---VWFKWLNRANPVAYGF
EALMANEFHNRVMTC--EQIVP----AG--PDYSGMPESNKVCSFS-GST
PGSLVVTGDN-YIKNSYNYSFSHMWRNLGILFAFWMGFVFFNVTFSEYIQ
YHSSSGDVLLFKRGHIPEELQKEGADIDEVIADKA---QADDSEKKMDRL
LS--LD
EERDVFTWQNVDY---VIPIAG-GTRKLLDNVQGYVKPGTITALMGESGA
GKTTLLNVLSQRINFGVI----T-GDMLVNGRPLDR--TFQRRTGYVQQQ
DLH-LAESTVRESLIFSARLRQPSFVPDQEKIDYCDKIIKLLGMEAYAES
LVGE--TGRGLNVEQRKKLSIGVELVAKPSLLLFLDEPTSGLDSQSAWAI
VQFLKNL-AAAGQAILCTIHQPSATLFEEFDRLLLLKKGGQTVYFGDIGK
NSNTLVSYFERQGGRKCAPDENPAEYILECIGAGAT--------------
-------AT-AD-GDWHDKWKNSEEYRQTTDEIAKLQQELAQRPQ-KE--
-LDP----SLQRKYAAPYMTQLRWVLRRTQIQFWRSPGYIMAKFMLLIVG
GLFIGF-SFWD--IKFTLSGMQNAIFAVFMITTLSVPLINQIQSFAFQSR
ELFEVRESSSNTFHWSCLLFSQFISELPYALIGGTIFYCCVYFPTKLGTS
A------RVAGYFYFIYAILFNLYYLSFGLWILYFSPDVPSASIITSLMF
SF-VIAFCGVMQPASLMPGFW-TFMYKLSPFTYIIQAYVGDVMHDRKITC
LPREFSRFNP--PSG-QTCQEYAGKFLS-----------QATGYLE--DP
--NATTQCGYCPYSV--ADEF--IATVGIK-YHYRWRNVGFICAYIIFNI
CAMVLCYYLARVRVWRTGAAIADW-----------KAKRAN---------
-----------KKKAASSEKA-----------------------------

>A8PTZ3

--------------------MPRSEGLGLDPPHDIYPPVSGTNEPSPEGD
NPLHSVVHMTDSPVAQPPSMNMVMSRGDMLNTPQGAYYGHDGMVTS----

------------PSSPIITAGNGTPKELYGGYQRRSRVVVPMTTLDPEGV
TELSRRLSTGPRRRSAWSNSFYDQSQSFGMDPSFSSKSHLAMGAASPMCS
SPYPDARTSPSGIDATTTEHGTFDPFDENNKFDLSYLLHE-IYAEMDQRG
NERRSMGIAFRDLRVTGYGTGAQLNETFGSLLLAPLRIVSGVRNMMHR--
-----PI----KTILQDVEGCVKPGEMLLVLGRPGSGCTSLLKALASYRD
GFRSVD---GTV------LYEGLDHR---SIDGPLRGDVV--YSPEDDVH
FPTLTVGQTLRFASATRAPNSKYRITLGETGDRQEYVDGTREVLATVLGL
RHTYNTKVGNDLIRGVSGGERKRVSIAEAMAARAKVALYDNSSRGLDSST
ALEFVQALRIQTNIADCTTIACIYQAGENITQLFDKVALLNQGHLVYFGP
VALAVDYFKS-IGFEP-LDRQTTADFLVACTDLAGQNV---NPD-FRG--
PIPRSPEEQALAFRQSWVGTANHTEVENYIASM-----------------
MARQTKQNADHYVKLARDERAKYSFHNSR--YLLSWPMQVRLAIQRRAQV
AMGDLGTHITVIFAALFQALIIGSVFYQM----PQNTSGFFSRGGVLFFS
LLYNSFTGMS-EISLCYEQR-PIVIRQKRFAMLHPSADALG-NTLLDFPI

```
RAISIFVFDIIVYWLTGLSADAGKFFTYLGMTALVTYCMTSFFRMVAACT
KSEPLATTFGGLAVLDVALYTGYMIPRGSMK---PWWIWLSYCNPVAFGF
EVLLANEYRGKFFEC--VQMIP----PG-------KSVENQVCPVM-SAK
PGQPNVSGED-YLSEMYGFSWHNRIRNFVIILAFWIVFILCFLYASDHQV
DPAAIGGELQFERSKAKNKNLSAPTNDQEKTLEEGKPLEPQDLSEAPAVG
RTGGT-------------------------------------------IK
VSDAIFSWDNITY---DVLIKG-KPRRLLNHVSGYVAPGKMTALMGESGA
GKTTLLNVLAQRTDVGVV----G-GDFFVNGKPLPR--SFQADTGYCQQQ
DVH-LAQHTVREALQFSAMLRQPRETPKEERLEYVETVIRLLEMEQFADA
IVGE--VGEGLNVEQRKRLTIGVELAAKPSLLLFLDEPTSGLDAQAAWSI
VRFLKKL-ASEGQAILCTIHQPSGELFNQFDRLLLLQKGGKTVYFGDLGP
NSMTLVNYFEQRTSMKCGENDNPAEYILDVIGAGAT--------------
-------AT-TD-KDWHELFLQSELFTALRRDLDEIYRTRRQIA------
---DSSSSKHAREYAQPFPVQLYEVTKRAFISYWRNPLYLYTKMMLNVVS
GLVVGS-SFWKEGKRNSYIALQNRLFACFLALVASTSLSQHLQPEFIRFR
GLFEVREKPSKMYTWPVMVLSALLVEIPWNIVGGTIYWIPWYYLIQFPFE
S-------KRSGYSWGLYMLFQLYYCTFAQAMAAISPNAMIASILFSTFF
SF-VVVFCGVVQPPPQLPYFWRSWMFQLSPFTWIMEGILGNAIGGAQVHC
EADEMQVVRL--PPG-ATCQSHMEPFSRP-----QSSDPRGNGYYVDNN-
----DGTCSFCLYRY--GDDY--LETVMMD-ASNKYRDLGIIVAYIAFNT
ALLFTLFWLFRIFKFGKNRQGKKKIMPAPGPEANMPIPSSTMQVEAPAMA
VNVPHVVGAGLTNFMSESSQHPNQHEDLSSRHDAATDISHGTYRQADMAT
SKTQLMGDESPQMDHGDFLSGEPAMTSHPLESPQRRPHRNLDSLYLDPPM
NEAPYVEEDHNDGVETQDDTDQHVPRRHKPSGRRSQYRSAKSRRRRRGSR
DHGETLRTPSGYSYYYDATSEPYDDPEQH
>B2VW24
--------------------------------------------------
--------------------------------------------------
--------------------------------------------------
--------------------------------------------------
--------------------------------------------------
-----------------MASNDATPGLSHLPGGWIETPADPSRQQSYIGS
QQQTHNDPAASYPHNGTSAHHTVSQNYYSAGDAGLSSASSNSTAVASTDA
--------------------------------------------------
------------QTSRNITNGHQLQDRVTQGGVTTERTRSNGYIYETSGG
PVEQQVELEHDTSDSELEGEPTATKKSGTATRPSFNSKNSRLMTEEDLFR
ALSRRRTSQSNGLSKTNTGATGHSPDEEDEINNLMSKMFG-RTRQEASEE
EKTRHQGVIFKHLTVKGMGLGAALQPSVGALFLDPVRFTKNLLTKGPRQA
AG-KPPV----RTILDDFSGCIRPGEMVLVLGRPGSGCSTFLKMIGNQRY
GFEEIT---GKV------SYGGTDAD---EMAKKYRSEVL--YNPEDDLH
YATLKVKDTLKFALKTRTPGKESRK---EGESRNDYVNEFLRVVTKLFWI
EHTLGTKVGNELIRGVSGGEKKRVSIAEAMITKASVQSWDNSTRGLDAST
ALEYVQSLRSLTNMAQISCAIALYQAGESLYDLFDKVLLIHEGRCCYFGP
TEKAADYFKS-LGFVK-PDRWTTSDFLTSVTDEHERQV---KEG-WED--
RIPRTGAAFGEAFANSEQANNNFADIEEFEKET-----------------
-------KRQAEQR---HEARTKATKKKN--FTISFPEQVMACTKRQFLV
MVGDPQSLVGKWGGIFFQALIVGSLFYNL----PNNAQGVFPRGGVIFFM
LLFNALLALA-ELTAAFESR-PILLKHASFSFYRPAAYAIA-QTVIDVPL
VLIQVIIFDVVVYFMANLSRTASQFFISVLFLWIITMTMYAFFRAIGSLV
GSLDVATRITGVAVQALVVYTGYLIPPRKMH---PWFSWLRWVNPIQYGF
EGLLTNEFYNLEIDCVPPFIAP----QV-----PNAQEQYQSCAIQ-GNR
PGSLTVAGSD-YIAAAYGYSRTHLWRNFGLICAFFLFFVALTAFGMEIQK
PNKGGGAVTIYKRGQVPKTIEK--------EMETKTLPKDEESGNKEAVT
EKHSSS-------------------------------DNDESDKTVEGVA
```

KNETIFTFQDITY---TIPYEK-GERTLLSGVQGFVKPGKLTALMGASGA
GKTTLLNTLAQRINFGVV----R-GDFLVDGKPLPH--SFQRSTGFAEQM
DVH-ESTATVREALQFSARLRQPKEVPIEEKYEYVEKIIDLLEMRDIAGA
AIGT--TGNGLNQEQRKRLTIGVELASKPELLMFLDEPTSGLDSGAAFNI
VRFLHKL-ADAGQAILCTIHQPSAVLFEHFDQLLLLKSGGRTVYFGDLGH
DSQKLIGYLQDNGAEKCPPNTNPAEYMLEVIGAGNP--------------
-------DY-KG-KDWADVWEKSSENGKLTQEIQEIITNRRNAA------
---KNEEARDDREYAMPYPQQWLTVVKRSFVAIWRDPPYVQGMVMLHIIT
GLFNGF-TFWN--LGQSQIDMQSRLFSVFMTLTIAPPLIQQLQPRFISVR
GIYESREGSAKIYAWTAMVWGTILSELPYRIVSGTIYWCCWYFPPGFPRD
-------TYTAASVWLFVMLFEIFYLGFGQAIASFAPNELLASLLVPLFF
TF-IVSFCGVVVPYASLPSFWQSWMYWLTPFKYLLEGFLALLVEGQVIRC
DSRELAIFPP--PPG-QSCQTYAGQFAR-----------QSGGYVETQP-
----DGNCGFCQYAT--GEAF--AASFNVF-PRYIWRDFGIMWAYIFFNF
AVVFVCTWLYLGGLRQMKTFFSPAA-------------------------
---RKQKK
EMKSKQGGDAA---------------------------------------

>Q2KEJ1

------------MDDRPTPKDTDIPGGFPESPPGPTPLTERNFPLVTSQL

------------RASRSHVDAPTEQIRRPGPAIGGDIGSTSSSLTEKEGL
NNETASQDDGSEKRFAPLRTDSQAVRATGSDARPGFERKATSYTEDDIFR
ALSRRRTGGTHASAPAES-----DEDEGQEIERLVSRMFG-HERQRQSAE
EKTRRSGVIFRDLTVKGVGLGATLQPTVGDIFLALPRKVGHLFTKGPRAA
FA-KPPV----RELISHFDGCVRPGELLLVLGRPGSGCSTFLKTFCNQRA
GFESVL---GDV------SYGGVDAK---TMARDYRGDII--YNPEEDLN
YATLSVKRTLHFALETRAPGKESRL---EGETRQDYIREFMRVITKLFWI
EHTLDTKVGNEYVRGVSGGERKRVSIAEAMIARASVQGWDNSSKGLDAST
AVEYLRSIRAMTNMANTSTAVSLYQAGESLYELADKVLLIDAGKCLYFGP
SEQAKQYFID-LGFHC-PERWTTADFLISVTDPHERHV---RQG-WED--
RFPRTPEQFAEAYRRSNIYRANLEDMSRFEAEQ-----------------
-------QQQVEARAAIEAGKPKRERTKN--YEIPFHKQVIACTKRQFLV
MIGDKASLLGKWGGLVFQGLIIGSLFFNL----PETASGAFPRGGVLFLL
LLFNALLALA-EQTAAFESK-PILLKHKSFSFYRPSAYAIA-QTVVDVPL
VFIQVVLFTVIIYFMSHLARTASQYFIANLILWLVTMTTYSFFRAISAWC
GTLDVATRFTGLAVQILVVYTGYLIPPTSMP---VWFGWLRWINWLQYGF
ECLMSNEFYRQELTCNGPFLVP----QG-----PQAEPQYQGCTLA-GST
PGDSTVSGAN-YIAESFSYTRAHLWRNFGFLWAFFIFFVLLTALGMERMK
PNKGGGAITVFKRGQVPKQLESTIETGGKGKGGNEKDEEVGTTGSDSQAP
VSPREG-------------------------------STEEDDKRSNQVA
ENETIFTFRDVNY---EISSKG-GKRKLLSDVQGYVRPGKLTALMGASGA
GKTTLLNTLAQRIQTGTV----T-GEFLVDGRPLPK--SFQRATGFAEQM
DIH-EPTATVREALQFSALLRQPREVPKQEKLDYCETIIDLLEMRSIAGA
TIGN--VGEGLNTEQRKRLTIGVELASKPELLMFLDEPTSGLDSGAAFNI
VRFLRKL-ADAGQAVLCTIHQPSAILFENFDELLLLKAGGRVVYHGPLGH
DSQDLLGYLEGNGAHKCPPNANPAEYMLDAIGAGDP--------------

-------DY-KG-QDWGDVWQNSKEREARTREIDDMISQRQQAE------
---QTQSLRDEREYAMPLSAQMSAVVRRSFVSYWRNPGYLVGKFMLHILT
GLFNCF-TFFR--IGFASIDYQNRLFSVFMTLTICPPLIQQLQPVFIDSR
QIFQWRENKAKIYSWSAWVTGAVLAEIPVAVLAGAVYFNCWWWGIFGWRD
IMP----ASSSAFAFLMVVLFELYYVSFGQAVAAFSPNKLLASLLVPLFF
TF-IISFCGVVVPPAQIPTFWREWMYWLSPFHYLLEALLGVAVHAQPLEC
SADELARFEP--PPG-SSCEEYAGPYVA-----------QAGGRLRTGQ-
----GGMCELCQYAD--GDQF--AASFSVY-YYNRWRDVGIFCGFIVFNY
AVVYLATWLKF------------KG-------------------------
---RNPLK
MFGKRKSG--

>B8M372

-MAASSNADLRGNHATPSESTNYNDSSSEETEVVPVRSGSSHQRKRDGRD
QKDGDEDSTLARRYTTNSSGKQQTNEDWAEIQRLMSRMFG-RERQENSEE
EKTRHSGVIWKNLNVKGAGLGAALQPTVGDLFLGLPRLVRGLLARGRKGA
GK-NVPV----KTILNDFTGCVRPGELLLVLGRPGSGCSTFLKVIGNQRA
GYESID---GEV------TYGGTDPQ---AMAKNYRSEVS--YNPEDDLH
YATLTVKQTLSFALQTRTPGKESRN---QGESRKDYQKTFLSAITKLFWI
EHTMDTKVGNELIHGISGGEKKRVSIAEAMITKASTQCWDNSTKGLDAST
ALEYVQSLRSLTNMAQVSTLVALYQAAESLYHLFDKVVLIEEGRCAYYGP
IDKAKAYFEN-LGFEC-PPRWTTPDFLTSISDPHARRV---KSG-WED--
RIPRTAEEFESIYLNSDLHKAALEDIRDFEQDL-----------------
-------EKQKEER----EAARNVTKQRN--FTLSFHQQVLALTRRQFLV
MIGDPQSLYGKWGMILFQALIVGSLFYNL----QPTSAGVFPRGGVMFYI
LLFNALLALA-ELTATFSSR-PILLKHKAFSFYRPSAYALA-QVVVDMPL
VAVQVTIFDLIVYFMSDLSRTASQFFINLLILFVLTMTIYSLFRAVGALC
SSLDVATRITGVALQALIVYTGYLIPPWKMH---PWLKWLIWINPVQYAF
EALMANEFYNLSIQCIPPLLIP----EG-----PGASPQHQSCFLQ-GSQ
PDQTTVRGSD-YIKTAYTYSRSHLWRNFGIIIAWLIFFVVLTMIGMELQK
PNKGGSSVTVFKRGQAPKDVDDA-------LKNKISPGDEENGDA--AQT
NVNNTE-------------------------------QEADGEKNVEGIA
KNTAIFTWQHVNY---DIPVKG-SQKRLLDDVQGYVRPGRLTAMMGASGA
GKTTLLNVLAQRVNTGVV----T-GDFLINGRPLPR--SFQRATGFAEQM
DVH-EPTATVRESLRFSARLRQPREVPLKEKYDYCEKIIDLLEMRPMAGA
TVGS--GGSGLNQEQRKRLTIAVELASKPELLLFLDEPTSGLDSLAAFNI
VRFLRRL-ADAGQAVLCTIHQPSAVLFEQFDDLLLLKSGGRVVYHGELGS
DSRTLIDYFERNGGKKCPRDANPAEYMLEVIGAGNP--------------
-------DY-KG-KDWGDVWANSEEHEARTREIDEIVSSRREGQ------
---TSQETKDNREYAMPIWTQISTTTKRSFVAYWRSPEYLLGKFMLHIFT
GLFNTF-TFWK--LGHSYIDMQSRLFSVFMTLTISPPLIQQLQPRFLHFR
NLYESREAKAKIYSWPAFVVSAILPELPYSLVAGSIYYNCWYWGIRFSHD
-------SFTSGFTYIMILLFELYYVGFGQFIAALSPNELFASLIVPAFF
TF-VVSFCGVVVPYSGLPSFWKAWMYWLTPFHYLLEAFLGVLVHNQPVKC

VAREEARFTP--PPG-STCQNYTSAFTS-----------QAGGYVDDLG-
----NGMCAFCAYSN--GDQF--AASFNVF-YSNKWRDFGIFWAFILFNF
FVIFALSWLYLHGIQGIKKVLSIRK-------------------------
---SKSSS
KTKQNSGRTSEKNRSGSTSSGE----------------------------

>A1DMK5

-------------------------------------MDAERDVEQRRE
ANAAPEGQSPQDIAGSAASPAVSGSDTEDSSSIDDEPSTAFEDVSSKSTR
DGQYGDDQINRVLSRRQTSRSEEGPEDMAQIAKLMSRMFG-KERKSVSDE
EKTRHAGVIWKGLTVKGVGLGAALQPTNSDIFLAVPRFIKGFLTRGRKGI
GAGHHPL----RTILDDFTGCVKPGEMLLVLGRPGSGCSTFLKVIGNQRA
GYKSIK---GDV------RYGGADAE---LMADKYRSEVS--YNPEDDLH
YATLTVRDTLLFALKTRTPDKDSRI---PGESRKDYQNTFLSAIAKLFWI
EHALGTKVGNELIRGISGGEKKRVSIAEAMITKASTQCWDNSTKGLDAST
ALEYVQSLRTLTDMANVSTLVALYQASENLYNLFDKVMLIEEGKCAYYGS
AKEAKAYFER-LGFEC-PPRWTTPDFLTSVSDPHARRV---KSG-WED--
RVPRSGEDFQRLYRESDTYRAALQEIEEFEKEL-----------------
-------ETQEHER----EQARQEMPKKN--YTIPFYGQVIVLTRRQFLI
MYGDKQTLVGKWCILVFQALIIGSLFYNL----PPTSGGVFTRGGVMFFI
LLFNALLAMA-ELTASFESR-PIMLKHKSFSFYRPSAYALA-QVVVDVPL
VFVQVTLFELIVYFMANLSRTPSQFFIQFLFIFILTMTMYSFFRALGAVS
ASLDVATRLTGVAIQALVVYTGYLIPPWKMH---PWFKWLIWINPVQYAF
EAIMANEFYNLDIQCVRPNIVP----DG-----PNAQPGHQSCAVQ-GST
PNQLVVQGSS-YIKTAFTYSRSHLWRNFGIIIAWFIFFVALTMLGTELQQ
PNKGGSSVTTFKRNEAPKDVEEA-------VKNKELPEDVESGQKENAVN
ADSEKT--------------------------------QSGEPGGEVKDIA
QSTSIFTWQDVNY---TIPYEG-GQRKLLQDVHGYVKPGRLTALMGASGA
GKTTLLNTLAQRINFGVI----T-GTFLVDGKPLPK--SFQRATGFAEQM
DIH-EPTATVRESLRFSALLRQPKEVPIQEKYDYCEKIIDLLEMRPIAGA
TVGS--GGVGLNPEQRKRLTIAVELASKPELLLFLDEPTSGLDSLAAFNI
VRFLRRL-ADAGQAILCTIHQPSAVLFEEFDDLLLLQSGGRVVYNGELGQ
DSKTLIEYFESNGAKKCPPHANPAEYMLEVIGAGNP--------------
-------DY-KG-KDWGDVWAQSPQCKQLAEEIDKIIGSRRNRE------
---IRQNKDDDRAYAMPIWTQIVAVTKRAFIAYWRSPQYTLGKFLLHIFT
GLFNTF-TFWH--LGNSYIDMQSRLFSIFMTLTISPPLIQQLQPRFLHFR
NLYESREANSKIYSWTAMVTSAILPELPYSVVAGSIYFNCWYWGIWYPRD
-------SFSSGYTWMLLMVFELYYVSFGQFIAAFSPNELFASLLVPCFF
TF-VVAFCGVVVPYVALPHFWQSWMYWLTPFHYLLEGFLGVLTHNIPVRC
VSREVTQFSP--PPG-QTCQSYAGAFAK-----------QAGGYVEDAA-
----GGLCSYCPYSI--GDAF--AASFNVF-YSHKWRAYGIFWAFTMFNF
AAVYFFSWLYLHGVGDLKRWISARK-------------------------
---TKKSV
K---

>A2QVP0

-------------------------------------MDSGRSPPHEQSS
VLEEEKKTSLDSSSGSSSSASPSSGLSRKQSAIPVEHQVSNENLIRQESS
LQRQLTQQDIARALSQRRSTGAAGADDTDQIARLVSRMFG-QERKANSEE
EKTRHLGVVWKDLTVKGVGLGAALQPTNTDILLGLPRLIKGLLTGGRK--
---SAPL----RTILDDFNGCVRPGEMLLVLGRPGSGCSTFLKVIGNQRS
GYKSVE---GDV------RYGGADAE---TMAKNYRSEVL--YNPEDDLH
YPTLTVRDTLMFALKSRTPDKSSRL---PGESRKHYQETFLSTIAKLFWI
EHALGTKVGNELIRGVSGGEKKRVSIGEALITKASTQCWDNSTKGLDAST
ALEYVESLRSSTDMAHASTLVALYQASENLYNLFDKVMLIEEGKCAYYGR
TENAKAYFER-LGFVC-PPRWTTPDFLTSVSDPYARRI---KEG-WED--
RVPRSGEDFQRAYQKSEICKEAKADIEDFEKEI-----------------
-------ESEQRAC----EQARERKRKQN--YTVSFYKQVIILTQRQFLV
MYGDKQTLIGKWVMLTFQALIIGSLFYDL----PPTSAGVFTRGGVMFYV
LLFNSLLAMA-ELTALYGSR-PVILKHKSFSFYRPAAYALA-QVVVDVPI
VFVQVTIFELIVYFMSNLSRTASQFFINFLFVFILTMTMYSFFRTIGALS
ASLDVATRVTGVSVQALIVYTGYLIPPWKMH---PWLKWLIWINPLQYAF
EAIMSNEFYDLDLQCVSPSIFP----DG-----PSAQPGNQVCAIQ-GST
PNQLVVQGSN-YIEAAFTYSRSHLWRNFGIVIAWFVLFVCLTMVGMELQK
PNKGGSTVTIFKKGEAPEAVQEA-------VKNKELPGDVETGSDGAGAT
SGFQEK-------------------------------GTDDSSDEVHGIA
QSTSIFTWQGVNY---TIPYKD-GQRKLLQDVQGYVKPGRLTALMGASGA
GKTTLLNTLAQRINFGVV----T-GTFLVDGKPLPK--SFQRATGFAEQM
DIH-EPTATVRESLQFSALLRQPKEVPIKEKYEYCEKIIDLLEMRPIAGA
IVGE--GGAGLNAEQRKRLTIAVELASKPQLLLFLDEPTSGLDSLAAYNI
VRFLRRL-ADAGQAILCTIHQPSAVLFEQFDELLLLQSGGRVVYNNELGT
DSKKLIEYFEQNGARKCSPHENPAEYMLDVIGAGNP--------------
-------DY-KG-QDWGDVWARSTQHKQVSQEIENIIQERRNRE------
---VEGEKDDNREYAMPIWVQILTVSKRSFVAYWRTPQYALGKFLLHIFT
GLFNTF-TFWH--LGNSYIDMQSRMFSIFMTLTIAPPLIQQLQPRFLHFR
NLYESREAGSKIYSWTAFVTSAILPELPYSVVAGSIYFNCWYWGVWFPRN
-------SFTSGFIWMFLMLFELFYVGLGQFIAAFSPNPLFASLLVPTFF
TF-VLSFCGVVVPYSSLNVFWRSWMYWLTPFHYLLEGFLAVVVHGVPVRC
VPREASEFSP--PSG-MTCQEYAGSYAS-----------QIGGYVQDAG-
----NGLCAFWRPQT--LMPYSQARNYNVY-YSHKWRNYGIFWAFVIFNF
MAVFFFSWLYLHGVRNMKKSISARK-------------------------
---SKKAV
KQ--

>Q2UM62

--MDVH
HGHLSRDTASDLSSVSASSSNISSNSCPDQNAELNETEKSSRPGSLNRRL
TEDEIVRVLSRRRTGGSGENTEGKSEDMTQIMKLVSRMFG-HERKSNSDE
EKTRHLGVVWKHLTVKGVGLGAAIQPTNSEILLALPRKIKSLLTRGRN--
---KPPL----RTIIDDFTGCVRPGEMLLVLGRPGSGCSTFLKVVGNQRS
GYKSVE---GDV------RYGGADAQ---TMADKYRSEVL--YNPEDDLH
YPTLTVRDTLLFALKTRTPNKESRL---PGESRKEYQETFLSAIAKLFWI
EHALDTKVGNELIRGISGGEKKRVSIAEALVTRASTQSWDNSTKGLDAST
ALEYVQSLRSLTDMANVSTLVALYQASENLYKLFDKVIFIEEGKCVYYGR
AESARHYFES-LGFEC-APRWTTPDFLLSVTDPQARRV---RQG-WED--
RIPRTAEEFRKIYRKSDIYKAALADNESFEEEL-----------------
-------ESHQEER----EAARKQSEKKN--YTVSFYQQVAILTHRQFLI
MYGDKTTLIGKWVILTGQALITGSLFYDL----PQTSAGVFTRGGVMFYV
LLFNALLAMA-ELTSFFDTR-PVILKHKSFSFYRPSAFALA-QVIVDIPI
IFVQVTLFELIVYFMANLSRTASQFFINFLFIFTLTMTMYSFFRTIGALC
GSLDIATRITGVAIQALVVYTGYLIPPWKMH---PWLKWLIWINPVQYAF
EGIMSNEFYNLDIQCEPPSIVP----DG-----PNASPGHQTCAIQ-GSS
ANQLIVRGSN-YIKSAFTYSRSHLWRNFGIIIAWLALFIALTMLGMELQK
PNKGGSAATIFKRGEEPETVRRA-------LENKKLPEDVESGNKEKGVD
GNMNES-------------------------------ASEDSGEKVTGIA
QSTSIFTWRNVNY---TIPYKG-REKKLLQDVQGYVKPGRLTALVGASGA
GKTTLLNTLAQRINFGVV----T-GEFLVDGRPLPR--SFQRATGFAEQM
DIH-EPTATVRESLRFSALLRQPKEVPIHEKYDYCEKILDLLEMRSIAGA
TVGS--GGIGLSEEQRKRLTIAVELASKPQLLLFLDEPTSGLDSLAAFNI
VRFLRRL-ADAGQAILCTIHQPSAVLFEHFDDLVLLQSGGKVVYNGELGQ
DSSKLISYFERNGGKKCPPHANPAEYMLEVIGAGNP--------------
-------DY-EG-QDWSEVWAKSSENKQLTEEIDSIIQSRRNKN------
---EGDNDDDRREYAMPIGVQVVAVTKRAFVAYWRSPEYNLGKFLLHIFT
GLFNTF-TFWH--LGNSYIDMQSRLFSIFMTLTIAPPLIQQLQPRFLHFR
NLYESREANSKIYSWVAFVTSAILPELPYSIVAGSIYFNCWYWGVWFPRD
-------SFSSGYVWMLLMLFEMFYVGFGQFIAALAPNELFASLLVPCFF
IF-VVSFCGVVVPYKALIHFWRSWMYWLTPFHYLLEGLLGVVTHNVPLRC
VSREESQFSP--PPG-ETCQSYAGPFAQ-----------QAGGYVHDTG-
----NGLCSYCQYSD--GDTFA-AESFNVY-YSHKWRAYGIFWAFVMFNF
AAVYAFSWLYLHGIRDIKKWFSTRK-------------------------
---TKRGA
QA--

>B6GZZ9

---MANDHSEES
ASTPGANPEHIPRNSPSSESSSSPVSSSPTTDHAPTRPTPSQSQADSTGP

KSDEYGAARTSSRRSTHASGHDTKGEEWAQIERLISRMFG-PERKANSEE
EKTRHVGVVWKNLTVKGVGLGAALQPTNGDIFLGLPRLIKGLFTRGRKGA
GRGKPPI----RTILEDFTGCVRPGEMLLVLGRPGSGCSTFLKVLGNQRA
GYESIE---GNV------QYGGTESE---KMAKQYRSEVL--YNPEDDLH
YATLTVRDTLLFALKSRTPGKASRI---PGESRKEYQQTFLSAIAKLFWI
EHALGTRVGNELIRGISGGEKKRTSIAEAMVTKASTQCWDNSTKGLDAST
ALEYVQSLRSLTNTANVSTLVALYQASENLFDLFDKVILIDDGKCSFFGP
SQDAKAYFEG-LGFEC-PPRWTTPDFLTSVSDPHARRV---KDG-WDN--
RIPRNAAEFQAAYRKSDTYKRNLADIESFEGEI-----------------
-------EGQRQER----EAARRKAKRKN--FTISFYKQVMILTHRQFLV
MFGDRESLIGKWSVITFQALITGSLFYNL----PDTSNGVFTRGGVMFFI
LLFNALLAMA-ELTAAFESR-PILMKHKSFSFYRPAAYALA-QVVVDVPL
VFIQVVLFDIVVYFMANLARTPSQFFINLLVIFILTMTMYSFFRALGALC
SSLDVATRLTGVAIQALVVYTGYLIPPWKMH---PWLKWLIWINPVQYAF
EALMANEFYNLQIKCEPPYVVP----DG-----PNVVPGHQSCAIQ-GSD
PDQLIVNGSR-YIQTGFTYSRAHLWRNFGIIIGWLILFVSLTMLGMELQR
PNKGGSAVTVFKRSEAPKAVQDV-------IKGSSPQRDEESAEKD-GIA
SNKNDS-------------------------------DTSVSSGKVQDIA
KNTAIFTWQDVNY---TIPYKG-GQRQLLQNVEGYVKPGRLTALMGASGS
GKTTLLNALAQRINFGVV----T-GSFLVDGRPLPR--SFQRATGFAEQM
DIH-EPTATVRESLRFSALLRQPKEVPLQEKYDYCETIIDLLEMRPIAGA
TVGS--AGSGLNQEQRKRLTIAVELASKPELLLFLDEPTSGLDSLAAFNI
VRFLRQL-ADAGQAVLCTIHQPSAVLFENFDELLLLKSGGRVVYNGPLGN
DSKTLIDYFEQNGGRKCSPHENPAEYMLEVIGAGNP--------------
-------DY-KG-QDWGNVWANSPESKQLSEELEGIIASRQNAG------
---SDGKTNDHREYAMPLYVQVAAVTKRAFVAYWRTPEYILGKMMLHIFT
GLFNTF-TFWH--LGNSFIDMQSRLFSVFMTLTIAPPLIQQLQPRYLHFR
GLYKSREANSKIYSWAAFVTSTIVPELPYSIVAGSIYFNCWYWGTWFPRD
-------SFSSGYVWMSLMLFEVYYIGLGQFIAALAPNELFASLLVPTFF
TF-IASFCGVVVPYPALPHFWQSWMYWLTPFHYLLEGLVGVITHNVPVRC
IDREESRFST--PAG-MNCQDYAGSFAE-----------KAGGYVRDAG-
----NGMCSFCQYSTELCHTFQ-AKSLNVF-YSHKWRDYGILWAYIIFNF
ALVFAFSWLYLHGVSNLKRWFSARK-------------------------
---ARKSK
DR--

>A6ZPG0

---------------MLQAPSSSNSGLNQGNAAPDGPPNETQPYEGLDAA
AQEEIKELARTLTSQSSLLSQ---EKRITGTGDPNTLTAASSSSSSRSIF
ASDIKGVNPILLDVNDPDYDETLDPRSENFSSVRWVRNMA-QICENDSDF
YKPFSLGCAWKDLSASGDSADITYQGTFGNMPIKYLKMSWRCISRRLFHR
AHGKSEDNDSGFQILKPMDGCINPGELLVVLGRPGAGCTTLLKSISVNTH
GFKISPD--TII------TYNGFSNK---EIKNHYRGEVV--YNAESDIH
IPHLTVFQTLYTVARLKTPRNRIK-----GVDRDTFAKHMTEVAMATYGL
SHTADTKVGNDFVRGVSGGERKRVSIAEVSIGGSKFQCWDNATRGLDSAT

ALEFIKALKTQATITKSAATVAIYQCSKDAYDLFDKVCVLYDGYQIFFGP
SKQAKKYFQR-MGYVC-PERQTTADYLTSITSPSERIKDKDMVK-HGI--
MIPQTAYEMNQYWIQSEEYKQLQVQVNKHLDT------------------
D---SSQQREQIKNAHIAKQSKRARPSSP--YTVSFFLQVKYILIRDIWR
IKNDPSIQLFTVLSHAAMALILGSMFYEVM--LSTTTTTFYYRGAAIFFA
ILFNAFSSLL-EIFSLYETR-PITEKHKTYSLYRPSADAFA-STFSDVPT
KLATAVTFNIPYYFLINLKRDAGAFFFYFLINIITVFAMSHLFRCIGSVS
KTLPQAMVPASVLLLAFAMYTGFAIPRVQML---GWSKWISYINPLSYLF
ESLMINEFHGRNFPCAQ--YIP----SGP--NYANATGDEVTCSAL-GSI
PGNNYVSGDD-FIQTNYGYRHKNKWRSVGIGLAYIIFFLFLYLFFCEYNE
GAKQNGEMLVFPHSVVKKMKKKGIVSEKKKKNQPTLSTSDAEKDVEMNNN
SSATDSRFLRDSDAAIMGNDKTVAKEHYSSPSSSASQSHSFSKSDDIELS
KSQAIFHWKNLCY---DIPIKN-GKRRILDNVDGWVKPGTLTALIGASGA
GKTTLLDCLAERTTMGLI----T-GDVFVDGRPRDQ--SFPRSIGYCQQQ
DLH-LKTATVRESLRFSAYLRQADDVSIEEKDKYVEEVIEVLEMKLYADA
IVGI--PGEGLNVEQRKRLTIGVELAAKPKLLVFLDEPTSGLDSQTAWST
CQLMKKL-ASRGQAILCTIHQPSALLMQEFDRLLFLQEGGQTVYFGELGK
GCKTMINYFEAHGAHKCPPDANPAEWMLEIVGAAPG--------------
-------TH-AS-QDYFAIWRDSEEYREMQKELDWMERELPKRTE-GSSN
-EEQK-------EFATSTLYQIKLVSYRLFHQYWRTPFYLWSKFFSTIVS
ELFIGF-TFFK--ANTSLQGLQNQMLAIFMFTVVFNPILQQYLPLFVQQR
ELYEARERPSRTFSWKAFIVSQILVEIPWNLLAGTIAFFVYYYPVGFYRN
ASYANQLHERGALFWLFACAFYVYISSMGVLVISCIEIAENAANLASLFF
IM-SLSFCGVLATPNILPRFW-IFMYRVSPLTYLIDALLSVGLANASVVC
SSNELLKIVP--PSG-MTCSEYMEPYMQ----------STGTGYLLDGSS
----ETECHFCQFSS--TNDY--LATVSSS-YSRRWMNYGIFSAYIVFDY
CAAIFLYWLVRVPKKSKKLKK-----------------------------

>O74208

---------------MNTPDDSSVSSVDSHQPYMGFDDNVEKRIRELARS
LTQQSLTSSNRSVNKEAPAD--------------GSAPLDRVSTRASSIF
SADFKGVNPVFSDEEEDDYDARLDPNSDEFSSKAWVQNMA-KITTGDPEF
YKPYSIGCCWKDLSASGESADVSYQSTFLNLPVKLLNAVWRKA---R---
----PARESDTFRILKPMDGLLKPGELLVVLGRPGSGCTTLLKSISSTTH
GFQISKD--SVI------SYNGLTPN---EIKKHYRGEVV--YNAEADIH
LPHLTVYQTLVTVARLKTPQNRVK-----GVTREDFANHVTDVAMATYGL
SHTRDTKVGNDLVRGVSGGERKRVSIAEVWICGSKFQCWDNATRGLDSAT
ALEFVRALKTQAHIAKNVATVAIYQCSQDAYNLFNKVSVLYEGYQIYFGD
AQHAKVYFQK-MGYFC-PKRQTIPDFLTSITSPAERRI---NKEYLDKGI
KVPQTPLDMVEYWHNSEEYKQLREEIDETLAHQ-----------------
----SEDDKEEIKEAHIAKQSKRARPSSP--YVVSYMMQVKYILIRNFWR
IKNSASVTLFQVFGNSAMAFILGSMFYKIQ--KGSSADTFYFRGAAMFFA
ILFNAFSSLL-EIFSLYEAR-PITEKHRTYSLYHPSADAFA-SVISEIPP

KIVTAILFNIIFYFLVNFRRDAGRFFFYFLINVIAVFAMSHLFRCVGSLT
KTLQEAMVPASMLLLALSMYTGFAIPRTKML---GWSKWIWYINPLAYLF
ESLMVNEFHDRRFPC--NTYIP----RG--GAYNDVTGTERVCASV-GAR
PGNDYVLGDD-FLKESYDYENKHKWRGFGVGMAYVIFFFFVYLILCEFNE
GAKQKGEMLVFPHSVVKRMKKEGKIRDKTKMHTDKNDIENNSESITSNAT
NEKNML-----------------QDTYDENADSESITSGSRGGSPQVGLS
KSEAIFHWQNLCY---DVPIKT-EVRRILNNVDGWVKPGTLTALMGASGA
GKTTLLDCLAERTTMGVI-----TGDVMVNGRPRDT--SFSRSIGYCQQQ
DLH-LKTATVRESLRFSAYLRQPSSVSIEEKNEYVEAVIKILEMETYADA
VVGV--PGEGLNVEQRKRLTIGVELAAKPKLLVFLDEPTSGLDSQTAWAT
CQLMKKL-ANHGQAILCTIHQPSAMLMQEFDRLLFLQKGGQTVYFGDLGK
GCKTMIKYFEDHGAHKCPPDANPAEWMLEVVGAAPG--------------
---------SHANQDYHEVWRNSEQFKQVKQELEQMEKELSQKE------
---LDNDEDANKEFATSLWYQFQLVCVRLFQQYWRTPDYLWSKYILTIFN
QLFIGF-TFFK--ADHTLQGLQNQMLSIFMYTVIFNPLLQQYLPTFVQQR
DLYEARERPSRTFSWKAFILAQIVVEVPWNIVAGTLAYCIYYYSVGFYAN
ASQAHQLHERGALFWLFSIAFYVVYVGSLGLFVISFNEVAETAAHIGSLMF
TM-ALSFCGVMATPDAMPRFW-IFMYRVSPLTYLIDALLSTGVANVDIRC
SNTELVTFTP--PQG-LTCGQYMTPYLN----------VAGTGYLTDPSA
----TDECHFCQFSY--TNDF--LATVSSK-YYRRWRNYGIFICFIVFDY
VAGIFLYWLARVPKTNGKIAKNGKTAKVNFIRRLIPF-------------

>A6ZZ05

---------------MSSDIRDVEERNSRSSSSSSSSNSAAQSIGQHPYR
GFDSEAAERVHELARTLTSQSLLYTANSNNSSSSNHNAHNAHNADSRSVF
STDMEGVNPVFTNPDTPGYNPKLDPNSDQFSSTAWVQNMA-NICTSDPDF
YKPYSLGCVWKNLSASGDSADVSYQSTFANIVPKLLTKGLRLL---KP--
-----SKEEDTFQILKPMDGCLNPGELLVVLGRPGSGCTTLLKSISSNSH
GFKIAKD--SIV------SYNGLSSS---DIRKHYRGEVV--YNAESDIH
LPHLTVYQTLFTVARMKTPQNRIK-----GVDREAYANHVTEVAMATYGL
SHTRDTKVGNDLVRGVSGGERKRVSIAEVAICGARFQCWDNATRGLDSAT
ALEFIRALKTQADIGKTAATVAIYQCSQDAYDLFDKVCVLDDGYQLYFGP
AKDAKKYFQD-MGYYC-PPRQTTADFLTSITSPTERII---SKEFIEKGT
RVPQTPKDMAEYWLQSENYKNLIKDIDSTLEKN-----------------
----TDEARNIIRDAHHAKQAKRAPPSSP--YVVNYGMQVKYLLIRNFWR
MKQSASVTLWQVIGNSVMAFILGSMFYKVM--KKNDTSTFYFRGAAMFFA
ILFNAFSCLL-EIFSLYETR-PITEKHRTYSLYHPSADAFA-SVLSEMPP
KLITAVCFNIIFYFLVDFRRNGGVFFFYFLINVIATFTLSHLFRCVGSLT
KTLQEAMVPASMLLLAISMYTGFAIPKTKIL---GWSIWIWYINPLAYLF
ESLMINEFHDRRFPC--AQYIP----AG--PAYQNITGTQRVCSAV-GAY
PGNDYVLGDD-FLKESYDYEHKHKWRGFGIGMAYVVFFFFVYLILCEYNE
GAKQKGEMVVFLRSKIKQLKKEGKLQEKHRPGDIENNAGSSPDSATTEKK
ILDDSS-----------------------------EGSDSSSDNAGLGLS

```
KSEAIFHWRDLCY---DVPIKG-GQRRILNNVDGWVKPGTLTALMGASGA
GKTTLLDCLAERVTMGVI-----TGNIFVDGRLRDE--SFPRSIGYCQQQ
DLH-LKTATVRESLRFSAYLRQPSSVSIEEKNRYVEEVIKILEMQKYSDA
VVGV--AGEGLNVEQRKRLTIGVELAARPKLLVFLDEPTSGLDSQTAWDT
CQLMRKL-ATHGQAILCTIHQPSAILMQQFDRLLFLQKGGQTVYFGDLGE
GCKTMIDYFESKGAHKCPPDANPAEWMLEVVGAAPG--------------
---------SHATQDYNEVWRNSDEYKAVQEELDWMEKNLPGRS------
---KEPTAEEHKPFAASLYYQFKMVTIRLFQQYWRSPDYLWSKFILTIFN
QVFIGF-TFFK--ADRSLQGLQNQMLSIFMYTVIFNPILQQYLPSFVQQR
DLYEARERPSRTFSWLAFFLSQIIVEIPWNILAGTIAYCIYYYAVGFYAN
ASAAGQLHERGALFWLFSIAFYVYIGSMGLLMISFNEVAETAAHMGTLLF
TM-ALSFCGVMATPKAMPRFW-IFMYRVSPLTYMIDALLALGVANVDVKC
SNYEMVKFTP--PSG-TTCGDYMASYIK----------LAGTGYLSDPSA
----TDICSFCAVST--TNAF--LATFSSH-YYRRWRNYGIFICYIAFDY
IAATFLYWLSRVPKNNGKISEKPKK-------------------------
--------------------------------------------------
--------------------------------------------------
--------------------------------------------------
----------------------------
>P33302
--------------------------------------------------
--------------------------------------------------
--------------------------------------------------
--------------------------------------------------
--------------------------------------------------
--------------------------------------------------
--------------------------------------------------
--------------------------------------------------
-----------------MPEAKLNNNVNDVTSYSSASSSTENAADLHNYN
GFDEHTEARIQKLARTLTAQ-----------SMQNSTQSAPNKSDAQSIF
SSGVEGVNPIFSDPEAPGYDPKLDPNSENFSSAAWVKNMA-HLSAADPDF
YKPYSLGCAWKNLSASGASADVAYQSTVVNIPYKILKSGLRKF---QR--
-----SKETNTFQILKPMDGCLNPGELLVVLGRPGSGCTTLLKSISSNTH
GFDLGAD--TKI------SYSGYSGD---DIKKHFRGEVV--YNAEADVH
LPHLTVFETLVTVARLKTPQNRIK-----GVDRESYANHLAEVAMATYGL
SHTRNTKVGNDIVRGVSGGERKRVSIAEVSICGSKFQCWDNATRGLDSAT
ALEFIRALKTQADISNTSATVAIYQCSQDAYDLFNKVCVLDDGYQIYYGP
ADKAKKYFED-MGYVC-PSRQTTADFLTSVTSPSERTL---NKDMLKKGI
HIPQTPKEMNDYWVKSPNYKELMKEVDQRLLND-----------------
----DEASREAIKEAHIAKQSKRARPSSP--YTVSYMMQVKYLLIRNMWR
LRNNIGFTLFMILGNCSMALILGSMFFKIM--KKGDTSTFYFRGSAMFFA
ILFNAFSSLL-EIFSLYEAR-PITEKHRTYSLYHPSADAFA-SVLSEIPS
KLIIAVCFNIIFYFLVDFRRNGGVFFFYLLINIVAVFSMSHLFRCVGSLT
KTLSEAMVPASMLLLALSMYTGFAIPKKKIL---RWSKWIWYINPLAYLF
ESLLINEFHGIKFPC--AEYVP----RG--PAYANISSTESVCTVV-GAV
PGQDYVLGDD-FIRGTYQYYHKDKWRGFGIGMAYVVFFFFVYLFLCEYNE
GAKQKGEILVFPRSIVKRMKKRGVLTEKN-----ANDPENVGERSDLSSD
RKMLQE-----------------------------SSEEESDTYGEIGLS
KSEAIFHWRNLCY---EVQIKA-ETRRILNNVDGWVKPGTLTALMGASGA
GKTTLLDCLAERVTMGVI-----TGDILVNGIPRDK--SFPRSIGYCQQQ
DLH-LKTATVRESLRFSAYLRQPAEVSIEEKNRYVEEVIKILEMEKYADA
VVGV--AGEGLNVEQRKRLTIGVELTAKPKLLVFLDEPTSGLDSQTAWSI
CQLMKKL-ANHGQAILCTIHQPSAILMQEFDRLLFMQRGGKTVYFGDLGE
GCKTMIDYFESHGAHKCPADANPAEWMLEVVGAAPG--------------
```

---------SHANQDYYEVWRNSEEYRAVQSELDWMERELPKKG------
---SITAAEDKHEFSQSIIYQTKLVSIRLFQQYWRSPDYLWSKFILTIFN
QLFIGF-TFFK--AGTSLQGLQNQMLAVFMFTVIFNPILQQYLPSFVQQR
DLYEARERPSRTFSWISFIFAQIFVEVPWNILAGTIAYFIYYYPIGFYSN
ASAAGQLHERGALFWLFSCAFYVYVGSMGLLVISFNQVAESAANLASLLF
TM-SLSFCGVMTTPSAMPRFW-IFMYRVSPLTYFIQALLAVGVANVDVKC
ADYELLEFTP--PSG-MTCGQYMEPYLQ----------LAKTGYLTDENA
----TDTCSFCQIST--TNDY--LANVNSF-YSERWRNYGIFICYIAFNY
IAGVFFYWLARVPKKNGKLSKK----------------------------

>Q9UVV5

--------------------MSLASDKKDADVASTTTTAQDDDNLSTYH
GFDHHVQDQVRQLARTLTQQ--------------------SSLHQKKEH
TLPEEGINPIFTNTEADDYNPRLDPTSDEFSSAEWVQNMS-NISNSDPDY
YKPYSLGCYWKDLVATGESADIEYQANFLNGPYKGLKTVYNTVVPSTA--
-----SSKDKNFKILKSMEGAVNPGELLVVLGRPGSGCTTLLKSISSNTH
GFNIAKD--STI------SYSGMTPN---DIRKHFRGEVV--YNAEADIH
LPHLTVYQTLLTVARLKTPQNRLK-----GIDRETYARHLTEVAMATFGL
SHTRNTKVGNDLVRGVSGGERKRVSIAEVSICGSKFQCWDNATRGLDSAT
ALEFIRALKVQASISNAAATVAIYQCSQDAYDLFDKVCVLYDGYQIYLGP
AGKAKRYFQK-MGYVS-PERQTTADFLTAVTSPSERII---NQDYINRGI
FVPQTPKEMWEYWRASEDHADLIKEIDSKLSDN-----------------
----YDANLAEIKDAHVARQSKRARPSSP--YTVSYGMQIKYLLIRNFWR
IKQSSGVTLFMVIGNSSMAFILGSMFYKVM--KHNTTSTFYFRGAAMFFA
VLFNAFSSLL-EIFSLFEAR-PITEKHRTYSLYHPSADAFA-SILSEVPA
KLITAVCFNIIYYFLVNFRRNGGVFFFYFLINIVAVFAMSHLFRCVGSVS
KTLSAAMVPASMLLLGLSMYSGFAIPRTKIL---GWSKWIWYINPLAYLF
ESLMINEFHDRKFPC--SQYIP----SG--SVYNNVPADSRICSSV-GAI
RGNDYVLGDD-FLRESYSYLHKHKWRGFGIGLAYVIFFLVLYLILCEYNE
GAKQKGEILVFPQNIVRRMKKERKLKNV------SSDNDVEIGDVSDISD
KKILAD----------------------------SSDESEESGANIGLS
QSEAIFHWRNLCY---DVQIKK-ETRRILNNVDGWVKPGTLTALMGASGA
GKTTLLDCLAERVTMGVI-----TGEVSVDGKQRDD--SFARSIGYCQQQ
DLH-LKTSTVRESLRFSAYLRQPADVSIEEKNQYVEDVIKILEMEQYADA
VVGV--PGEGLNVEQRKRLTIGVELAAKPKLLVFLDEPTSGLDSQTAWSI
CQLMKKL-ANHGQAILCTIHQPSAILMQEFDRLLFLQRGGKTVYFGDLGD
GCKTMIDYFESHGSHKCPPDANPAEWMLEVVGAAPG--------------
---------SHANQDYHEVWRNSDEYQKVQEELEWMSNELPKKN------
---TNNSETVHKEFATGVLYQCKLVSPRLFQQYWRSPDYLWSKFFLTIFN
NIFIGF-TFFK--ADRSLQGLQNQMLAVFMFTVIFNPLLQQYLPSFVQQR
DLYEARERPSRTFSWKAFIVSQILVEIPWNILAGTVAFVIYYYAIGFYSN
ASVAHQLHERGALFWLFSCAFYVYIGSLALFCISFNQVAEAAANMASLMF
TL-SLSFCGVLVTPNGMPRFW-IFMYRVSPLTYLIDGMLSTGVANVAIKC

SNYELLRFSP--AAN-LTCGEYLGPYLQ----------TVKTGYIVDPSA
----TDTCELCPYSH--TNDF--LSSVSSK-YSRRWRNWGIFICYIAFNY
IAGIFLYWLARVPKKSGKLAKK----------------------------

>Q96WM8

--------------------MDADSTSSLVEYQGFDHQVQEQIRDLART
LSRNSLKEQASQQESTGNVL------------------ASDDDGAKSIF
STRYEGVNPVFTDSDASGYDVRLDPNSDEFSSAAWIKNMV-ALANNDPEY
YKHYTIGCCWKDLRAFGDSTDVAYQSTVLNLPGKIFSSVKRHF-------
---VKSKPEDVFDILKPMDGLLKPGDLLVVLGRPGSGCTTLLKTISSNID
GYNVDEN--SVI------SYNGLDPR---TIKKHFRGEVV--YNAESDVH
FPHLSVYETLYNIALLVTPSNRIK-----GATREEFANHVTQVAMATYGL
SHTRDTKVGNELVRGVSGGERKRVSIAEVTICGSRFQCWDNATRGLDSAT
ALEFIRALKTSTDISGSTGVIAIYQCSQDAYDLFDKVCVLHEGYQIFYGN
AKAAKAYFER-MGYVS-PSRQTTADFLTAVTNPAERIVNQEFVK-EGR--
FIPSTAKQMEEYWRNSPEYKQLRGEIEEELNK------------------
D---STQTRQELIEAHIARQSKRQRKESP--YIVNYGMQVKYLTMRNFLR
IKKSYGITVGTIVGNTAMSLVLGSIFYKSM--KDTTTNTFFYRGAAMFIA
VLFNSFSSML-EIFSLYEAR-PIIEKHKRYSLYHPSADALA-SMLSELPA
KIITAICFNLILYFMVNFRREAGPFFFYFLMNFLATLVMSAIFRCVGSAT
KTLSEAMVPASCLLLAISLYVGFSIPKKNLL---GWSRWIWYINPLSYIF
ESLMINEFNGRDFPCAA--YIP----SGS--GYENIGLYERVCNTV-ASQ
PGLSYVSGRA-FIEEAYGYNPSHRWRALGIALAYFIFFTAFYLLFCEFNE
SAVQKGEILLFPKSVLKRAKKQKLIKAKHDVEAVQDSEGALTDQKLLQDS
LVESNI------------------SSSSDKSVNV---------------GLS
KSEAIFHWRNVCY---DVQIKK-ETRRILSNVDGWVKPGTLTALMGSSGA
GKTTLLDCLASRVTMGVI----T-GDMFVNGHLRDN--SFPRSIGYCQQQ
DLH-LSTSTVRESLRFSAYLRQPSSVSIEEKNNYVEDVINILEMQQYADA
VVGV--AGEGLNVEQRKRLTIGVELAAKPKLLLFLDEPTSGLDSQTAWSV
CQLMRKL-ADHGQAILCTIHQPSALLMQEFDILLFLQKGGKTVYFGNLGE
GCQEMINYFEKHGASKCPEGANPAEWMLDVIGAAPG--------------
-------SH-AT-QDYHEVWRNSDEYQAVQKELDWMESELRKKPL-DTSS
-E-QS-------EFGTSLFYQYKVVTLRLFEQYYRTPSYIWSKLFLTIFS
QLFIGF-TFFK--ANLSIQGLQNQLFAIFTFTVIFNPACQQYLPLFVSQR
DLYEARERPSRTFSWLAFIFSQITVEIPLNICFGTIAFFVFYYPIGFYNN
ASYAGQLNERGVLFWLFSVSFYVFISSMGQLCIAGLQYAEAAGNMASLMF
TM-SLNFCGVFGGSGVLPGFW-IFMYRISPLTYFIDGVLSTGLANNPVTC
ANYEYVSFNP--RSG-ETCGEYMADYID----------KNG-GYLLDSNA
----TEDCNFCKISE--TNAF--LTSFQSS-YHRRWRNFGIFIVFIVFNW
CGCVFLYWLARVPKKKNRVADERDPD------------------------
---HGKKI
ENVQKEKVEPQSNEQV----------------------------------

>P32568

----------------------------MSNIKSTQDSSHNAVARSSSAS
FAASEESFTGITHDKDEQSDTPADKLTKMLTGPARDTASQISATVSEMAP
DVVSKVESFADALSRHTTRSGAFNMDSDSDDGFDAHAIFESFVRDADEQG
IHIRKAGVTIEDVSAKGVDASALEGATFGNILCLPLTIFKGIKA-KRHQK
----------MRQIISNVNALAEAGEMILVLGRPGAGCSSFLKVTAGEID
QFAGGVS--GEV------AYDGIPQE---EMMKRYKADVI--YNGELDVH
FPYLTVKQTLDFAIACKTPALRVN-----NVSKKEYIASRRDLYATIFGL
RHTYNTKVGNDFVRGVSGGERKRVSIAEALAAKGSIYCWDNATRGLDAST
ALEYAKAIRIMTNLLKSTAFVTIYQASENIYETFDKVTVLYSGKQIYFGL
IHEAKPYFAK-MGYLC-PPRQATAEFLTALTDPNGFHL--IKPG-YEN--
KVPRTAEEFETYWLNSPEFAQMKKDIAAYKEKV-----------------
---NTEKTKEVYDESMAQEKSKYTRKKSY--YTVSYWEQVKLCTQRGFQR
IYGNKSYTVINVCSAIIQSFITGSLFYNT----PSSTSGAFSRGGVLYFA
LLYYSLMGLA-NIS--FEHR-PILQKHKGYSLYHPSAEAIG-STLASFPF
RMIGLTCFFIILFFLSGLHRTAGSFFTIYLFLTMCSEAINGLFEMVSSVC
DTLSQANSISGILMMSISMYSTYMIQLPSMH---PWFKWISYVLPIRYAF
ESMLNAEFHGRHMDCA-NTLVP----SG--GDYDNLSDDYKVCAFV-GSK
PGQSYVLGDD-YLKNQFQYVYKHTWRNFGILWCFLLGYVVLKVIFTEYKR
PVKGGGDALIFKKGSKRFIAHADEESPDNVNDIDAKEQFSSESSGANDEV
FDDLE---
-AKGVFIWKDVCF---TIPYEG-GKRMLLDNVSGYCIPGTMTALMGESGA
GKTTLLNTLAQR-NVGII-----TGDMLVNGRPIDA--SFERRTGYVQQQ
DIH-IAELTVRESLQFSARMRRPQHLPDSEKMDYVEKIIRVLGMEEYAEA
LVGE--VGCGLNVEQRKKLSIGVELVAKPDLLLFLDEPTSGLDSQSSWAI
IQLLRKL-SKAGQSILCTIHQPSATLFEEFDRLLLLRKGGQTVYFGDIGK
NSATILNYFERNGARKCDSSENPAEYILEAIGAGAT--------------
---------ASVKEDWHEKWLNSVEFEQTKEKVQDLINDLSKQET-----
---KSEVGDKPSKYATSYAYQFRYVLIRTSTSFWRSLNYIMSKMMLMLVG
GLYIGF-TFFN--VGKSYVGLQNAMFAAFISIILSAPAMNQIQGRAIASR
ELFEVRESQSNMFHWSLVLITQYLSELPYHLFFSTIFFVSSYFPLRIFFE
A------SRSAVYFLNYCIMFQLYYVGLGLMILYMSPNLPSANVILGLCL
SF-MLSFCGVTQPVSLMPGFW-TFMWKASPYTYFVQNLVGIMLHKKPVVC
KKKELNYFNP--PNG-STCGEYMKPFLE-----------KATGYIENPDA
----TSDCAYCIYEV--GDNY--LTHISSK-YSYLWRNFGIFWIYIFFNI
IAMVCVYYLFHVRQSSFLSPVSILNKIKNIRKKKQ---------------

>A7TJY8

---------------------------------MADNISEINEKLGDLNN
SRANNNGATNDESEETLEPEHILENFDNTGRSFAKDSRPSSISEHESTES
LTNSRLVNRITSLARTFSHVETSSSEADFETIKDPRKIIEKFVRDAFEQG
IHIRNTSIIMEDITVQVPDTSRQEASTFGDFILLPITIYKMIKKFTQHKK
----------MKSILSEVNFLVKPGNMVLVLGRPGSGCSTLLKTAVGETS
SYKGNVS--GSV------TYDGIPQK---EMVKNYKSDLI--YCAETDIH
FPHLTVKQTLDFALACKVQNVRIN-----NMSAKKQIGIARDLLATIFGL
RHTYSTKVGNDFVRGVSGGERKRVSIAEALAARGTIYAWDNATRGLDAST
ALEFAQALRTFTELLKSTSFCCLYQVSENIFHTFDMVTVLYSGKQIYYGP
VAKAKDFFER-MGYKC-PPRQDTAEFLTALTDPNGLHL--IKEG-FEG--
TVPRSADEFESYWKSSPEFKQLLSDIETYKTEV-----------------
---DPEKTREIYTMSLKQEKTKWTRKSSY--YTVSFPEQVKLCTIRGFQR
IYGDKSFSIISTVAAVFQSFIIGSLFYNT----PSSTSGAFSRGGTHFFM
LLYYSLISLA-NIS--FAQR-PIINKHKSYSFYHPAAEALS-ENLSFFPF
RMIGLTMFMVVIYFLAGLRRTASAFFITYLFLTMAAEIINCLFQMIAAAC
ETINEANSINGILMLSLSMYSTYMIQLPEMR---VYFKWISFALPLRYGF
ESMLNAEFHNRYMSCG-GTLVP----SG--PGYENVPPSNQVCAFP-GST
PGSSEVLGDN-YLRVQFTYQYKNTWRNFGIFWCFLLGYLGLKCLLAEFKP
SFRKEGNILLYKKNSKILSPFKDEESTPDVGEVSLKHHTTATSSNNETIV
HEKDEV--FEGL
GSTGNFLWRDLVY---TIPLEG-STRVLLDHVSGYCIPGKLTALMGESGA
GKTTLLNTLAQRNEIGVV-----TGDIKVDGAKIDI--SFGRRTGYVQQQ
DVH-LAELTVREALIFSAMMRRPESVPYSEKLDFVEKIIEVLEMGDYADA
LVGL--EGSGLNVEQRKKLSIGVELVARPDILLFVDEPTSGLDSQEAWSI
VQLLKRL-SESGQSILCTIHQPSATLFEQFDRLLLLKKGGKTVYFGDIGN
NSDTVLDYFERNGGRKCLESENPAEYILEIIGAGAS--------------
---------ASVEEDWGSVWNNSPESTTVQNDIEGLLSEKGNRP------
----DGEHSINKTYATSYFYQFKCVQMRAFTILWRDTNYLVSKFMLFLVT
GLYIGF-TFFH--VPQSYNGLQGALFAAFLTLITSAPVMNQIQARAIEAR
ELYEVRESKSNVFHWSLLLLCQYFSEIPYSLVFSAIYFVSFYFPVGIFYE
A------SRSAVFYLNYGVMFQFFYVALGLMVLYLSPNLQSANVIMGLTL
SF-LVAFCGVVQPKTLMPGFW-TFMWKASPYTYFVQNLISIMLHDKPVVC
KEKELSFLDP--PTG-LTCGEYMAPFLT-----------IAPGYINNPDS
----TSNCGYCLYSV--GDSY--LERVSAS-YSNLWRNFGFYWAYIVFNL
IAMVVSYYIFHILKIPSKIIHIIKNALAAFRIKQKNKNNN----------

>B9WJ85

--MSSEDIES
SSSSLQEYVGQQHHKTQPSTDEDYNDYDENNDESRRLHLVRTVSSINRHN

FDEKFDSISREISRQVTNKEGEFQLRLDEFNLAKILANFV---YFAKKQG
IVLRKSGITFQDLCVYGVDESFAIAPTVTDLLKGPIGGIQAILSQMKTPP
-----------RKILKNLNGFAKPGESVLVLGRPGAGCTTFLKALSGTDF
DLYKGVT--GDI------RYDGLPQS---EMLKLFKNDLV--YNPELDVH
FPHLTVDQTLTFAIACKTPEMRIN-----GVTRDEFINAKKEILATVFGL
RHTYNTKVGNDFVRGVSGGERKRVSIAEALACNGSIYCWDNATRGLDAST
ALEFAQAIRTSTKLLKTTAFVTIYQAGEGIYETFDRVTVLYDGHQIYYGP
ANKAKKYFED-MGWEC-PPRQSTAEFLTAITDPIGRFP---RAG-WEN--
KVPRTAQDFEHYWLNSPQYQELMQEIKDYNDEI-----------------
---DEDETRGKYYESIQQEKMKGARTKSP--FTISYLEQLKLCFIRSYQR
ILGDSAYTLTLMFASVAQAFVAGSLYYNT----PDDVSGAFSRGGVIFFA
VLFMSLMGLA-EISASFSSR-PILMKQKNYTMYHPSADSLS-NFVMSIPI
SIFINTFFVIILYFLSNLARDAGKFFICYLFVIMLHLTMKSMFQAIAAIN
KSIAGANAMGGISVLASLMYSSYMIQRPSMH---PWFKWISYINPVLYAF
EAVIASEFHGRKMQCTSQYLTP----SG--PGYENLGAGEQVCTFI-GSV
PGQSWVLGDD-YLRIAYTYRFSHVWRNLGILFGFLAFFLTIATLGTEYVK
PITGGGDKLLFLKGKVPEHITLPSERKEEDIESGGDTTATSNGTLS-QGK
SDD-EK------------------------------------GAIVDEGL
KAKGVFVWKDVDY---VIPYEG-KKRQLLQNVSGYCVPGTLTALMGESGA
GKTTLLNVLAQRIDFGVI-----TGDMLVNGRPLDT--SFSRRTGYVQQQ
DIH-FSEVTVRESLQFAARLRRSNDVSDAEKLEYVEKIIDVLDMRGYADA
VVGR--LGNGLNVEQRKKLSIGVELVAKPSLLLFLDEPTSGLDSQSAWAI
VKLLRDL-ANAGQSILCTIHQPSATLFEEFDRLLLLKKGGIVTYFGDIGP
RSRTILDYFERNGARHCDDKENPAEYILEAIGAGAT--------------
---------ASTEFDWGDIWAQSPEKVQTDAKRDELIKESAQNAA--DTT
TSSSEKNS--TSKYATPYWYQFRHVTHRTSLIFYRDPDYIAAKIFLMTIA
GLFIGF-TFFG--LKHTKTGAQNGMFCAFLSCVIAAPLINQMLE-KAASR
DIYEVREKLSNTYHWSLLILPQVIFEVIYMIIGGTIMFVCLYFPTQVNTV
A------SHSGIFYFSQAIFLQTFAVSFGLMVSYVSPDVESASVIVSFLY
TF-IVSFSGVVQPVDLMPGFW-TFMNKVSPYTYFIQNLVSSFLHDRTIRC
NAKELSYFNP--PSG-ETCKEFASAFIS-----------RHGGYLIDDGA
----TSNCGYCSFSN--ADEY--LLTIGVK-FAYRWRNIGFFCAYIIFNI
SICLILYYFIRYRKISLNVTGLISKFKKSKI-------------------

>Q5A8I9

--MSSEDIGS
SSSSLQEYVGQQQHNKIQPSTD--DDYNEDDYESRRLHLVRTVSSINHHN
FDEKFDTISREISRQVTNKEGEFQLRLDEFNLAKILANFV---YFAKKQG
IVLRKSGITFQDLCVYGVDESFAIAPTVTDLLKGPVGAVQAILSQMKTPP
-----------RKILKNLNGFAKPGESVLVLGRPGAGCTTFLKALSGTDF
DLYKGVT--GDI------RYDGLPQK---EMLKLFKNDLV--YNPELDVH
FPHLTVDQTLTFAIACKTPEMRIN-----GVTRDEFINAKKEILATVFGL
RHTYHTKVGNDFVRGVSGGERKRVSIAEALACNGSIYCWDNATRGLDAST

ALEFAQAIRTSTKLLKTTAFVTIYQAGEGIYETFDRVTVLYDGHQVYYGP
ANKAKKYFED-MGWEC-PPRQSTAEFLTAITDPIGRFP---RAG-WEN--
KVPRTAQDFEHYWLNSPQYQELMQEIKDYNDEI-----------------
---DEDETRSKYYQSIQQEKMKGSRTKSP--FTISYLEQLKLCFIRSYQR
ILGDSAYTITLMFASVAQAFVAGSLYYNT----PDDVSGAFSRGGVIFFA
VLFMSLMGLA-EISASFSSR-PILMKQKNYTMYHPSADSLS-NFVMSIPI
SIFINTFFVIILYFLSNLARDAGKFFICYLFVIMLHLTMKSMFQAIAAIN
KSIAGANAMGGILMLASLMYSSYMIQRPSMH---PWFKWISYINPVLYAF
EAVIASEFHGRKMQCTSQYLTP----SG--PGYENLGAGEQVCTFI-GSV
PGQSWVLGDD-YLRIAYTYRFSHVWRNLGILFGFLAFFLAIATLGTEYVK
PITGGGDKLLFLKGKVPEHITLPSEKKEEDIESGGNSDTTATSNGTLSQG
KSE-EK------------------------------------AAIADDGL
KAKGVFVWKDVDY---VIPYEG-KKRQLLQNVSGYCVPGTLTALMGESGA
GKTTLLNVLAQRVDFGVI-----TGDMLVNGRPLDT--SFSRRTGYVQQQ
DIH-FSEVTVRESLQFAARLRRSNDVSDAEKLEYVEKIIDVLDMRGYADA
VVGR--LGNGLNVEQRKKLSIGVELVAKPSLLLFLDEPTSGLDSQSAWAI
VKLLRDL-ANAGQSILCTIHQPSATLFEEFDRLLLLKKGGIVTYFGDIGP
RSRTILDYFERNGARHCDDKENPAEYILEAIGAGAT--------------
---------ASTDFDWGEIWAQSPEKVQTDAKRDELINESAKNAT--DTS
ATDSPSEKNLTSKYATPYWYQFRHVTHRTSLIFYRDPDYIAAKVFLMTIA
GLFIGF-TFFG--LKHTKTGAQNGMFCAFLSCVIAAPLINQMLE-KAGSR
DIYEVREKLSNTYHWSLLILPQIIFEVIYMIIGGTIMFVCLYFPTQVSTV
A------SHSGMFYFSQAIFLQTFAVSFGLMVSYVSPDIESASVIVSFLY
TF-IVSFSGVVQPVNLMPGFW-TFMNKVSPYTYFIQNLVSSFLHDRTIRC
NAKELSYFNP--PSG-QTCKEFASAFIS-----------RNGGYLVDEGA
----TSNCGYCNFSN--ADQY--LLTIGAK-FSYRWRNIGFFCVYIIFNI
SVCLVLYYFLRYRKVSFNVTGLVNKFKKSKK-------------------

>C5MHE4

--MVQDDQSS
NSYSIEEYGADYFGQQGSSGLH--DESTNIDEETRRASLVRTITAIEANE
YQQKFDSISREISRQITNKEGEFQLKLEEFNLVKILANFV---YFAKKQG
INLRKSGVTFKDLSVFGVDDSVAVVPTVLDVLKGPVYGIQELIRKIKTPK
-----------REILKSFNGLAKPGDMVLVLGRPGAGCTTFLKALSGTDF
DLYKGIE--GDI------RYDGLPQN---EMIKMFRNDLI--YNPELDIH
FPHLTVDQTLSFAIACKTPNIRIN-----GVTREQFINAKKEVLATVFGL
RHTYHTKVGNDYVRGVSGGERKRVSIAEALACHGSIYCWDNATRGLDSST
ALEFAQAIRTSTKLLGTTAFVTIYQAGENIYEKFDKVTILYDGHQIYYGP
ANKAKKYFEN-MGWEC-PPRQSTAEFLTAVTDPIGRFP---KKG-WED--
KVPRTAEDFESRWLNSPQYNELLNEIDEYNSQI-----------------
---DEDQVRRDYYDSVIQEKMKGARKKSP--FTVSYMQQLKLCFIRSFYR
IKGDNAYTITLVGAAVCQAFIAGSLYYNT----PNDVSGAFSRGGVIFFA
VLFMSLMGLA-EISASFRNR-LILNKQKNYSMYHPSADALS-QFVMAIPI

SLFVNALFVVILYFLSNLAVDAGKFFTCYLFVFMLHLTMGAMFQAVAALH
KTIAGANAVGGILVLATLSYSSYMIQRPTMH---GYSRWISYINPVLYAF
EAIIASEFHHRKMECTSEYLTP----SG--PGYENVGEGEQVCAFT-GSI
PGTKWVSGEK-YLSVSYTYKFIHVWRNFAILVGFLAFFLAVNALGTEFIK
PITGGGDKLLYLRGKVPDHVALPEEKQNGDIESAGQRSGSTQLEKPFSSK
EDTLGQ------------------------------------CEKKDATL
ATNDIYVWKDVDY---IIPYEG-KQRQLLNCVSGFCIPGTMTALMGESGA
GKTTLLNVLAQRIDFGTI-----TGDMLVNGRPLDS--SFSRRTGYVQQQ
DIH-CEEVTVRESLQFAARLRRSNDVSDEEKLDYVEKIIDVLDMKPYADA
IVGR--LGNGLNVEQRKKLSIGVELVAKPSLLLFLDEPTSGLDSQSAWAI
VKLLRTL-ANSGQSILCTIHQPSATLFEEFDRLLLLKKGGIVTYFGDIGP
RSHILLNYFESNGARHCGDDENPAEYILEAIGAGAT--------------
---------ASSNFDWGEIWAASPQKMDTEKKRDELIEESSKKPV--GTG
SEKEDKKL--HQKYATPYWYQFRITLQRSNTVLWRIPGYCVSKILVMTLS
GLFIGLVTFFS--LQQTYAGSRNGMFCGFLSVVVVAPIANMLMERYSYAR
AIFEARESLSNTYHWSLLVISSMIPEIPYLIVGGTFFFITVYFPATRSAG
S-------QAGIFYFTQGVFLQFFTITFAAMILFIAPDLESASVIFSFLY
TF-IVAFSGIVQPTNLMPGFW-TFMYKASPYTYFISNLVSSFLHGRKIRC
TEEELAVFNP--PAG-QTCQEYTAAFLS-----------RKPGYLVDGSA
----TSNCSYCPYST--ADEY--LASIKAK-YHYRWRNIGFFCAYILFNI
GFCLFLYYFIRYKRIFKGVPALFHNILRMFKKKKA---------------

>Q02785

-------------------------------MSSTDEHIEKDISSRSNHD
DDYANSVQSYAASEGQVDNED----------LAATSQLSRHLSNILSNEE
GIERLESMARVISHKTKKEMDSFEINDLDFDLRSLLHYLR-S--RQLEQG
IEPGDSGIAFKNLTAVGVDASAAYGPSVEEMFRNIASIPAHLISKF----
---TKKSDVPLRNIIQNCTGVVESGEMLFVVGRPGAGCSTFLKCLSGETS
EL-VDVQ--GEF------SYDGLDQS---EMMSKYKGYVI--YCPELDFH
FPKITVKETIDFALKCKTPRVRID-----KMTRKQYVDNIRDMWCTVFGL
RHTYATKVGNDFVRGVSGGERKRVSLVEAQAMNASIYSWDNATRGLDAST
ALEFAQAIRTATNMVNNSAIVAIYQAGENIYELFDKTTVLYNGRQIYFGP
ADKAVGYFQR-MGWVK-PNRMTSAEFLTSVTVDFENRTLDIKPG-YED--
KVPKSSSEFEEYWLNSEDYQELLRTYDDYQSRH-----------------
P---VNETRDRLDVAKKQRLQQGQRENSQ--YVVNYWTQVYYCMIRGFQR
VKGDSTYTKVYLSSFLIKALIIGSMFHKIDDKSQSTTAGAYSRGGMLFYV
LLFASVTSLA-EIGNSFSSR-PVIVKHKSYSMYHLSAESLQ-EIITEFPT
KFVAIVILCLITYWIPFMKYEAGAFFQYILYLLTVQQCTSFIFKFVATMS
KSGVDAHAVGGLWVLMLCVYAGFVLPIGEMH---HWIRWLHFINPLTYAF
ESLVSTEFHHREMLCSA--LVP----SGP--GYEGISIANQVCDAA-GAV
KGNLYVSGDS-YILHQYHFAYKHAWRNWGVNIVWTFGYIVFNVILSEYLK
PVEGGGDLLLYKRGHMPE-----LGTENADARTASREEMMEALNGPNVDL
EKVIA---

-EKDVFTWNHLDY---TIPYDG-ATRKLLSDVFGYVKPGKMTALMGESGA
GKTTLLNVLAQRINMGVI----T-GDMLVNAKPLPA--SFNRSCGYVAQA
DNH-MAELSVRESLRFAAELRQQSSVPLEEKYEYVEKIITLLGMQNYAEA
LVGK--TGRGLNVEQRKKLSIGVELVAKPSLLLFLDEPTSGLDSQSAWSI
VQFMRAL-ADSGQSILCTIHQPSATLFEQFDRLLLLKKGGKMVYFGDIGP
NSETLLKYFERQSGMKCGVSENPAEYILNCIGAGAT--------------
-------AS-VN-SDWHDLWLASPECAAARAEVEELHRTLPGRAV-NDDP
-ELAT-------RFAASYMTQIKCVLRRTALQFWRSPVYIRAKFFECVAC
ALFVGL-SYVG--VNHSVGGAIEAFSSIFMLLLIALAMINQLHVFAYDSR
ELYEVREAASNTFHWSVLLLCHAAVENFWSTLCQFMCFICYYWPAQFSGR
ASHA------GFFFFFYVLIFPLYFVTYGLWILYMSPDVPSASMINSNLF
AA-MLLFCGILQPREKMPAFWRRLMYNVSPFTYVVQALVTPLVHNKKVVC
NPHEYNIMDP--PSG-KTCGEFLSTYMD----------NNT-GYLVNPTA
----TENCQYCPYTV--QDQV--VAKYNVK-WDHRWRNFGFMWAYICFNI
AAMLICYYVVRVKVWSLKSVLNFKKW------------------------
---FNGPR
KERHEKDTNIFQTVPGDENKITKK--------------------------

>Q8X0Z3

--MSATGVPAELNNLGAPITATTQNPSGLANSQVTSGPASSATQHDEH--

------------RSSAGNTLADEENDKAVESEKAEVIDAAADGKQKRLAA
DSSENIVTELEPHHVSIHRGKEEFAALERRYSTLSQQSQHELHRPTTRHS
VRSSFSRKDRVVSRLTQDDAEKAKEGEGEFNLVDVLRSGR---ENQDEAG
IKRKAVGVIWEDLEVIGAGGMRINIRNFSSAIIEQFMMPAFKILSIFGVN
PFAPKP-----KAILHPSSGLLKPGEMCLVLGRPGAGCTTFLKTITNQRA
GFMEIK---GNV------EYAGVGWK---EMRKRYGGEVV--YNQEDDDH
LPTLTVAQTIRFAPATKTPKKKIP-----GVSAKQFQDDMLDLLLSMLNI
KHTANTIVGNAYVRGVSGGERKRVSIAEMFCSGATVCSWDNSTRGLDAST
ALDYAKSLRLLTDIMGQTTFVSLYQAGEGIYDQFDKVLVLNEGHVAYFGP
AKEARQYMIG-LGYRD-LPRQTTADYLSGCTDVNERRF---ADGRDET--
NVPATPEEMDKAYKESEVCARMTREREEYKQLM-----------------
--AEDATAREDFRQAVLEQKHKGVSKKSP--YTVSFLQQVFIIFKRQLRL
KFQDHFGISTGYATAIISALIVGSVYFRL----PETASGAFTRGGLLFLG
LLFNALTSFS-ELPSQMLGR-SVLYRQNEYRFYRPAAFALA-AVLADVPY
NASVIFLFSIVLYFMGGLYSSGGAFFMFFLFVFLTFMVMSAFFRTLGVAT
SDYNVAARLASVLISFMVTYTGYMIPVQRMK---RWLFWIFYLNPLSYGY
EAIFANEFSRINLTCDSSYTIPRNIPEAGITGYPDTLGPNQMCSIF-GST
PGDPNVSGSD-YMATGYSYYKAHIWRNFGILLGFFAFFMFLQMLFIEVLE
QGAKHFSINVYKKEDKDLKAKNERLAERLEAFRAGELEQDLSELK-----

MRPEPFTWEVLNY---TVPVPG-GHRQLLNDIYGYVKPGSLTALMGASGA
GKTTVLDVRASRKNIGVI-----EGDVLMNGRPIGT--GFQRGCGYAEQQ
DTH-EWTTTVREALRYSAYLRQPQHVPKQEKDDYVEDIIELLELQELADA
MIGF--PGYGLSVEARKRVTIGVELAAKPELLLFLDEPTSGLDGQSAYNI
VRFLKKL-CAAGQKILCTIHQPNALLFQSFDRLLLLQRGGECVYFGDIGP
DSKVLIDYLERNGA-KVPHDANPAEFMLEAIGAGSR--------------

-------KR-IG-SDWGEKWRNSPEFAEVKREIQELKAEALAKPV-----
--EEKSN---RTEYATSFFFQLKTVLRRTNVALWRNADYQWTRLFAHLAI
GLIVTL-TFLQ--LDNSVQSLQYRVFAIFFATVLPALILAQIEPQYIMSR
MTF-NREASSKMYSSTVFALTQLLSEMPYSLGCAVSFFLLLYYGVGFPYA
S-------SRAGYFFLMILVTEVYAVTLGQAVAALSPTILIAALFNPFLL
VL-FSIFCGVTAPPPTLPYFWRKWMWPLDPFTRLISGLVSTVLQDQEVVC
KDGEYQVFPA--PSG-QTCQQWAGAFAE-----------AIGGYINNPDS
----TGDCQFCQYRT--GQAF--FTPLEIS-FSTRWRDFGIFICYVVFNI
LVLLIAARFLKWQRR-----------------------------------

>Q4WUS1

-------------------------MDEKPAVSESSNGSDVDSLSTASAY
EQHRERLRDANPQGVTSHRSGVNVKEAEEEFSELNRQFSTISHQAHCLSK
QISRASKPTGK--TEDVERSDSPADSDEPWDLETALRGNR---DAETAAG
IRSKRIGVIWDNLTVRGMGGVKTYIKTFPDAIIDFFNVPETIMHMLGYGK
--KGKE-----FEILRNFRGVLQPGEMVLVLGRPGSGCTTFLKTITNQRF
GYTSID---GDV------LYGIFDAD---TFAKRFRGEAV--YNQEDDVH
QPTLTVKQTLGFALDTKTPGKRPL-----GVSKAEFREKVINMLLKMFNI
EHTANTVIGNQFIRGVSGGERRRVSIAEMMITSATVLAWDNSTRGLDAST
ALDFAKSLRIMTNIYKTTTFVSLYQASENIYKQFDKVLVIDSGRQVFFGP
ASEARSYFES-LGFKE-RPRQTTPDYLTGCTDPFEREF---KEGRSED--
DVPSTPDSLVEAFNRSSYSERLAQEMDAYRKKL-----------------
--EQEKHVYEDFEIANQEAKRKFTPKSSV--YSIPFHLQIWALMQRQFLI
KWQDRFAQTVSWITSTGVAIILGTVWLRL----PKTSAGAFTRGGLLFIS
LLFNGFQAFS-ELVSTMMGR-SIVNKHRQFTFYRPSALWIA-QILVDTTF
AIARILVFSIIVYFMCGLVLDAGAFFTFILIIVLGYLCMTCFFRVIGCMS
PDFDYAMKFASVVITLFVLTSGYLIQWSSEQ---EWLRWLYYINPFGLGF
AALMVNEFKDLTMTCTADSLVP----SG--PGYDDM--ASRVCTLA-GGE
PGSVIIPGAS-YLAKTFSYFPGDLWRNFGIMVALTVGFLTLNLYHGETLQ
FGAGGRTVTFYQKENKERRALNGALMEKRTNRESKD--QSAANLK-----
---I
TSKSVFTWEDVCY---DVPVPS-GTRRLLQSVYGYVQPGKLTALMGASGA
GKTTLLDVLASRKNIGVI-----SGNILVDGAPPPG--SFLRTVSYAEQL
DIH-EPMQTVREALRFSADLRQPYETPQSEKYEYVEGIIQLLELEDLADA
IIGT--PETGLSVEERKRVTIGVELAAKPELLLFLDEPTSGLDSQSAFNI
IRFLRKL-AAAGQAILCTIHQPNSALFENFDRLLLLQRGGECVYFGDIGE
DSHVLLDYFRRNGA-DCPPDANPAEWMLDAIGAGQT--------------
-------RRIGD-RDWGEIWRTSSEFEQVKREIIQIKAQRAEEVR-----
--QSGGSQIIVREYATPLWHQIKVVCKRTNIVFWRSRNYGFTRLFNHVVI
ALVTGL-AFLN--LDDSRASLQYRIFVIFNVTVLPAIILQQVEPRFEFSR
LVF-FRESACKSYSQFAFALSMVIAELPYSILCAVCFFLPLYYIPGFQAA
P-------SRAGYQFLMVLITELFSVTLGQMISALTPNSFIASQINPPIV
II-FSLFCGVAIPRPQMPGFWRAWLYQLDPFTRLISGMVTTELHGRTVSC

SPSEFNRFQA--PEN-QTCGEYMLPFFE-----------RGGLGYLADNT
----TQACEYCAYKI--GDEF--YSAFSMS-FNTRWRDLGIFLAFIGSNL
IILFLAVSFMSPRCRLRYKLIYDIF-------------------------

>B8M035

-------------------------------MSSPAIHTEFWTKEDESVL
TEVTYEDGREQVRTSNPTGLSRIQSGVDVERAERDFAELNQQFSNISQQA
RRLSKQASQRSKCASVHDLEKTGISNASSDELWDLETSLHGSKAAENDAG
IRPKHIGVIWDGLTVRGFGGVKTFVQTFPDVVIGFFNVYATIKSLLGL--
-----QKQGVEVDILHNFRGVLKPGEMVLVLGRPGSGCTTFLKVITNQRY
GYTSFD---GAV------SYGPFDSS---TFAKRFRGEAV--YNQEDDVH
HPTLTVGQTLAFALDTKTPGKRPA-----GVSKKEFKEKVIQMLLKMFNI
EHTVNTVVGNAFVRGVSGGERKRVSIAEMMITSGTVLAWDNTTRGLDAST
ALDFSKSLRIMTNVYKTTTFVSLYQASENIYEQFDKVMVIDEGRQVFFGP
TTEARAYFEG-LGFML-KPRQTTPDYLTSCTDPFEREY---QDGRNSD--
NVPSTPDALVKAFDGSKYRALLDQEIAAYRTQI-----------------
--QEEKHVYEEFELAHQEAKRKHTPKSSV--YSIPFYLQIWALMKRQFLV
KWQDKFSLTVSWSTSIITAIVLGTVWYKL----PTNSSGAFTRGGLLFIS
LLFNAFQAFA-ELGSTMLGR-PIVNKHKAYTFHRPSALWIA-QILVDTAF
AAVQILVFSIIVYFMCGLVLDAGAFFTFVLIIITGYLSMTLFFRTIGCLC
PDFDYAMKFAAVIITLYVLTAGYLIQYQSEQ---VWLRWIFYINALGLGF
AALMVNEFKRITLTCSTSSLVP---------SYGDIA--HQTCTLQ-GSS
PGSNIISGSA-YLSAGFSYETGDLWRNFGIIVVLIAFFLFTNAYLGESVN
WGAGGRTITFYQKENAERKKLNEELIAKKQRRQNKEAVDSSSNLN-----
---I
TSKAVLTWEGINY---DVPVPS-GTRQLLNSVYGYVQPGKLTALMGPSGA
GKTTLLDVLAARKSIGVI-----TGDILVDGHKPGA--SFQRGTSYAEQQ
DVH-EPTQTVREALRFSAELRQPYHVPLEEKHAYVEEIISLLELEILADA
VIGF--PEIGLSVEERKRVTIGVELAAKPELLLFLDEPTSGLDSQSAFNI
VRFLRKL-AAAGQAILCTIHQPNSALFSSFDRLLLLQRGGNCVYFGDIGE
DSRVLIDYFRRNGA-QCPPNANPAEWMLDAIGAGQT--------------
--------PRIGDRDWDDIWRESPELAQIKEDITKMKNERAAQNR-----
---SSESSSQEVEYATPTWYQIKTVVRRTNLSFWRSPNYGFTRLFVHAVI
ALLTGL-MFLQ--LDDSRSSLQYRVFVLFQITVIPAIIIQQVEPKYELSR
-LISYRESASKTYKSLAFAIAMVVAEVPYSLLCTVAFFLPIYYIPGFQSA
S-------DRAGYQFLMVLITEFFAVTLGQMVAAITPSSYISAQLNPPLI
IT-FALFCGVAIPKPQIPKFWRAWLYQLDPFTRLIGGMVVTELHDREVVC
KNSELNTFSA--PDG-QTCGEYMAPYFA----------AGAPGYLVNNA-
----TNTCQYCAYKV--GDQF--YEAFDLS-YDNRWRDLGIFACFIVSNI
VILSLGVSKLSYMFNYKPTNSTARHDT-----------------------

>B8M9S3

-------------------------------MSSPAIHTESWTKEDESVR
TEVTYEDGREQVRTSNPTGLSRIQSGVDVERAERDFAELNRQFSNISQQT
RRLSKQASRPSKRASVHDLEKTGSSNASSDDPWDLETSLHGSKAAEVEAG
IKPKHIGVVWDGLTVRGFGGVKTFVQTFPDAVIGFFNVYATIKTLLGF--
-----QKQGAEVDILHNFRGVLKPGEMVLVLGRPGSGCTTFLKVITNQRY
GYTSFD---GEV------SYGPFDSN---TFAKRFRGEAV--YNQEDDVH
HPTLTVGQTLSFALDTKTPGKRPA-----GVSKKEFKEKVIQLLLKMFNI
EHTVNTVVGNAFVRGVSGGERKRVSIAEMMITSGTVLAWDNTTRGLDAST
ALDFAKSLRIMTNIYKTTTFVSLYQASENIYEQFDKVMVIDEGRQVFFGP
TTEARAYFEG-LGFMP-KPRQTTPDYLTGCTDPFEREY---QDGRNSD--
NVPSTPDALVKAFDESKYRTLLDQEIAAYRTQI-----------------
--QEEKHVYEEFELAHQEAKRKHTAKSSV--YSIPFYLQIWALMKRQFLV
KWQDKFTLTVSWATSIITAIVLGTVWYKL----PTNSSGAFTRGGLLFIS
LLFNAFQAFA-ELGSTMLGR-PIVNKHKAYTFHRPSALWIA-QILVDTAF
AAVQILVFSIIVYFMCGLVLDAGAFFTFVLIIITGYLSMTLFFRTIGCLC
PDFDYAMKFAAVIITLYVLTAGYLIQYQSEQ---VWLRWIFYINALGLGF
AALMVNEFKRITLTCSTSSLVP---------SYGDIA--HQTCTLQ-GSS
PGSDIIPGSA-YLSAGFSYETGDLWRNFGIIVALIAFFLFTNAYLGESVN
WGAGGRTITFYQKENAERKKLNEELMAKKQRRQNKEAVDSSSNLN-----
---I
TSKAVLTWEDVNY---DVPVPS-GTRRLLNSVYGYVQPGKLTALMGASGA
GKTTLLDVLAARKSIGVI-----TGDILVDGHRPGA--SFQRGTSYAEQL
DVH-EPTQTVREALRFSAELRQPYHVPLEEKHAYVEEIISLLELETLADA
VIGF--PEIGLSVEERKRVTIGVELAAKPELLLFLDEPTSGLDSQSAFNI
VRFLRKL-AAAGQAILCTIHQPNSALFSSFDRLLLLQKGGNCVYFGDIGE
DSRVLIDYFRRNGA-ECPPNANPAEWMLDAIGAGQT--------------
--------PRIGDRDWGDIWRESPELAQIKEDITKMKNERSAQNS-----
---SSGSSSQEVEYATPTWYQIKTVVRRTNLSFWRSPNYGFTRLFVHAVI
ALLTGL-MFLQ--LDDSRSSLQYRVFVLFQITVIPAIIIQQVEPKYEFSR
-LISYRESASKTYKSLAFAIAMVVAEVPYSLLCTVAFFLPIYYIPGFQSA
S-------DRAGYQFLMVLITEFFAVTLGQMVAAITPSSYISAQLNPPLI
IT-FALFCGVAIPKPQIPKFWRAWLYQLDPFTRLIGGMLVTELHDREVVC
KNAELNTFSA--PDG-QTCGEYMAPYFA----------AGAPGYLVNNA-
----TSACQYCAYKV--GDQF--YEAFDLS-YNNRWRDLGIFACFIVSNI
VILFLGARYLNFNKR-----------------------------------

>Q1DSU0

--------------------------MPGSGSGDPPPQTREPTDSSDAET
LFTSSDYEARRENLRNENPQGLTRPTSGVDVQRAEEEFAQLSKQLTGISE
RAKRLSRQQSNRLDEKTPEDIETSTEGEPFDLETTLRGSR---TAEADAG
IRPKRIGVIWDGLTVRGIGGVRNIVRTFPNAVVDFFNVPQTIMHILGLGR
--KGKE-----FEILKNFKGVAKPGEMVLVLGKPSAGCTTFLKVIANQRF
GYTGVD---GEV------RYGPFDAS---AFAKRFRGEAV--YNQEDDVH
HPTLTVGQTLGFALDTKTPGKRPA-----GMSKAEFKEKIINLLLKMFNI
EHTINTVVGNQFVRGVSGGERKRVSIAEMMVTSATVLAWDNTTRGLDAST
ALDYAKSLRILTNIYQTTTFVSLYQASENIYDQFNKVMVIDSGRQVYFGP
TKEARAYFED-LGFKE-KPRQTTPDYLTGCTDPFEREY---KEGRNAE--
NTPSTPDALVQAFEKSRFNEALEQEMDTYRAQL-----------------
--DQEKHVYDDFEMAHLEAKRKFTSKSSV--YSIPFYLQVWALMQRQFLI
KWQDKFSLAVSWITSIGVAIVLGTVWLKL----PTTSAGAFTRGGVLFIS
LLFNALQAFS-ELASTMLGR-PIVNKHRAYTFHRPSALWIA-QIAVDLAF
ASVQIFVFSVIVYFMCGLVLDAGAFFTFVLIIITGYLSMTLFFRTVGCVC
PDFDYALKGVSIIITLFVVTSGYLIQWQDQQ---VWLRWFFYINAVGLGF
SGLMMNEFGRLNMTCTPESLIP----AG--PGYTNL--SHQVCTLP-GGD
PGSSIIPGSN-YIKLQFRYDPADLWRNWGIMVVLIVVFLCANAYLGEALT
YGAGGKTVTFFAKETHELKKLNSELQEKKRNRQEKKSEESESNLK-----
---I
ESKSVLSWEDLCY---DVPVPG-GTRRLLNNVFGYVEPGKLTALMGASGA
GKTTLLDVLAARKNIGVI-----TGDILVDGRTPGS--SFQRGTSYAEQL
DVH-EPTQTVREALRFSATLRQPYEVPEEEKFAYVEEIISLLELENLADA
IIGD--PETGLSVEERKRVTIGVELAAKPQLLLFLDEPTSGLDSQSAFNI
VRFLRKL-AAAGQAILCTIHQPNSALFENFDRLLLLQRGGECVYFGDIGT
DARILRDYFHRNGA-DCPSNANPAEWMLDAIGAGQT--------------
-------PRIGS-RDWGDVWETSPEFEQVKQRIVEIKDERVKATE-----
--GASASADAEKEYATPLWHQIKVVCRRTNLAFWRSPNYGFTRLFSHVAL
ALITGL-CYLQ--LNDSRSSLQYRIFVLFQITVIPALILAQVEPKYDMSR
LIF-YRESAAKAYKQFPFALSMVLAEVPYSILCAVCFFLPLYYIPGLQSA
S-------SRAGYQFFMILITEFFAVTLGQTISALTPSTFIAMLLNPPVI
II-FFLFCGVSIPRPQIPKFWRVWLYELDPFTRLMSGMIVTELHDRPVTC
KPEELNRFVP--PPG-QDCFSYMKEFFA-----------NGGPGYLVKNA
----TDICEYCAYKV--GDQF--YKPFGME-FSNRWRDLGIFLCFIASNL
ILLFVGSRYLNFNRR-----------------------------------

>Q4P2M1

-------------------------MSDPTSFSGISTPAGPAAMYTPDNR
SSANGDHSVSIDDNSGAKDSHVDVDRAKAEFHQLQTSLSRQSETTRQNSQ

EAQDIE----------AGQKGGDNDDDDDFDLVQYLRSVQ---SENAQAG
IKSKHIGVSWSDLEVIGNDSMSLNIRTFPDAITGL--FLGPLFSIMSRLN
--KNRG-----RKLLQNFNGVAKPGEMVLVVGRPGSGCSTFLKTIANQRG
GYIGVN---GDV------KYGGIPSQ---EFARKYQGEAV--YNEEDDVH
FPTLTVKQTLEFALSLKSPGKRLP-----HQTVKSLNEEVLNTFLKMLGI
PHTANTLVGSAVVRGVSGGERKRVSIAECMASRAAVVSWDNSTRGLDAST
ALDYAKCMRVFTDILGLTTFITLYQPGEGIWEQFDKVMVIDEGRCVYYGP
RIKARQYFLD-LGFKD-YPRQTSADFCSGCTDPNLDRF---AEGQDEN--
TVPSTSERLEEVYHNSSIYQDMLRQKQEYDAQI----------------
--AADRSAEEEFRQAVLEDKHKGVRPKSI--YTVSFARQVQALTVRQMQM
ILGNQFDIFVSFATTITIALIVGGIFLNL----PETAAGGFTRGGVLFIG
LLFNALTAFS-ELPTQMGGR-PVLFKQMNYAFYRPAALSLA-QLFSDIPL
SLGRVILFSIILYFMAGLERSAGAFFTFFLFVYFGYLAMSALFRLFGTVC
KSYDVAARLAAVIISALVVFAGYVIPRDAMY---RWLFWISYLNPLYFAF
SGLMMNEFKNLSLACVGTYIVPRNPPGS--TQYPDNVGQNQVCTLP-GAR
AGQQFVAGND-YLRASFGYDSGDLWLYFGVTVIFFVGLVGITMVAIEIFQ
HGKHSSALTIVKKPNKEEQKLNQRLKERASMKEKDSSKQLD---------

VESKPFTWEKLCY---EVPVKG-GKRQLLDNVYGYCRPGTLTALMGASGA
GKTTLLDVLADRKSIGVI-----SGERLIDGKKIGI--EFQRGCGYAEQQ
DIH-EGTATVREALRFSAYLRQPPSVPKEDKDAYVEDIIELLEMQDIADA
MIGI--PEFGLGIGDRKRVTIGVELAARPDLLLFLDEPTSGLDGQTAYNV
VRFLKKL-AASGQAILCTIHQPNALLFEQFDRLLLLERGGKTVYFGDVGP
NAKHIVKYFADRGA-ECPGNVNMAEYMLDAIGAGSM-------------
-------KRVGD-KPWSELYKESDLFQHNLAEIEKIKQESSSSTS-----
--QGSEQS-HKTEYATPFVYQVKTVLHRALLSTWRQPDYQFTRLFQHAAI
ALISGL-CFLN--LDNSVASLQYRIFGIFMATVLPAIILAQIEPFFIMSR
SVF-IREDSSKMYSGVVFAIVQLIQEVPFGIVSTVVYFLLFYYPAGFQTG
S-------DRAGYFFAMLLVTEMFAVTLGQAIAAISPSIYIASLFNPFMI
VI-MSLLCGVTIPYPNMPSFFRSWLYWVNPLTYLVSGLVTNEMHNLTVEC
TATELARFNP--PSG-QTCRAWAGAFVD-----------AFGGYLQNPDA
----TSDCQYCQYRN--GDQF--YAGLNID-FSERGRNIGLVIVYVAFNA
IVTILASRFIKYANR-----------------------------------

>P41820

-----------------MNQNSDTTHGQALGSTLNHTTEVTRISNSSDHF
EDSSSNVDESLDSSNPSSNEKASHTNEEYRSKGNQSYVPSSSNEPSPESS
SNSDSSSSDDSSV---DRLAGDPFELGENFNLKHYLRAYK-DSLQRDDI-
-ITRSSGVCMRDHSVYGVGSGYEFLKTFPDIFLQPYRAIT-----EK---
--QVVE-----KAILSHCHALANAGELVMVLGQPGSGCSTFLRSVTSDTV
HYK-RVE--GTT------HYDGIDKA---DMKKFFPGDLL--YSGENDVH
FPSLTTAETLDFAAKCRTPNNRPC-----NLTRQEYVSRERHLIATAFGL
THTFNTKVGNDFVRGVSGGERKRVTISEGFATRPTIACWDNSTRGLDSST

AFEFVNVLRTCANELKMTSFVTAYQASEKIYKLFDRICVLYAGRQIYYGP
ADKAKQYFLD-MGFDC-HPRETTPDFLTAISDPKARFP---RKG-FEN--
RVPRTPDEFEQMWRNSSVYADLMAEMESYDKRWTETTPASSEAPEKDNFG
SDISATTKHELYRQSAVAEKSKRVKDTSP--YTVTFSQQLWYCLARSWER
YINDPAYIGSMAFAFLFQSLIIGSIFYDM----KLNTVDVFSRGGVLFFS
ILFCALQSLS-EIANMFSQR-PIIAKHRASALYHPAADVIS-SLIVDLPF
RFINISVFSIVLYFLTNLKRTAGGFWTYFLFLFIGATCMSAFFRSLAGIM
PNVESASALGGIGVLAIAIYTGYAIPNIDVG---WWFRWIAYLDPLQFGF
ESLMINEFKARQFEC--SQLIP----YG--SGYDNYPVANKICPVT-SAE
PGTDYVDGST-YLYISFNYKTRQLWRNLAIIIGYYAFLVFVNIVASETLN
FNDLKGEYLVFRRGHAPDAVKAAVNEGGKPLDLETGQDTQGGDVVKESPD
NEEELN-------------------------------------KEYEGIE
KGHDIFSWRNLNY---DIQIKG-EHRRLLNGVQGFVVPGKLTALMGESGA
GKTTLLNVLAQRVDTGVV----T-GDMLVNGRGLDS--TFQRRTGYVQQQ
DVH-IGESTVREALRFSAALRQPASVPLSEKYEYVESVIKLLEMESYAEA
IIGT--PGSGLNVEQRKRATIGVELAAKPALLLFLDEPTSGLDSQSAWSI
VCFLRKL-ADAGQAILCTIHQPSAVLFDQFDRLLLLQKGGKTVYFGDIGE
HSKTLLNYFESHGAVHCPDDGNPAEYILDVIGAGAT--------------
-------AT-TN-RDWHEVWNNSEERKAISAELDKINASF-------SNS
EDKKTLSKEDRSTYAMPLWFQVKMVMTRNFQSYWREPSILMSKLALDIFA
GLFIGF-TFYN--QGLGVQNIQNKLFAVFMATVLAVPLINGLQPKFIELR
NVFEVREKPSNIYSWVAFVFSAIIVEIPFNLVFGTLFFLCWFYPIKFYKH
I---HHPGDKTGYAWLLYMFFQMYFSTFGQAVASACPNAQTASVVNSLLF
TF-VITFNGVLQPNSNLVGFW-HWMHSLTPFTYLIEGLLSDLVHGLPVEC
KSHEMLTIN---PPSGQTCGEYMSAFLT--------NNT-AAGNLLNPNA
----TTSCSYCPYQT--ADQFL--ERFSMR-YTHRWRNLGIFVGYVFFNI
FAVLLLFYVFRVMKLRSTWLGKKITGTG----------------------

>A1C8C8

----------------------------MNEDHEAGNSSREAEVHQLARQ
FTDQSNYSAAGQNPFAVEAGSALDPNGEHFNARAWCKAML-QMHTGDKQA
HPLRTLGVAFSNLNVHGFGSDTDYQKSVGNVWLEVLSLVSKAF--GQKQR
----------KIEILQNMEGLVEAGEMLVVLGPPGSGCSTFLKTIAGETY
GFHVDKN--SNI------NFQGIA-K---QMAHEFRGEAI--YTAEVDVH
FPKLTVGDTLYFAARARAPRHIPG-----GVNATQYASHMRDVIMAMFGI
SHTKNTIVGNDFIRGVSGGERKRVSIAEACLSNAPLQCWDNSTRGLDSAN
AIEFCKTLRMQADINGTTACVSLYQAPQAAYDYFDKALVLYEGREIYFGR
TSMAKQYFLD-MGFVC-PDRQTDADFLTSMTSHLERVV---QPG-YEG--
RVPRTPDEFAARWRASPQRAQLLQDIKCYNAKF-----------------
--ALDGEYLDKLKQSRRAQQAKAQRVSSP--YTLSYVQQVELCLWRGYQR
LKADPSVTISSVFGNTIISLVIASIFYNL----KADTSTFFQRGALLFFA
VLMNALGCGL-EMLTLYAQR-GIIEKHSRYALYHPSAEAFS-SMIMDLPY

KIINAITSNIVLYFMTNLRREPGAFFFFVFTSFVLTLTMSMFFRSMASLS
RSLVQALPFSAVLLLGLSMYTGFTIPTGYML---GWARWIAYINPISYGF
ESLLINEFHNRDFPC--MNYVP----SG--PGYTDLGLNNRVCSTV-GSV
PGQAFVNGDA-YIESAYIYTASHKWRNIGVIFAYMFLLAAVYLVATDFIT
EKKSKGEILVFPRGH---EALKKGKSDEDLEEGSGRSVTVEKTGSDGLTM
--IE
RQTAIFQWKDVCF---DIKIGK-ENRRILDHVDGWVKPGTLTALMGVSGA
GKTTLLDVLATRTSVGII-----SGEILVDGQPRDD--SFQRKTGYAQQQ
DLH-LSTATVREALEFSALLRQSAHVPRQEKIDYVTEVIKLLDMTEYADA
VIGV--PGEGLNVEQRKRLTIGVELAARPQLLLFLDEPTSGLDSQTSWAI
LDLLDKL-KKNGQAILCTIHQPSAMLFQRFDRLLFLQAGGRTVYFGEVGE
NSQILIDYFVRNGGPPCPPAANPAEWMLDVIGAAPG--------------
---------SHTNINWFETWRKSPEYARVQEHLAELKRERPEQTNLFRTT
SSQKREDKASYREFAAPFCAQLCEVQVRVFQQIWRSPTYIYSKTALCVLS
ALFVGF-SLFH--TPNTIQGLQNQMFGIFMLLTVFGQLIQQIMPHFVAQR
ALYEVRERPAKTYSWKAFIISNIVVELPWNSLMSVLMFLCWYYPIGLYRN
AEPTDAVSLRGTQMWLMVWTFLLFSSTFAHFMIAAFDAAENAGNLGNLLF
LL-CLIFCGVLATPGQLPGFW-IFMYRVSPFTYLVSGMLSVGISNTNATC
ADNEYLRFDP--VNG--TCGKYMDSYIS-----------NMGGYLEDEMA
----TSDCSFCPIKE--TNVF--LSSVSSS-YSEIWRNFGLMWVFIVFNI
FAACLLYWWVRVPRVKKPVAKTE---------------------------

>B6H475

----------MADCDLANTRVRTGSSSAEHTSADDQANELHDRVAKVAER
LSICSGSGERQSEVLKPTPGSKLDPSSPAFDARVWVKEFI-RLTESDPDS
APSRSLGVAFKNLGVFAYTTAAEFQKTTGNVIIGLTTYLARCLRGNRHGR
----------RVDILRNFEGVAEKGEMLLVLGPPGSGCSTLLKTLSGETA
GLNITPE--SYI------NFRGLDVA---NIRSSLRGDVL--YNAELDVH
LAHLTVGETLTFASRARSVRHVPG-----GFDRHQLNTVRRDVMMAIFGL
NHTINTHVGDDFVRGVSGGERKRVSIAEASLTGAKFQCWDNSTRGLDSAN
AINFCSNLRLQADLLDVTSVVTLYQAPQSAYDLFDRVTVIYEGRQIFFGR
ITEAKGYFES-LGFDCESSRQTVPDFLTSMTSPEERRA---RAG-FDD--
SVPRSADEFAERWQSSEQRRNLLSELASYEQRH-----------------
--PPE-ERTAEYYHSRRAEQAKQQRSKSA--YTISYAQQISLTLWRAYRR
LLADPGFTIASLLFNVAMALILGSMFYDL----KPDTSSFYYRGGLIFFS
LLFNAFASQL-EVLTIYAER-PVVEKQNRYAFYHQSAQAIA-SYLTDLPY
KIVNMFVFNILIYFMASLKREAGAFFFFCLTTFLTTLVQSAIFRTLACIT
RTSDQAMIPSAVLSLGLMIYTGFTTPPEYMP---GWSRWMAYINPLAYGF
EALMANEFHHREFPC--VDMVP----SG--QGYTNLPSESHICSVV-GSE
AGSAFVNGDN-YINRSFDYWNVHKWRNIGILCAFLAFFFPAYVLAAELAK
PPRTRGEILVFRRKKGSPKPEKALSLDAENQMQGRPVVAEKLNPPSRSDD
--IV

```
VGKDVFHWEDLCY---DIEIKG-GNRRLLDHVDGWVKPGVSTILMGVSGA
GKTTLLDVLATRVTTGVV-----TGSTMVNGSPTDP--SFQHKVGYVQQQ
DLH-LSTMTVREALRFSAVLRQSAEITRSEKLNYVEHVIDTLEMREFSDA
VIGV--PGEGLNVEQRKRLTIGVELAARPQLLIFFDELTSGLDSQTSWSI
SELIKKL-TNSGQAVLCTIHQPSAILFDQFDRLLLIAPGGKTAYFGDLGS
GASTMINYFEKNNAPACPAGANPAEWMLQVIEPPTD--------------
---------GSKSPDWHQVWLDSPEYRTVKAELGRLRALPATES---AEN
EDTTDGDTSQHQEFVASFWTQFGQVLQRTGKHLWRSPTYIWSKTILVVLS
SLYLGF-SF-E--ANNSIQGLQNQLWAIFMLLVLFININEQIMPIFLPQR
ALYEARERPSKIYRWTTYLLSNILIELAWHTLMAVIMYFCWYYPVGFVRN
T-SSDDQAIRGFLVFLFLWVYLLFTSTFAHSAIFWIDLPETAGVLTSLFW
ML-CILFCGVGVPIIDLPKFW-SFMYRVSPATYLVGGIMSNAVANSNVTC
ADREILHMVP--IGN-LTCEEFLGAYRE-----------AAGGLVLNPAA
----RSMCEYCPLAT--TNEF--LDRFQIS-YDTRWRDFGLIWVYILVNI
VAGLGLYWAFKVPKKGGSKRA-----------------------------
--------------------------------------------------
--------------------------------------------------
--------------------------------------------------
-----------------------------
>A2QT53
--------------------------------------------------
--------------------------------------------------
--------------------------------------------------
--------------------------------------------------
--------------------------------------------------
--------------------------------------------------
--------------------------------------------------
--------------------------------------------------
--------------------------------------------------
-----MVTGPPVEGAGNEEKEHYDSTTTLSEHQQEELRKIASRQSAVSTT
GGKHSEQGFGSHI---DP-------TSPHFDLSQWMRYQV-QQFEEDSS-
--ARKTGVVFKDVTVQGRGAEIQIQHTVLSSVYAPILGGAFR---RG---
--SNKA-----KTILHDVHGHIEQGEMLLVLGRPGAGCSTMLKTISADTN
GLDLSSS--SVI------SYNGILQH---LMQKNYKGELL--YNQEVEKH
FPHLTVGETLNFAAAARMPRILPN-----GMSKQEYIKHMRDVVMAVFGL
SHTVNTKVGSDFVRGVSGGERKRVSIAEMALAGSPLCCWDNATRGLDSAS
SLDFVKALRTSSRIFGTTHVSTLYQPSQAVYHCFDKVMVLYQGHEIFFGP
TTEAKQYFED-MGWYC-PARQTTADFLTSITNPSERRP---REG-FEA--
RVPRTPEEFEMYWRNSTTYKRLMDDISSYEVKF-----------------
--GADCGATEAFKQSHARRQARYARSSSP--YLIDIPTQIEICASRFYQR
VWNDIPSTLTLMIGQVVFSIIIGSLFYGS----AFGTEDFTLKMSALFFA
ILLNSLLTVT-EIQNLYAQR-PIVEKQASYAFYHPFTEALA-GVCADIPI
KVGCSLMFNIVFYFMCGFRYEAGPFFVFYLFVTMALLCMSQIFRSLAAAT
KAIPQALAAAGVILLATVIYTGYLLPQPSMH---PWFKWISYINPLMYAF
EALAVNEFHGRDFPC--SDLVP----LY--PGLKNGSGTYFICA-A-GAV
AGELYVSGDD-FLSVSYGYEYSHLWRNFGILCAFVIAFLALYLVLTEINS
QSSSTAESLVFRHGR-----IP-----------VAFEKSANDPKAANVSA
TQGQET-------------------------------------GDETVMP
PHHDTFMWREVCY---DIEIKK-EERRLLDKDSGWVEPGTLTALMGVSGA
GKTTLLNVLAQRTSTGVI----T-GDMLVNGSPLTA--SFQRSTGYVQQQ
DLH-LHTATVRESLRFSALLRQPKSVPVQEKYDFVEKVISMLGMEEFAEA
VVGF--PGEGLNVEQRKLLTIGVELAAKPALLLFLDEPTSGLDSQSSWTI
IALLRRL-ASSGQAILCTIHQPSAMLFQQFDRLLFLAKGGRTVYFGDIGP
NSRTMLDYFEQK-ARRCDDSENPAEYILEIAGAGVN--------------
```

-------GK-AE-QDWPTVWKESPECTEMMKILEKRCAAVRY-----TDK
TDKQAEAEGAEDAFAMPFRVQFAAVLRRIFQQYWRSPEYIYGKLALGILS
ALFVGF-SFYL--PGTSQQGLQSSIFSVFMITAIFTALVQQIMPQFIFQR
DLYEVREQPSKTYHWAAFLGANLIAEIPYQMFVAILVYASFVYPVYGIAD
S-------QRQGIMLLLIIQFFIYGSTFAHAVVAVLPDAETAGLIATMLF
NM-TLVFNGILVPRVALPGFW-DFMYRVSPMTYLVNAIIASGVSGRAVNC
SEKELSVFSV--APGYDSCGQYMEAYLE--------AAGTAAGKLLNPES
----TDQCKYCTLQT--ADQFL--ASRDIY-YDQRWRNFGLLWAYIAFNA
AFAFAIYYLSRVRSWKRKA-------------------------------

>B0DP88

--MSDPLP
L---
------------TDIAEETPSGSVSRAASYPTLYNVDTPLSQHAEEDHHA
SNVHANPHGHPDHSPPPPVSPKMGYTRARSGSSVSHVNIEFFDPEGVQAL
RRTMTEEAQNRGR---PSAASSSTDHTLTDGPFDFEKTIRHHVRRREEAQ
ITQRSIGVGFQNLRVVGLGASASYQPTLGSLLNPLGVLETVQN--IR---
--HPAV-----RDILSGFEGVIRPGEMILVLGRPGSGCSTLLKTLANQRQ
EFH-AVE--GDV------HYDSLSPQ---DIHDHFRGDVQ--YCPEDDVH
FPTLTVEQTLKFAVTTRTPRARV------DVSREQFQDETVKVLTTIFGL
RHTLNTPVGDAAIRGVSGGEKKRVSIAEAMATRSCVGAWDNSTRGLDSST
ALEFVKALRIATDVFHATTIVSIYQAGESLYKHFDKVCVIYEGRMAYFGT
ADKARQHFID-MGYEP-ANRQTTADFLVAVTDPNGRIP---RAG-VIS--
Q-PRTAAEFAEYFLKSEAGKENRADLDSYLEEF-----------------
--VGKPHVASAYMTSARAEFAKGSGKKNP--YMLTIPQQVRAVMKRRVQI
IRGNLLATGLQVFSFIFQSLIMGSVFLKM----PQNTANFFSRGGILFFA
LLFSALTSMA-EIPALYSQR-PIVLRHERAALYHPFIEALA-LTLVDVPL
TFLTTIVFSIILYFMTGLQRTASQFFVFILFLFTMSITMKAWFRTIAAAF
KSEAAAQSVAGIAILALSIYTGFTIPKPSMI---GALRWITYINPLRYGF
EAMITNEFRTLEGEC--SSLVP----RG--PGYENITLANQVCTTV-GSV
PGQPFVDGNR-FAAISYGFFWSKTWMNFGIVIAFGVGFLTFLLLFTEFNT
ASAVESSVMLFKRGTGPNNGSNV--------NDEEAINEKDSRGLVISDD
DEKLQK-------------------------------------EDTPSAP
AMNDVFTWQRVRY---TVPIAGQDDRLLLSDVSGYVAPGKLTALMGESGA
GKTTLLNVLAQRVSTGVV----T-GDRFVNGQALPA--DFQSQSGYCQQM
DTH-VPTATVREALLFSAKLRQPKSVPLAEKEAYVEKCLKMCGLEKYANA
SVG------SLGIEHRKRTTIAVELAAKPKLLLFLDEPTSGLDSQSAWAI
MSFLRSL-ADNGQAILCTIHQPSAELFQVFDRLLLLQKGGRTAYFGDLGR
NATTLIDYFEKNGARPCLDDENPAEYMLDVIGAGAT--------------
-------AT-SK-QDWYQLWQSSQESKDNQQEIEAIH----------AEG
RNRPAIAASIRTEFATPWAYQVVELLKRDAEAHWRNPTYLMAKLILNIVG
GLFIGF-TFFK--AKHSIQGTQNKLFAIFMATILSVPLSNQLQVPFIDMR
TVYEIRERPSRMYSWTALVTSQVLIELPWNIVGATLLFLTWFWTVGFESS
R--------GGYTYLMLGIAFPLYYTTIAQAVAAMAPSAEIAALLFSFLF
SF-VITFNGVLQ-PFAQLGWW-RWMYRLSPYTYLIEGLLGQAIGGQQVSC

SPVEFVSIT---PPSGMTCGEYMNPFIS--------VAG---GYLTNADA
----TSACQFCSIRT--TDQFL-NAAFNIF-YNHHWRNFGFMMAFFVFNF
SCIYLLTYIFRIREGSILPSFKRRAAKK----------------------

>Q9Y840

---------------------------MWGYSVDERKLQREDTNGPPT--

------------NQWHNAQRTGGTHPTEEVEGEGEGTWGENDVGGFTTRQ
AMEDYEALRKDLTQLSKTRSRDTQHSLKRTTTGQTAKSGRKSLTSRQATH
TSEAAEQDVEAGPQEEVEESKKDDDDEDDFELDRFMREGH---FEKRSDG
TSDKRVGVVYKDLTVKGIGSTTSFVRTLPDAIIGTFGPDLFKIICRFVPA
LAKRTGET---RTLLNGFTGCVRDGEMMLVLGRPGSGCSTFLKAISNNRE
TYAEVT---GDV------SYGGIPAD---KQKKMYRGEVV--YNQEDDVH
FATLNVWQTFIFALMNKTKKKET-----------GNIPVIAEALMKMFGI
PHTKYTLVGDDFVRGVSGGERKRVSIAETLASKSTVVCWDNSTRGLDAST
ALDYARSLRVMTDVSNRTTLVTLYQAGEGIYEVMDKVLVIDEGREIYSGP
AKEARQYFID-LGYEA-PERQTTADFLTAVTDPVERKF---RKG-YEH--
KAPKGPEALEKAFRESPNYQKVLEDITDYENYL-----------------
-KETDYNDAREFEDAVQDGKSKRVSNKSS--YTVSFQRQVLACVKREAWL
LWGDKTTLWTKLFIIISNGLIVGSLFYGE----SFDTSGAFTRGGALFFS
ILFLGWLQLT-ELMKAVSGR-AVVKRHEDYAFYRPSAVTIA-RVVMDLPV
ILVQVLIFGIIMFFMTNMTISASQFFIYMLFVYITTILLTALYRMFASLS
PEIDTAVRFSGIALNLLVIYTGYVIPRPQLLTKYIWFGWIYWINPLSYSF
EAVITNEFAGRTMACAPSQLVP----QG-----PGIDPAYQGCALA-GAD
VNAQSVDGSA-YLATQFNYSRSNLWRNFGVVIAFIVLYILVTVIATETVS
FAGGGGGALIFKKSKKAKKQVKHAKHADEEKGGIAEDSSSSSKKNASLGD
APNEDK-----------------------------------EDEALDKLT
KSESIFTWKDVEY---TVPYMG-GERKLLNKVNGYAKPGVMVALMGASGA
GKTTLLNTLAQRQSMGVV----S-GEMFVDGRPLGR--EFQRNTGFCLQG
DLH-DGTATIREALEFSAILRQDASVSREEKIAYVDTVIDLLELNDMQDA
IIS------SLGVEQRKRLTIGVELAAKPSLLLFLDEPTSGLDSQSAYSI
VRFLKKL-ASAGQAIVCTIHQPSSVLIQQFDMILALNPGGNTFYFGPVGE
NGKDVTKYFSDRGV-DCPPHKNVAEFILETAAKPHK--------------
-------RKDGKKIDWNQEWVESQQAKDVLEEIDGLKQTRSHVS------
--TSQKNKDDEKEFAASTMLQCTELLRRTFRQYWRDPSYLYGKFFVSVIV
GIFNGF-TFWQ--LGNTQQDMQNRMFTAFLIITIPPTIVNAVVPKFYTNM
ALWQAREYPSRIYGYFAFVTAQVVAEIPPAIIGAVLYWVLWYWPTGLPTD
S-------STSGYVFFMTLLFFLFQASWGQWITAFSPSFTVISNVLPFFF
VM-FSLFNGVVRPYASLPVFWRYWMYYVNPSTWWIGGVLAATLDGIPVQC
AETETAHFDA--PPG-QTCASYAGAFAQ-----------SAGGYLLNPQD
----NTNCMYCPLST--GNQY--LAQLNIN-ASDKWRDLGIFVVFVFSNW
FLVYFFIYTVRVKGWTFGFG------------------------------
--PLFG
ALGKGVELIKKPFKKGEKKEQSEE--------------------------

>P78577

-----------------------------MSTLTVGDTALPRESRETMV
SIQGIRIVNYSSRRGFRFHNKIQMSAVPMAGL--LMPQVKEQNEREAESG
FKRRELGVTWQNLSVEVVSADAAVQENFLSQFNVPKLARE---SRNKPPL
-----------RTILDNSHGCVKPGEMLLVLGRPGSGCTTLLKMLANQRL
GYKAVQ---GDV------RYGSMTAK---EA-EQYRGQIV--MNTQEELF
FPSLTVGETMDFATRLKVPNRLPN----GVESPEAYREEYKKFLLQSMGI
SHTVDTKVGNEFIRGVSGGERKRVSIIECLGTRASVFCWDNSTRGLDAST
ALEWTKTIRTMTDVLGLSTIVTLYQAGNGIYDLFDKVLVLDEAKQIYYGP
MTQARPYMET-LDFVC-REGSNVADFLTGVTVPTERKI---RSG-FEA--
RFPRNADAMLEEYNKSAVKADMISEYDYPDSE-----------------
---YAKLRTEDFKQAIAEEKAKQLPKSSP--FTVDFMNQVKICVTRQYQI
LWGDKATFIIKQVSTLIQALIAGSLFYDA----PNNSGGLFVKSGALFFS
LLYNSLLAMA-EVTESFQGR-PVLIKHKSFAFFHPAAFCIA-QIAADIPV
LIFQVTIFALPVYFMVGLEMDAGVFFTYWILVFATTMAMTAVFRACGAAF
KTFDDASKVSGFLISALIMYTGYMIRKPEMH---PWFVWIYWIDPLAYGF
DALLSNEFHGKIIPCVGTNLVP----AG--PGYENAT--TQSCTGVGGSI
PGRNYVTGDD-YL-ASLSYSHGHVWRNFGILWAWWALFVVVTIIATSRWK
GASENGPSLLIPRESVEKHRQHGHRDEESQSNEKTSTKGKSEGVQDSSDI
DNQ---LV
RNTSVFTWKDLCY---TVKTPS-GDRQLLDHVYGWVKPGMLGALMGSSGA
GKTTLLDVLAQRKTAGTI----Q-GSVLVDGRPLPV--SFQRSAGYCEQF
DVH-EPYATVREALEFSALLRQPRTTPREEKLKYVDVIIDLLELHDIADT
LIGR--VGAGLSVEQRKRVTIGVELVSKPSILIFLDEPTSGLDGQSAYNT
VRFLRKL-ADVGQAVLVTIHQPSAQLFGEFDSLLLLAKGGKMVYFGDIGD
NGSTVKEYFGRHGA-PCPPNANPGEHMIDVVSGSLS--------------
-------Q--G--RDWHEVWKASPEHTNAQKELDRIISEAGSKP------
---PGTVDD-GHEFAMPLWQQTVIVTKRTCLGVYRNTDYVNNKLALHIGS
ALFNGF-SFWK--MGASVGELQFKLFVLFNFIFAAPGGIGQVQALFIERR
DIYDAREKKSRIFSWVGFVTGLIVSELPYLVLCAVLYFVCFYYQTGLPTS
SD-------KAGAVFFVMLLYEGLYTGIGQFISAYAPNAVFATLTNPLVI
GT-LVSFCGVLVPYGQIQEFWRYWIYWLNPFNYLMGSLLTFTIFDVDIKC
RESEFATFDP--P-NGSSCIDYLSTIFK---------GWGVSANLINPDA
----TSQCQVCQYTR--GSDY--LYSLNLKDYYYGWRDTAIVALFVLSSY
ALVYGLMKLRTKASKKAEE-------------------------------

>Q96W59

------------------------------MTLQRSSSWGVQTGTGEEVY
RTLSRTFSNRPKEETYPESTSSSDDSTKKAADWGLTEELKQTQQQNENDG
AKDKKLGITWTDLDIKGIGADAAFAENVISQFNIPKKIKE---GRQKPPL
-----------KTIVDKSHGCVKPGEMLLVLGRPGAGCTSLLKILANRRL
GYAEID---GDV------KYGSMDHK---QA-QQYRGQIV--MNTEEELF
FPTLTVGQTMDFATRMKVPYNVPS----NFSSAKELQQAQRDFLLKSMGI
EHTDDTKVGNEYVRGVSGGERKRVSILETMAARATVVCWDNSTRGLDAST
ALEYTRCVRAMTDVLGLSSIVTLYQAGNGIYELFDKVLVLDEGKEIFYGP
MSQAKPFMED-LGFVC-TDGANVADFLTGITVPTERRI---RDE-YED--
RFPRNADEVRAAYQKSNIKARMEQEYDYSDTE------------------
---EAKTCTQTFCEAVQAEKHKSLPKKSP--LTTSFYTQVQTSVIRQYQL
LWGDKATFFIKQISTVSQALIAGSIFYNA----PANSSGLFIKGGALFFS
LLYNALVAMN-EVTDSFSAR-PILAKHRGFAYYHPAAFCVA-QITADIPI
IIVQVTLLSLPMYWLTGLKPTAAAFFTYWAILFATSMAITAFFRMIGAGC
ATFDAASKVSGFAVSALIMYTGYMLPKPNMH---PWFVWIYWIDPLAYGF
EALMGNEFSNQVIPCANNNLVP----NG--PGYADSA--FQACTGVRGAP
RGSTIVTGEQ-YL-DSLSYSPSNVWRNFGVLWAWWLLFVALTIYFTSNWS
QVSGNSGFLVIPREKAKKAAH--LMNDEEAQPAGMSEKKTAEDKEKDGNV
DSQ---LI
RNTSVFTWKGLTY---TVKTPT-GDRVLLDDVKGWVKPGMLGALMGSSGA
GKTTLLDVLAQRKTEGTI----K-GSILVDGRDVPI--SFQRSAGYCEQL
DIH-EPLATVREALEFSALLRQPRDVPREDKLKYVDTIIDLLEMHDIENT
LIGT--TYAGLSVEQRKRLTIGVELVSKPSILIFLDEPTSGLDGQAAFNI
VRFLRKL-ADVGQAVLVTIHQPSASLFAQFDTLLLLAKGGKTVYFGDIGD
NGQTVKDYFGRYDA-PCPKNANPAEHMIDVVSGTLS--------------
-------K--D--KDWNRVWLDSPEHSAMTTELDRIVSDAASKP------
---PGTLDD-GREFATSLWTQIKLVTNRNNISLFRNNDYTDNKFMLHIGS
ALFNGF-TFWQ--IGNSVQDLQLRLFALFNFIFVAPGVIAQLQPLFLERR
DLYEAREKKSKMYHWSAFVTGLIVSEIPYLVVCAVLYFVCFYYTVGFPAA
SS-------SAGAVFFVMLFYEFIYTGIGQFVAAYASNALFAFLINPFII
SM-LALFCGVLVPYAQIQPFWRYWFYYLNPFNYLMGSLLVFTTWNVPVTC
KTSELAVFDT--PNAGQTCQEYLAGFLQ---------GMGRTSNLLNPQA
----TSGCEVCQYRT--GADY--LYGLNLTKKSDGWRDAGIVVLFAFSSY
GCVYLLMKLRTKASKKAE--------------------------------

>Q2U2C3

-------------MEFHLHDEALPASTAPTEYGDQSGEEFEAYSGKPTLG

VPDNNVREATSAETLAVHGSPHITPPPGRDAEWSMTDQVIRNKERSEAAG
YKKRELGVTWQNLTVEVLAAEAAVKENQFTQYNIIQLIQD---WRRKPPL
-----------KAILQDSHGCVKPGEMLLVLGRPGSGCTTLLKMLANRRE
GYHSVH---GDV------SFGNMNSE---EA-AHYRGQIV--MNTEEELF
YPRLTVGQTMDFATKLKVPAHLPA----ETKSVHDYVAETKQFLLESMKI
AHTADTKVGNEFVRGVSGGERKRVSIIECMATNGSIFTWDNSTRGLDAST
ALEWAKALRAMTNVMGITTIVTLYQAGNGIYNLFDKVLVLDEGKQIYYGP
AASAKPFMED-LGFVY-SDGANVGDYLTGVTVPTERKI---RPG-FEN--
RFPKNAEAILAEYQRSTLYQTMTREYDYPSSD------------------
---AARQRTEEFKESVAWEKAKHLPNSST--LTVGFWDQLIACTIRQYQI
LWGEKSTFLIKQVLSVAMALIAGSCFYNS----PDTTAGLFTKGGAVFFA
LLYNCIVAMS-EVTESFKGR-PVLIKHKSFAMYHPSAFCLA-QITADLPV
LLVQCTLFAVVIYWMTGLKHTAAAFFTFWAILFTTTLCITALFRCIGAGF
STFEAASKISGTAVKGIVMYAGYMIPKGHIK---NWFLELYYTNPFAYAF
QAALSNEFHGQTIPCVGNNLVP----SG--PGYENVSSANKACTGVGGAL
PGADYVTGDQ-YL-LSLHYKHSQMWRNYGVLWGWWGFFAVLTVICTCFWK
GGAAAGASLLIPRE--KLKAHR-AHLDAEAQKEKDPAREKGSGDALTSAD
EGN---LT
HNTSIFTWKNLTY---TVNTPT-GERVLLDNIHGWVKPGMLGALMGSSGA
GKTTLLDVLAQRKTEGTI----K-GSILVDGRELPV--SFQRMAGYCEQL
DVH-EPYATVREALEFSALLRQSRDTPREEKLKYVDTIIDLLELHDLADT
LIGT--VGNGLSVEQRKRVTIGVELVSKPSILIFLDEPTSGLDGQSAYNT
VRFLRKL-ADVGQAVLVTIHQPSAQLFAQFDTLLLLARGGKTVYFGDIGD
NGAAIKQYFGKYGA-SCPIEANPAEFMIDVVTGGIE--------------
-------EVKD--KDWHQIWLESPEHEHMMVELDQLISDAAAKP------
---PGTHDD-GYEFSMPLWDQVKIVTHRMNVALFRNTNYVNNKFSLHIIS
ALLNGF-SFWH--TGPSVSALNLKMFTIFNFVFVAPGVINQLQPLFIQRR
DIYDAREKKSKMYSWVAFVTGLIVSEFPYLCVCAVLYFACWYYCVRLPHD
SN-------RSGATFFIMLIYEFIYTGIGQFVAAYAPNPTFAALVNPLII
ST-LTLMCGIFVPYSQLTVFWRYWMYYLNPFNYVTSGMLVFGMWGAKVTC
NEDEFAIFDP--V-NG-TCGDYLADYMA---------GSGSRINLTNPDA
----TSGCRVCEYRS--GSDF--LTTLNINHYYYGWRDAGICVIFAISGY
ALVFALMKLRTKASKKAE--------------------------------

>B2WSF9

------------------------MVERIVNSSGWSAGIPSATGEEVLRT
LSRTFSGKSRHDEEMERLDEDNSTDADSTKTKVDIWRLAHHVKEFQNNDP
ADSRKLGVTWNNLTVKVVPAEAHIQENFISQFNIFQQIKE---SRQKSGL
-----------RKILDSSSGCVKPGEMLLVLGRPGSGCTTLLKLLANKRK
GYAEIE---GDV------HFGSLTAK---EA-EPYRGSIV--INTEEELF
YPTMTVGKTMDFATRLNVPDTLPK----DAKSREEYRVQFKEFLLESMGI
SHTEETQVGDAFVRGVSGGERKRVSIIETLATRGSVFCWDNSTRGLDAST

ALEYTRALRCLTDAMGLATIVTLYQAGNAIYDMFDKVLVLDEGKQVFYGT
REQARPFMEE-QGFIC-GEGANIADFLTGVTVPSERQI---RPE-FES--
RFPRNNLELEQVYRQSPIKAAMDQELNYPTTE------------------
---EAKSNTQAFREAITLDKSKSLLKSSP--FTVSFQEQVRACIARQYQI
IWSDKATLFIKQGSSFIQALIAGSLFYNA----PDNSSGLFIKGGSLFLA
LLFNALMAMS-EVTDSYAGR-PILAKQKNFAFFNPAAFCIA-QVTADVPI
IFIQVTTFVVVLYWMTALKATASAFFTCWFLVYLTTFVMTAFFRMIGAAF
KNFDAASKVSGFAVTALIVYAGYQLAKPEMH---PWFVWIYWIDPLSYGL
EAMLANEFHDQIIPCVNANLIP----NFL-PEYQNTT--SAACAGVRGAL
PGATSVLGDD-YL-AGLSYSHDNVWRNVGILFAWWFLFVALTIFFTLGWD
DAAGSGGSLVIPRENRKIAQHA-SQRDEEAQVTEKAPAHDGSGTGNSQSL
GAN---LI
RNTSVFTWRNLSY---IVKTPS-GDRTLLDNVHGYVKPGMLGALMGSSGA
GKTTLMDVLAQRKTEGTI----H-GEILVDGRPLPV--SFQRSAGYCEQL
DVH-EAFSTVREALEFSALLRQSRDTPRAEKLAYVDTIIDLLELRDLEHT
LIGR--LGAGLSVEQRKRVTIGVELVSKPSILIFLDEPTSGLDGQAAFNT
MRFLRKL-ADVGQAVLVTIHQPSAQLFAQFDTLLLLAKGGKTVYFGEIGE
NAKTIKEYFARYDA-PCPPNANPAEHMIDVVTGAHG--------------
-------------KDWNKVWLESPEAEKMHRDLDHIITEAAGKE------
---TGTTDD-GHEFAIDLWSQTKLVTQRMNISLYRNIDYTNNKLALHIGI
ALFIGF-TFWQ--IGDSVSEQSILLFALFNYVFVAPGVIAQLQPLFIERR
DLYETREKKSKMYSWVAFVTGLIVSEIPYLILCAIAYFLCSYYSQGLPSG
SD-------KAGAVFFVMLAYQFMYTGIGQFVAAYAPNPVFASLVNPLLL
GT-LTCFCGVLVPYAQIQEFWRYWMYWLNPFNYLMGALLVFTDFDREIKC
TDSEFATFDP--P-SGQTCGQYLDAWLN---------GPGARTNLVNPDA
----TMECRVCQYTR--GSDY--LVTVNLNDYYYGWRNAAICVIFVFSSY
GLVYLMMKLRTKVSKKAE--------------------------------

>Q55DQ2

-------------MVHNNDGDNNDQEMQEIGINEKRSQFPHISVEESRQE
FDSFSNNIEGESKQFGMNRDAESNMAVESEEDFKLRKYFENSNRMHLENG
GNEKKMGVSIRNLTVVGLGADASVIADMSTPFYGLVKLFTPSFWTKKTST
-----------FDILHDVTTFCKDGEMVLVLGRPGAGCSTLLRVIANQTA
SYVSVK---GDI------TYGGIPSK---EF-EKYRGEPI--YTPEEDSH
HPTLTVRETLDFALKCKTPGNRLP----DE-TKRSFRDKVFNLLLSMFGI
VHQADTIVGNEFIRGLSGGERKRLTITEAMVSSASITCWDCSTRGLDAAS
AFDYAKSIRIMSDTLHKTTIASFYQASDSIYNVFDKVCVLEKGRCIYFGP
VGMAKQYFMS-LGFDC-EPRKSTPDFLTGVTNPQERII---KKG-FEG--
RTPETSADFEEAWKNSDIYRDQLQEQKEYEELI-----------------
---ERTQPKVAFVQEVRDANSKTNFKKSQ--YTTSFVTQVIALIKRNFAL
VLNDKFGMYSKYLSVLIQGFVYASLFYNM----DTDITGLFTRGGAILSA
VIFNAFLSIG-EMAMTFYGR-RVLQKHKSYALYRPSALHIA-QVVTDIPF

TAIQVFLFSIIAYFMFGLQYDAGKFFIFCFTLLGASLACTALFRCFGYLC
PSMYIAQNISNVFIIFMLTYSGYTIPIPKMH---PWFSWFRHINIFTYAF
KALMANEFEGLDFNCK-ESAIP----YG--PAYQGSEFDAYRICPLGGIE
QGSLYFKGDF-YMDKTLSFATGEMSQNVIIVYCWWVFFVVCNMFAMEYID
HTSGGYTHKVYKKG-----------KAPKMNDVEEEKQQNAIVAKATSNM
KDT---LH
MDGGIFTWQNIRY---TVKVPG-GERLLLDNIEGWIKPGQMTALMGSSGA
GKTTLLDVLAKRKTLGVV----E-GDSHLNGRELEI--DFERITGYVEQM
DVH-NPGLTVREALRFSAKLRQEPEVSLEEKFKYVEHVLEMMEMKHLGDA
LIGTLETGVGISVEERKRLTIGVELVAKPQIL-FLDEPTSGLDAQSSYNI
IKFIRKL-ADAGMPLVCTIHQPSSVLFEHFDRILLLAKGGKTVYFGDIGE
KSKTLTSYFERHGVRPCTESENPAEYILEATGAGVH--------------
-------GKSD--VNWPEAWKQSPELADISRELAALKEQGAQQY------
---KPRSDGPAREFSQSTWYQTKEVYKRLNLIWWRDPYYTYGSFVQAALC
GLIIGF-TFWN--LQGSSSDMNQRIFFIFEALMLGILLIFVVMPQLIIQR
EYFK-RDFASKFYSWFPFAISIVVVELPFIVISGTIFFFCSFWTAGLHKT
SD-----DEQTFYFWFIFIIFMFFCVSFGQAVAAVCINMFFAMTLIPLLI
VF-LFLFCGVMVPPSSIPTFWRGWVYHLNPCRYFMEGIITNILKTVRVEC
SEEDMAIFTF--PKSYNTCQNYTSAFQS--------YKPSGYVESATLNG
----EPACGYCIYKN--GEEY--YETLGWS-ADNRWRNVGIIIGFFVFNI
LMVILFVYLTRKGSR-----------------------------------

>B6H8M2

-----------------------------MSETSESPERKIQDREIPVYR
PNGRSLGILFSNITALGGGNSSQTVSDLQKILTDIVMWPIKIVRQLT---
----QGEAHMASSIVEDVSGVIFPGELMLVLGRPGAGCSTVLRLIANQRE
TYQDVH---GCV------EYGGLSSA---EMRTRYRSEVL--YCAEDDIH
FANLSVKDTMDFAMRVRKPYEHHE-------PVTQFSQNMTDSILASLGL
SHTKNTIVGDAFTRGVSGGERRRISLAEVLAVNPVLASWDNPIRGLDSSS
ALSFLDLLRAMSRQTGMASAVTIYQASEAMYEFFDRVMLMYEGKMIFCGP
ATRAKEYFIA-LGFSC-PERQTTADFLTAVTSPSERVF---QDT-YTG--
PRYETAEALARAFRNSKEYQQLQEEMKRYSEQV-----------------
--ASDQSITCSFEEEVRRTRSRFVSKSSS--EISSIWTQSLATSRRQYQL
IWRDWSTLLTVLVLTAVNAVIASSAYYMA----PKTATGSFERSGALFFS
LVYFTLNALT-EVPKTIQSR-AILLKQHRMGYLHPVSFVIA-LAIAEVPV
TALQSIVFACCYYFTIGLEKTAGSFWIFVLIVFVHFTSISTLFRMLGAWS
PNLNIGLLMAGCAVPMVCLYTGYAPPVPTMH---RWGSWIRRISPTPFGM
EALMGNEYSDITLHCSPDQLIP----HGP--GYDDI--HNQGCPMA-GAH
MGSAEVSGKT-YLTSQYGFHAENIWRDFGIILVMWFIYFVLTAVGLSVMT
RESSASNGRVYKRGATSGVHDPRTNDVENQAEGEIKPKANSSASSLTEQV
ADVTVA---EPV

```
SGEGVFVFKDVNY---FVKVAG-EERQLLNDVTGYVKPGQLTALMGASGA
GKSTLLETISGRKSEGRT----E-GSLLFDNRYLDN--TFSRSCGFCMQQ
DVH-EPLATVREALSFSAFMRQTAETTPEQKLEDVDKILELLELDTISDA
LVG------SLGVEERKRVTIGVELCARPSALLFLDEPTSGLDSQAAHSI
VTFLRKI-AGQGVPIVCTIHQPSSIIFEMFDHTLILAPGGRTVYFGETRE
R---LNEYFARNGA-AMSPFANPAEFVITTVAASGQ--------------
-------DPNG--TDWSQLWRNSPEFQKLHKFVSGLIQNAPSSSE-----
GQVQLAPSKNTKEFALPLASQVIHVTTRHWISVWRNGAYNFSKLFKQIFF
QLIVAF-TFFK--VGKDVNGLQNHALAFLIACWVIPALAADLQDIWFQKW
AIFEARER-NGIYDYKALLIALIAVETPWQIVSFTLIFFCSYWTVGYANT
T-------SIGGFVYFMTLLLSLFGTGFCFLVAALFPNPTMAGYATSVFW
VV-LMMFNGIVTPQSVLNSFYRPWIIWADPMRYYFGAIVSSVLHNTEAVC
KQKDFATFHP--PSG-QTCGEYMHNYTS-----------SNPGYLVDPSA
----TSLCQYCPYSD--GDDY--SKTLGFI-YDDRWRDWAVFLAFCLTNL
ALIFFISWLTRVQIRRWRK-------------------------------
--------------------------------------------------
--------------------------------------------------
--------------------------------------------------
-----------------------------
>Q4W9C7
--------------------------------------------------
--------------------------------------------------
--------------------------------------------------
--------------------------------------------------
--------------------------------------------------
--------------------------------------------------
--------------------------------------------------
--------------------------------------------------
--------------------------------------------------
--------------------------------------------------
----------------------------------MDTPSSGTIDLEHGEA
GLRKRLTLTFRSVSVHVTAPDAALGDTLLSVADPRQFLGFLKGSRPK---
-----------RTILKDVSGQVKPGEMLLVLGRPGSGCTSLLRVLSNDRE
SFDEVI---GET------RY-GSMDH---VAARRFRQQIM--FNNEDDVH
FPTLTVNRTMKFALRNKVPRERPD-----GQGSKEFVQEQRDNILSALGI
RHTTKTLVGNEFIRGVSGGERKRVSLAEVIAGQSPIQVWDNPTRGLDSKT
AVEFARLLRREADMNQKTMVATMYQAGNGIYNEFDQVLVLADGRVTYYGP
RQLAKSYFED-MGFVC-PKGANVADFLTSVTVLTERIV---RPG-MED--
KVPSTAEEFEARYRQSDIHQKAMEGFDPPEKL------------------
-----THEVDELTAAVASEKRKRHLPRSPSVYTTSLWEQIQACTIRQFQI
MAGDRLSLIIKVVSAILQALVCGSLFYNL----KDDSSSIFLRPGALFFP
VLYFLLESMS-ETTASFMGR-PILSRQKRFGFYRPTAFCIA-NAITDIPV
VLVQVSCFCIILYFMAALQMDAGRFFTYWIIVIANTLCFMQMFRAVGALC
KRFGNASKITGLLSTIFFVYGGYLIPYEKMH---VWFRWIFYLNPGAYAF
EALMANEFVGKSLQCVQPDYIP----YG--SGYPGSESPYRGCSIP-GS-
-EGDVILGAA-YIRAQYNYSWHHIWRSFGVIIGFWVFFIVLTALGLELLN
S-QGGSSVLLYKRGSQKTRSEDTTTPVQEAARAS----------------
-------------------------------------------------H
AKQSTFTWHDLDY---HVPYQG-QKKQLLDKVFGFVKPGNLVALMGCSGA
GKTTLLDVLAQRKDSGEI----Y-GSILIDGRPQGI--SFQRTTGYCEQM
DVH-EPTATVREALVFSALLRQPAHVPREEKLAYVDHIIDLLELRDISDA
LIGV--PGAGLSIEQRKRVTLGVELVAKP-TLLFLDEPTSGLDGQSAYNI
IRFLRKL-VDGGQAVLCTIHQPSAVLFEAFDSLLLLARGGKMAYFGETGK
DSQTVLDYFARHGA-PCPPDENPAEHIVEVIQGNTD--------------
```

-------KP----IDWVQVWNESEEKQRALAQLQTLNARGKA--------
---DADYVEDTADYATSKWFQFTMVTKRLMVQLWRSPDYVWNKVILHVFA
ALFSGF-TFWK--IGDGAFDLQLRLFAIFNFIFVAPGCINQMQPFFLHNR
DIFEAREKKSKIYHWLAFIGAQTVSEIPYLILCATLYFACWYFTAGFPTT
A-------SISGHMYLQMIFYEFLYTSIGQGIAAYAPNEYFAAVMNPVLI
GAGLVSFCGVVVPFSQMQPFWRDWLYYLDPFTYLVGGLLGEVLWDVEVRC
DPSELVRFRA--PLG-QTCGEYMAAFLA-----------EKPGYLVDGNA
-----TACEFCQYST--GADY--ARTFNLKERYYSWRDTGITALFCVSSY
-AMVFLMMKLRSKKTKSARSE-----------------------------

>P40550

---MSLSKYF
NPIPDASVTFDGATVQLEESLGAVQNDEESASEFKNVG------------
-----------HLEISDITFRANEGEVVLVLGNPT---SALFKGLFHGHK
HLKYSPE--GSI------RFKDNEYK---QFASKCPHQII--YNNEQDIH
FPYLTVEQTIDFALSCKFHIPK------------QERIEMRDELLKEFGL
SHVKKTYVGNDYVRGVSGGERKRISIIETFIANGSVYLWDNSTKGLDSAT
ALEFLSITQKMAKATRSVNFVKISQASDKIVSKFDKILMLGDSFQVFYGT
MEECLTHFHDTLQIKK-NPNDCIIEYLTSILNFKFKET---SNSIVGL--
DTPSVVSEENQALNINNETDLHTLWIQSPYYK------------------
----HWKAITSKTVQECTRKDVNPDDISP-IFSIPLKTQLKTCTVRAFER
IIGDRNYLISQFVSVVVQSLVIGSLFYNI----PLTTIGSFSRGSLTFFS
ILFFTFLSLA-DMPASFQRQ-PVVRKHVQLHFYYNWVETLA-TNFFDCCS
KFILVVIFTIILYFLAHLQYNAARFFIFLLFLSVYNFCMVSLFALTALIA
PTLSMANLLAGILLLAIAMYASYVIYMKDMH---PWFIWIAYLNPAMFAM
EAILSNELFNLKLDCH-ESIIP----RG--EYYDNISFSHKACAWQ-GAT
LGNDYVRGRD-YLKSGLKYTYHHVWRNFGIIIGFLCFFLFCSLLAAEYIT
PLFTRENLLRWNNYLKRYCPFLNSQKKNNKSAITNNDGVCTPKTPIANFS
TSSSSV-------------PSVSHQYDTDYNIKHPDETVNNHTKESVAME
TQKHVISWKNINY---TI-----GDKKLINDASGYISSG-LTALMGESGA
GKTTLLNVLSQRTESGVV----T-GELLIDGQPLTNIDAFRRSIGFVQQQ
DVH-LELLTVRESLEISCVLRGD------GDRDYLGVVSNLLRLP--SEK
LVA------DLSPTQRKLLSIGVELVTKPSLLLFLDEPTSGLDAEAALTI
VQFLKKL-SMQGQAILCTIHQPSKSVISYFDNIYLLKRGGECVYFGSLPN
ACDYFVAHDRRLTFD--REMDNPADFVIDVVGSGSTNIPMDDAEKPTSSK
IDEPVSYHKQSDSINWAELWQSSPEKVRVADDLLLLLEEEARKSG------
-----VDFTTSVWSPPSYMEQIKLITKRQYICTKRDMTYVFAKYALNAGA
GLFIGF-SFWR--TKHNINGLQDAIFLCFMMLCVSSPLINQVQDKALQSK
EVYIAREARSNTYHWTVLLIAQTIVELPLAISSSTLFFLCCYFCCGFETS
A-------RVAGVFYLNYILFSMYYLSFGLWLLYSAPDLQTAAVFVAFLY
SF-TASFCGVMQPYSLFPRFW-TFMYRVSPYTYFIETFVSLLLHDREVNC

STSEMVPSQP--VMG-QTCGQFMKPFID-----------EFGGKLHINNT
----YTVCAYCMYTV--GDDF--LAQENMS-YHHRWRNFGFEWVFVCFNI
AAMFVGFYLTYIKKIWPS--------------------------------

-VIDGIKKCIPSMRRSKTSHNPNEQSV-----------------------

>Q6FQ96

-------------------------------------MEQQDLVENKLSF
SKIPRVGLHVRDLSITASKTDSV---------------------------
-------------LVHPLSLDLPSGSVMAIMGGSGSGKTTLLNVLASKIS
SGLTHSGEISYILEADKNNVDDSSSEASFDLNATYKENVTLAYVPQQDVL
CSRLTCRETLMYAADLKLDASKVE------------KTLIVNQLIDELGL
KDCADTLVGDNSHRGLSGGEKRRLSMGTQMVSNPSVMFLDEPTTGLDAYS
AYLVVKTLKKLAKEDGRTFILSIHQPRSDILFLFDYVCILSKGNVVFCNQ
MNELIPYFKS-ISYDV-PDLVNPADYFIDLSSVDGRT---------ED--
SLAITSERLEMLVSHWRKYEEKTLSYKPINHKY-----------------
-----------------EIQLKSMTGRLP------FWKQVSVLTRRNYKL
NFNDTNTLIATFAEPVVMGAIVGWIYFKP---DTSNIGGLRTLISCLYAS
VILQSYLYLLFDTYRLCEQDIALFDRERAEGAVSGIAFIVARKLALFITD
DILMTILYTSITYFMFGLEKNASKFFFQYAVNLLAQLICSALAMVSVAVS
RDFAKASLVGNLSFTLFSMSCGFFVNAKLMP---VYVRWTKYIAFTWYGF
GSLLSNTFSDSY--C--------------------NGGEGFECQ------
-GNQVVR----------QYGFPLHWRTLPM---WILFCWFVGFFMLSIII
LTWNKVDITLQNEVNKKKHKKSDIKDDASNLTEESDIKDTDDSISTNQRD
DMTNKL-------------------------------------AHITVVA
QDVDLEVKYTKLYNRKNHKIFEYENKKILQNVSAVFKPGMINAIMGPSGS
GKSSFLNLISGRLESSLLVKFNTAGTIKLNDTPISQ-DMFKHLCSYVSQD
DDHLLAMLTVRETFDFAAALRL-KHLSKDDKRSRTDSLIAILGLKHCENT
IIGNEFI-KGISGGEKRRVSMGIQLLSDRPILL-LDEPTSGLDSFTSSTI
LEILENLCSEHNKTVILTIHQPRSELFMKFGNILLLAKSGRTAFNG----
SPEEMIKHFDLMGY-KCPSFTNIADFFLDLISVNTQN-------------
--EQNEIHSKTRVENILSTWRAERLRLSAEKSIVTEEEHTLET-------
---FEDEYGQYFSKPAGLKLAYMVNLRRQYTTTIRNFDSLMARIAQVPGL
GGIFAL-YFAP--LKHNYTSISNRLGLAQESTSLYFVGMLANLAVYPPER
D-YFYEEFKDGVYGVAPFYLAYMTLELPLTTIAAIVYSALTVFGCGMPRT
A-------GNFFANVYCALMIVSCGEALGIMTNTLFKRPGFVVNCISVIL
SI-GTQLSGLMS----------LHMSRVLKGINYINPVWYTSI--IVINF
AFPDNLNF---------TCKDGPQNA------------------------
----DGSCVLKTGRD----------VLDAYGLHRNTKNFLGVVICVTFCY
RLLAYLMLKAKLEWLKW---------------------------------

